# CrossAttOmics: multiomics data integration with cross-attention

**DOI:** 10.1093/bioinformatics/btaf302

**Published:** 2025-05-13

**Authors:** Aurélien Beaude, Franck Augé, Farida Zehraoui, Blaise Hanczar

**Affiliations:** Université Paris-Saclay, Univ Evry, IBISC, Evry-Courcouronnes 91020, France; Sanofi R&D, Translational Precision Medicine, Vitry-sur-Seine 94400, France; Sanofi R&D, Translational Precision Medicine, Vitry-sur-Seine 94400, France; Université Paris-Saclay, Univ Evry, IBISC, Evry-Courcouronnes 91020, France; Université Paris-Saclay, Univ Evry, IBISC, Evry-Courcouronnes 91020, France

## Abstract

**Motivation:**

Advances in high throughput technologies enabled large access to various types of omics. Each omics provides a partial view of the underlying biological process. Integrating multiple omics layers would help have a more accurate diagnosis. However, the complexity of omics data requires approaches that can capture complex relationships. One way to accomplish this is by exploiting the known regulatory links between the different omics, which could help in constructing a better multimodal representation.

**Results:**

In this article, we propose CrossAttOmics, a new deep-learning architecture based on the cross-attention mechanism for multiomics integration. Each modality is projected in a lower dimensional space with its specific encoder. Interactions between modalities with known regulatory links are computed in the feature representation space with cross-attention. The results of different experiments carried out in this article show that our model can accurately predict the types of cancer by exploiting the interactions between multiple modalities. CrossAttOmics outperforms other methods when there are few paired training examples. Our approach can be combined with attribution methods like LRP to identify which interactions are the most important.

**Availability and implementation:**

The code is available at https://github.com/Sanofi-Public/CrossAttOmics and https://doi.org/10.5281/zenodo.15065928. TCGA data can be downloaded from the Genomic Data Commons Data Portal. CCLE data can be downloaded from the depmap portal.

## 1 Introduction

Precision medicine aims to provide individualized clinical decisions according to the unique expression of different biological molecular layers, known as omics profile. With the recent advances in high-throughput methods, it is now possible to simultaneously measure those multiple layers from a single patient. Complex diseases like cancer are often the result of the disruption of multiple levels of omics, such as genomics, transcriptomics, epigenomics, and proteomics. Each omics level only provides a partial view of the underlying complex biological process and disregards significant molecular interactions. By analyzing multiomics data, one can better comprehend the disease and leverage the complementarity and redundancy between individual omics data to improve the predictions. Omics data can be noisy, and exploiting the redundancy between omics helps to increase robustness to noise (Stahlschmidt *et al.* 2022).

Deep learning approaches can capture complex non-linear relationships and interactions between different omics layers without prior selection. Several multiomics integration strategies can be used with different deep learning architectures. The early fusion method combines the different omics in the data space; the resulting vector is analyzed like an unimodal input. This approach is well-studied due to its simplicity and has been successfully applied to auto-encoders (AEs) (Chaudhary *et al.* 2018) and variational auto-encoders (VAEs) (Zhang *et al.* 2019, Benkirane *et al.* 2023) with an unsupervised training. Early fusion has also been applied to multilayer perceptron (MLP) (Huang *et al.* 2019), and MLP whose connection are based on knowledge (Elmarakeby *et al.* 2021, Zhao *et al.* 2021). Graph neural network (GNN) have been used on patient similarity graph (Li *et al.* 2022) or protein-protein interaction (PPI) graph (Althubaiti *et al.* 2021). DeepPathNet is an early fusion strategy based on the self-attention mechanism (Cai *et al.* 2022). However, early fusion does not fully exploit the complementarity between the omics and is known to be sensitive to the differences in distributions across omics.

For the late fusion approach, the fusion occurs in the prediction space. Each omics is processed separately, and the predictions are combined, similar to ensemble methods. In MOGONET, a graph convolutional network (GCN) is used on a patient similarity graph for each modality, and then predictions are combined through the cross-discovery tensor (Wang *et al.* 2021). Errors between modalities should not be correlated to ensure they have complementary effects. However, late fusion approach may not capture complex interactions between modalities by only considering a subset of them. Modalities performing best initially are reinforced, and late fusion models achieves sub-optimal performance.

Thanks to its flexibility, the intermediate fusion approach can help overcome the limitations of early and late fusion. With intermediate fusion, a modality-specific encoder first processes each omics separately. Then, the features learned from each modality are combined in the latent space before being passed to a multimodal classifier (Liang *et al.* 2022). AEs are also used with intermediate fusion strategies (Tong *et al.* 2020), a contrastive loss between the different latent spaces can be used to ensure they are aligned (Sayed *et al.* 2023). MLP have also been combined with an intermediate fusion approach (Sharifi-Noghabi *et al.* 2019, Vale-Silva and Rohr 2021). MOMA (Moon and Lee 2022) is an intermediate fusion strategy where modalities are fused based on their cosine similarity.

The interactions between the different omics layers are patient-specific. The attention mechanism dynamically computes interactions between its input elements, providing flexible modeling capabilities that allow for the inclusion of various and complex modalities, such as omics data (Xu *et al.* 2023). In a multimodal setting, cross-attention can be used to learn relationships between different omics by adapting one modality to another.

The methods described above focused on bulk omics data; recent developments in deep learning have also been proposed in single-cell multiomics integration. Unlike bulk data, single-cell multiomics aims to simultaneously capture multiple molecular layers at the resolution of individual cells. scMM (Minoura *et al.* 2021) employs a multimodal variational autoencoder to learn shared and modality-specific latent representations, capturing cross-modal dependencies. Multigrate (Litinetskaya *et al.* 2022) leverages GNNs to integrate multiomics data through cell-cell similarity graphs, enhancing feature learning across modalities. A comprehensive review of recent advances in single-cell multiomics data integration, including deep learning-based approaches, is provided by (Athaya *et al.* 2023).

In this article, we propose a new intermediate fusion approach based on the attention mechanism to capture interactions between the different omics profiles of a patient in order to predict his phenotype, such as a cancer type. Cross-attention is used to compute modality interactions, and capture their complex relationships, after encoding each modality with an attention-based method, like AttOmics (Beaude *et al.* 2023). We focused the cross-attention computation on known omics regulatory interactions to reduce the number of omics interactions to consider and therefore reduce the computational cost of the attention. The architecture was tested on two different datasets, TCGA (Weinstein *et al.* 2013) and CCLE (Ghandi *et al.* 2019), and was compared with state-of-the-art deep learning-based data fusion methods. The results show that our proposed architecture better considers the interaction between the different modalities and improves model performances, especially with few paired training examples.

## 2 Model architecture

**Figure 1.**
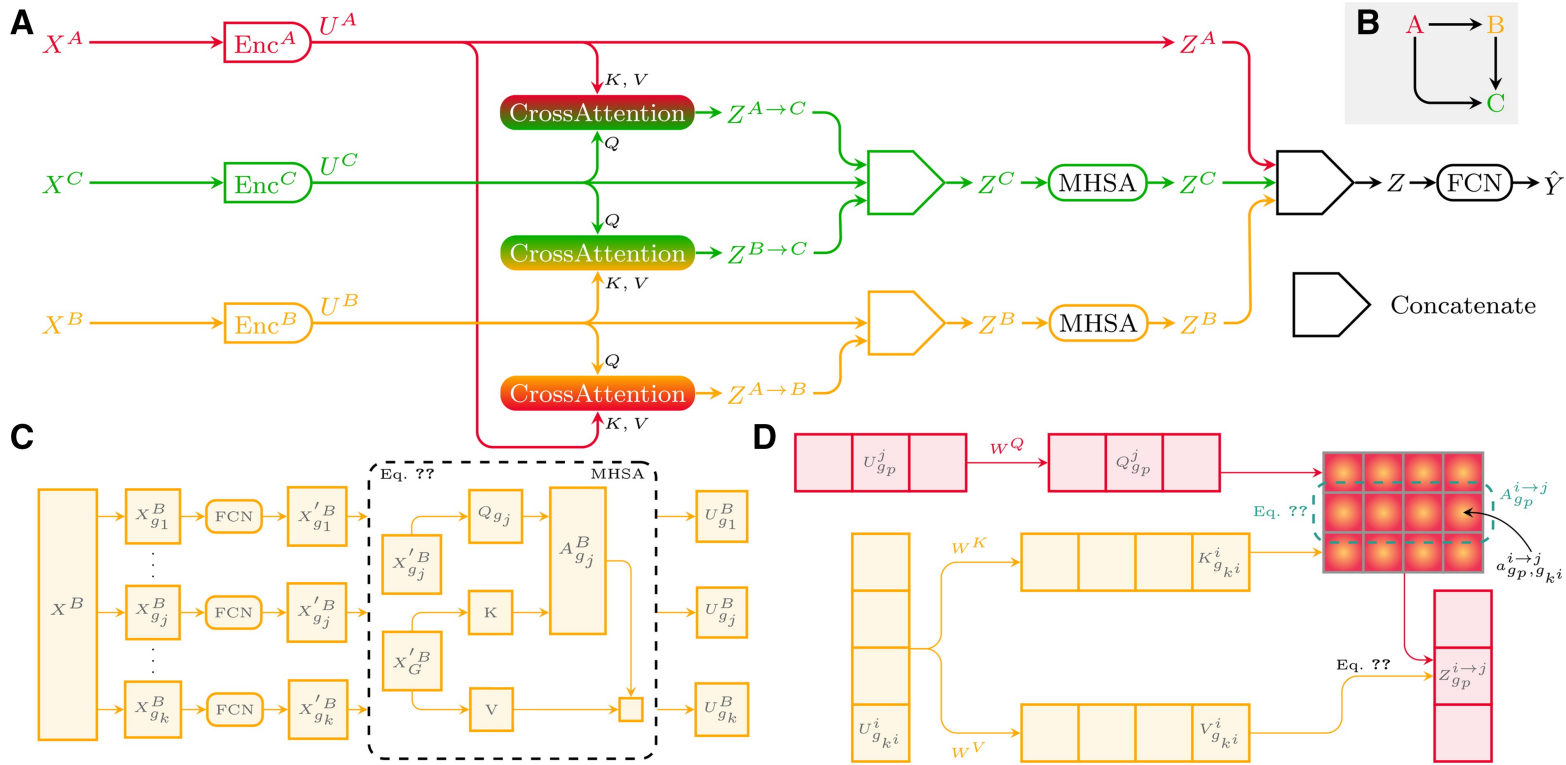
The CrossAttOmics architecture is composed of modalities encoders, cross-attention modules and a predictor (A). Each modality is encoded with its specific attention based encoders capturing intra-modality interactions (C). Modality interactions are computed with cross-attentions (D) according to the modality interaction graph (B).

Our proposed architecture consists of two main components, illustrated in Fig. 1. Initially, a series of encoders independently projects each modality into a representational space, utilizing a self-attention mechanism to capture intra-modality interactions. Subsequently, the architecture employs cross-attention modules to represent modality pairs, focusing on inter-modality interactions. Given the high dimensionality of omics data, direct computation of attention matrices becomes impractical. To address this challenge, we used a strategy of data decomposition into groups, effectively reducing the memory demands associated with attention calculations (Beaude *et al.* 2023).

### 2.1 Modalities encoder

Let $X=\left\{X^{1},\ldots ,X^{M}\right\}$ be a multimodal training example, where *M* is the number of modalities and *Y* is the associated label. We denote with $X^{i}\in R^{p_{i}}$ each modality input, and $p_{i}$ is the number of features for modality *i*. Each modality input $X^{i}$ is encoded with a self-attention-based encoder (Fig. 1C): ${\text{Enc}}^{i}$, based on the AttOmics architecture (Beaude *et al.* 2023). Each modality input $X^{i}$ is randomly split into $k^{i}$ groups $g_{j}$, $1\leq j\leq k^{i}$ [Equation (1)].

(1) $$X_{G}^{i}={\left\{X_{g_{j}}^{i}\right\}}_{1\leq j\leq k^{i}}$$

Each group $X_{g_{j}}^{i}$ is projected into an $s^{i}$-dimensional space with an ${\text{FCN}}^{i}$, a succession of fully connected layers (FCLs), to compute $X_{g_{j}}^{i^{\prime }}$ [Equation (2)]. Each FCL is the composition of an affine transformation of its inputs with a rectified linear unit (ReLU) activation function: FCL(x)=ReLU(Wx+b)=max(0,Wx+b).

(2) $$X_{G}^{{}^{\prime }i}={\left\{{\text{FCN}}^{i}\left(X_{g_{j}}^{i}\right)\right\}}_{1\leq j\leq k^{i}}$$

Multi-head self-attention (MHSA) is then applied to each group $g_{j}^{i}$ ($1\leq j\leq k^{i}$) to compute a new representation $U^{i}={\left\{U_{g_{j}}^{i}\right\}}_{1\leq j\leq k^{i}}$ considering interactions between groups of the *i*-th modality. In each head,

(3) $$U_{g_{j}}^{i}=A_{g_{j}}^{i}\cdot {\left[X_{g_{1}}^{\prime }\cdot W^{V},\ldots ,X_{g_{k^{i}}}^{\prime }\cdot W^{V}\right]}^{T},$$

where $A_{g_{j}}^{i}$ is the attention vector computed by the usual dot product attention (Vaswani *et al.* 2017).

Each modality *i* is represented in a lower dimensional space: $U^{i}={\text{Enc}}^{i}\left(X^{i}\right)$. Combining the different representations makes it possible to exploit their complementarity and redundancy.

### 2.2 Cross-attention: modality interactions

Cross-attention is applied to construct a new representation in which a target modality is reinforced by features from a source modality. This is performed by learning cross-modal interactions between the two modalities. The cross-attention is applied to all pairs of modality defined by the directed interaction graph G=(V,E) (Fig. 1B). Each node v∈V represents a modality, and each edge $\left(u,v\right)\in V^{2},u\neq v$ represents a regulatory link between the two modalities *u* and *v*. The modality *u* is considered the source, and modality *v* is the target.

Let us consider two modalities, a source *i* and a target *j*, i≠j, which are encoded with their respective encoders to obtain the new representations $U^{i}$ and $U^{j}$. Cross-attention is performed with *H* different heads to learn different types of cross-modal interactions. For each head *h*, cross-attention is applied to each group $g_{p}$ of the modalities in order to obtain (Fig. 1D):

$$Z^{i\to j}={\left\{Z_{g_{p}}^{i\to j}\in R^{l^{j}}\right\}}_{1\leq p\leq k^{j}},$$

where $l^{j}=\frac{s^{j}}{H}\in N$. $Z^{i\to j}$ has the same number of groups than the target modality *j*.

$Z_{g_{p}}^{i\to j}$ is defined by:

(4) $$Z_{g_{p}}^{i\to j}=A_{g_{p}}^{i\to j}\cdot {\left[U_{g_{1}}^{i}\cdot W_{h}^{V_{i}},\ldots ,U_{g_{k^{i}}}^{i}\cdot W_{h}^{V_{i}}\right]}^{T},$$

where $A_{g_{p}}^{i\to j}$ is the cross-attention vector, whose n-th element quantifies the attention that the group $g_{p}$ of modality *j* pays to the group $g_{n}$ of modality *i*. It is computed by the usual dot product attention (Vaswani *et al.* 2017).

(5) $$A_{g_{p}}^{i\to j}=\text{softmax}\left(\left[a_{g_{p},g_{1}}^{i\to j},\ldots ,a_{g_{p},g_{k^{i}}}^{i\to j}\right]\right),$$

 

(6) $$a_{g_{p},g_{k^{i}}}^{i\to j}=\frac{{\left(U_{g_{p}}^{j}\cdot W_{h}^{Q^{j}}\right)}^{T}\cdot \left(U_{g_{k^{i}}}^{i}\cdot W_{h}^{K^{i}}\right)}{\sqrt{s^{j}}}.$$

Projection matrix $W_{h}^{Q^{j}}$ maps the group $U_{g_{p}}^{j}$, from an $s^{j}$-dimensional space to an $l^{j}$-dimensional space. Projection matrix $W_{h}^{K^{i}}$ and $W_{h}^{V^{i}}$ maps the group $U_{g_{k^{i}}}^{i}$, from an $s^{i}$-dimensional space to an $l^{j}$-dimensional space. The different heads are fused in an $s^{i}$-dimensional space using a projection matrix $W^{O}$. A residual connection is added to the cross-attention computation to prevent vanishing gradients (Vaswani *et al.* 2017), and cross-attention inputs are normalized using layer-normalization.

### 2.3 Predictor module

All embeddings having the same target modality *j* and $U^{j}$ are concatenated in an unique ensemble $Z^{j}$ (Fig. 1). We then apply MHSA on this ensemble $Z^{j}$ in a similar way as the encoder (Enc) to enrich the multimodal representation from the different modality adaptation (Fig. 1). In each branch, the vectors $Z_{g_{p}}^{i\to j}$ are concatenated into a new vector $Z^{j}$. Modalities *i* with a zero in-degree, (${\text{deg}}^{-}\left(i\right)=0$), in the interaction graph are considered in unimodal branches by taking their unimodal embedding $U^{i}$. Then, the different multimodal $Z^{j}$ and unimodal $U^{i}$ branches are concatenated to form a single multimodal vector *Z*. The vector *Z* is then fed to a fully connected network (FCN) to predict the cancer type $\hat{Y}$ (Fig. 1).

The output layer has one neuron per class, and a softmax activation function is applied to get the probability vector $P={\left[p_{c}\right]}_{1\leq c\leq C}$, where *C* denotes the number of classes.

### 2.4 Model training

We adopt a two-phase training procedure. In the first step, each modality encoder ${\text{Enc}}^{i}$ is trained individually to learn a compact representation of the modality *i* by adding an FCN layer with one neuron per class and a softmax activation function. Each encoder is trained end-to-end with a weighted cross-entropy loss to account for class imbalance:

$$L(\theta^{i})=-\sum_{c=1}^{C}w_{c}Y_{c}\;\text{log}\;\left(\text{FCN}\left({\text{Enc}}^{i}\left(X^{i}\right)\right)\right),$$

where $\theta^{i}$, are the parameters associated with the encoder ${\text{Enc}}^{i}$ and $w_{c}$ denotes the weight (inversely proportional to the class size) of class c∈{1,…,C}

In a second step, we freeze the encoder parameters $\theta^{i}$, and only the multimodal weights of the model, i.e. all the cross-attention and the predictor module weights, are trained. The training is done with a weighted cross-entropy loss:

$$L(\theta )=-\sum_{c=1}^{C}w_{c}Y_{c}\;\text{log}\;\left(p_{c}\right),$$

where θ denotes the multimodal parameters, that is the cross-attention and predictor parameters.

## 3 Experiments

### 3.1 Data

TCGA (Weinstein *et al.* 2013) and CCLE (Ghandi *et al.* 2019) data were used to evaluate our proposed approach CrossAttOmics. For TCGA, we collected DNA methylation (DNAm), gene expression (mRNA), miRNA expression data, copy number variation (CNV), and proteomics for 5862 patients of 18 different cancers from the GDC Data Portal (https://portal.gdc.cancer.gov/). FFPE samples and bad replicates were removed according to the TCGA consortium recommendation. Methylation data was restricted to the probes shared between HumanMethylation27 and HumanMethylation450 platforms. We considered the average of all probes located within 1500 bp of a gene transcription start site as the methylation features. No feature selection was applied, and data were standardized to a zero mean and unit variance. We considered coding and non-coding mRNA (nc mRNA) as two different modalities, as nc mRNAs can affect cancer cell fate through various mechanisms (Grillone *et al.* 2020) and are involved in the oncogenesis (Toden *et al.* 2021).

For CCLE, we collected DNAm, mRNA, miRNA expression data, CNV, metabolomics, and proteomics for 536 cells of 16 different cancers from the DepMap portal (https://depmap.org/portal/ccle/).

Seventy percent of the data was used as a training set, 15% forms the validation set, and the remaining 15% forms the test set while preserving the proportion of each cancer.

### 3.2 Comparative study

**Figure 2.**
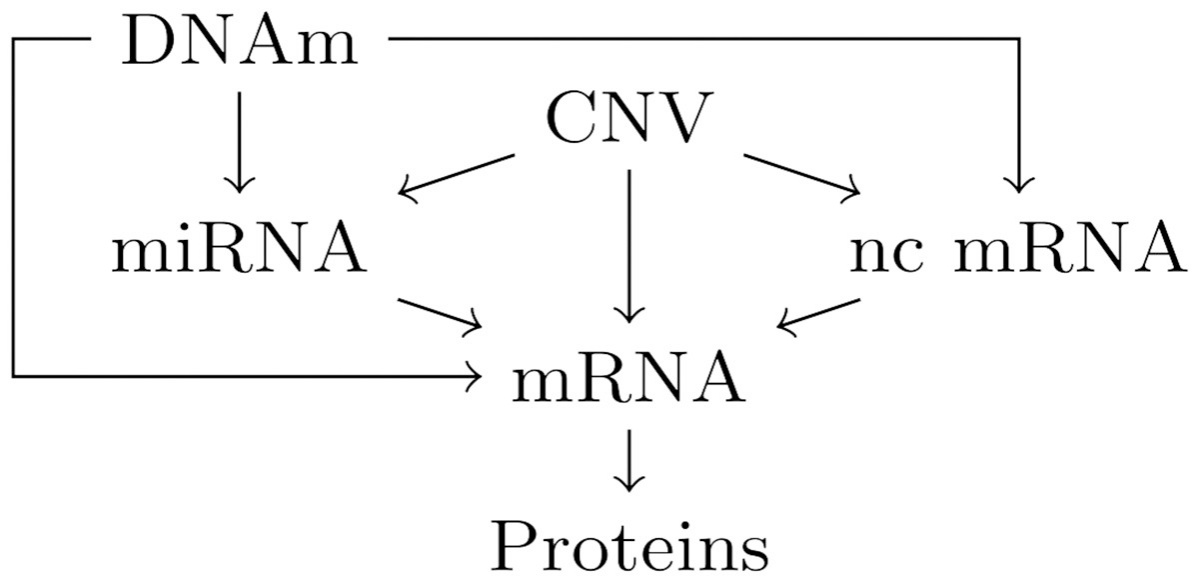
Interaction graph between modalities used for TCGA.

For a comprehensive and comparative evaluation, we chose various deep learning architectures with different integration strategies: MLP early fusion (MLP EF), MLP intermediate fusion (MLP IF), AttOmics early fusion (AttOmics EF), AttOmics intermediate fusion (AttOmics IF), GNN early fusion (GNN EF), P-NET (Elmarakeby *et al.* 2021) and MOGONET (Wang *et al.* 2021). We also considered single-omics architecture for comparison: attention-based model (AttOmics), MLP and GNN based on the PPI graph. We proposed a modality interaction graph based on the known regulations between omics, described in Fig. 2, to train CrossAttOmics on TCGA data. Each edge of the graph represents the computation of a cross-attention between the two modalities composing the edge [Equation (5)]. The details of the different architectures and training hyperparameters are available in the Supplementary File: Section A and Table S2. Attention-based architectures, AttOmics EF, AttOmics IF, and CrossAttOmics, are impacted by the number of groups. We used similar numbers of groups as used in Beaude *et al.* (2023). All models were trained on an Nvidia GeForce RTX 3090.

All the architectures cannot be tested with all possible combinations due to hardware constraints (see below) or architecture limitations. For architecture based on knowledge, not all modalities can be mapped to the knowledge source, for instance miRNA cannot be associated to the PPI graph. Main results are presented on TCGA, similar results are available for CCLE in the Supplementary File.

## 4 Results

### 4.1 Omics combination

**Figure 3.**
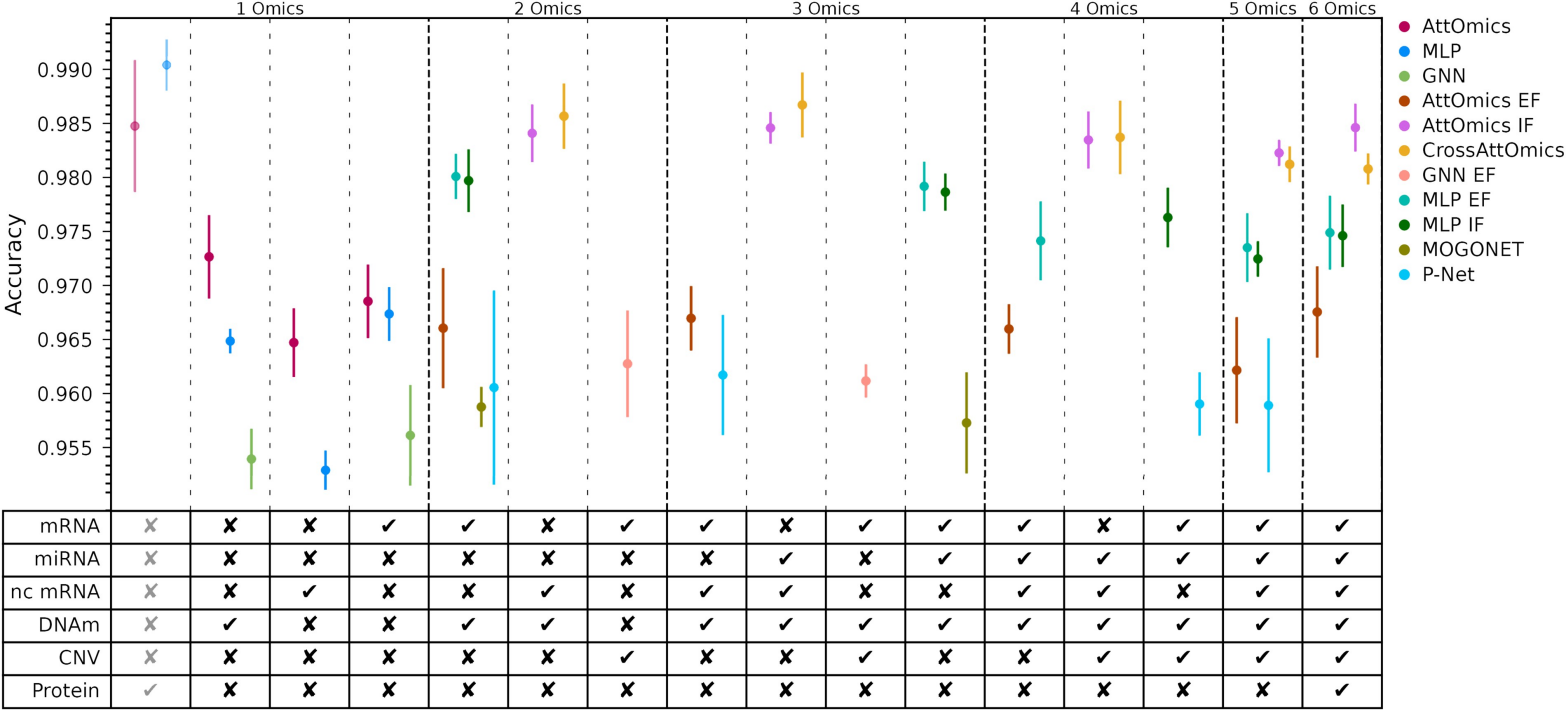
Comparison of the test accuracy of different multiomics deep learning integration models across different omics combination on the TCGA dataset. Each dot represents the mean accuracy obtained by a model on the test set after 5 different training. The error-bars represents the standard-error. For each combination a ✓ means that the omics is included in the combination and a ✗ means that the omics is excluded from the combination.

Figure 3 shows the average and standard deviation of the accuracy on the cancer-type classification task according to the best omics combination for all tested methods on the TCGA dataset.

For single-omics baselines, MLP and AttOmics achieve better performances than the GNN. Results vary according to the studied omics; AttOmics outperforms the MLP when analyzing DNAm or nc mRNA and performs similarly for mRNA and proteins (Fig. 3). The best accuracy is obtained with the proteins for the MLP and AttOmics. On this modality, the best model is the MLP, as the number of features is reduced (454 features), grouping the features does not offer any computational advantages. The proteins used to construct this dataset were pre-selected as known cancer biomarkers. Therefore, they have higher predictive power than the other studied omics. With their higher predictive power, proteins can cause biases towards this modality. During the training phase, modalities will compete with each other, and only a subset of modalities will win (Huang *et al.* 2022). Experimentally, we noticed that including proteins would significantly boost predictive performances for some architectures (Fig. S2 in the Supplementary File). Furthermore, proteomics is not used for diagnosis due to its high cost (Mundt *et al.* 2023). For those reasons, we decided not to report the best combination that includes proteins in Fig. 3. Complete results are available in the Supplementary File.

Increasing the number of omics available during training can boost performances or have little to no effect on test accuracy. There is no change in accuracy when going from two to three modalities with MOGONET. It is impossible to test this model with a higher number of modalities due to the huge memory requirements of the architecture. The VCDN module, used to perform the integration, requires $C^{2M}$ parameters. For 19 classes and four views, this would represent more than 16 billion parameters. P-Net did not benefit either from adding more modalities, and it even reduced the performance. The combination of DNAm and mRNA has lower performances than the MLP on those individual modalities (Fig. 3). AttOmics EF did not benefit from multiple modalities; the performance of the DNAm and mRNA combination is similar to AttOmics on mRNA only and lower than AttOmics on DNAm. Adding multiple modalities hinders the performance in this case. This architecture did not gain from even more modalities; the performances stayed around the same value. The number of groups has been increased to accommodate large multiomics input, but the architecture is known to be impacted negatively with a large number of groups (Beaude *et al.* 2023). When going from single omics to multiomics, we observe a small accuracy gain for the GNN architecture. However, the performances were still lower than AttOmics or MLP on single omics.

Knowledge-based methods are not among the best-performing methods. Their performance depends on the quality of the knowledge, which can be incomplete or outdated. Incorporating knowledge into the architecture may not necessarily improve performance, but it can enhance the interpretability of predictions.

Surprisingly, for the MLP, the integration strategy, early or intermediate, did not impact the performances. Those models achieve better accuracy with two omics than some single omics baselines but could not outperform models only trained on the proteins. Adding more omics did not improve the performances and even started to degrade the accuracy. The addition of omics brings more noise rather than more information. With two and three omics, CrossAttOmics is the best-performing model. It can achieve similar accuracy to a model trained on proteins with multiple omics that are not proteins. Similarly, AttOmics IF is the second-best model.

When training on the six available omics, AttOmics IF outperforms CrossAttOmics. In this case, AttOmics IF has fewer multimodal parameters than CrossAttOmics and is easier to train.

**Figure 4.**
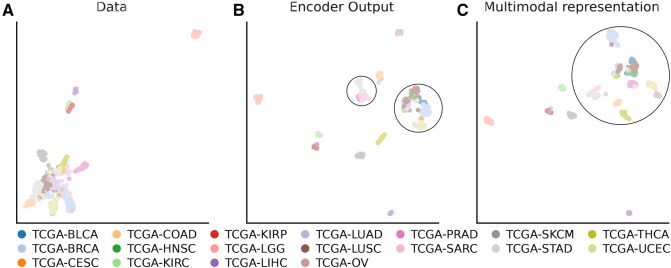
UMAP comparison of pan-cancer samples in the data space (A), after passing through the modality encoder (B) and after applying the cross-attention (C). Each color represents one of the 18 cancers. The multimodal representation of the data has better class separation compared to the raw data and the encoders’ outputs.

To visualize the segregation capabilities in the raw data space, after encoding individual modalities and after enriching individual representation with multiomics information, we reduce the dimension of each patient representation to a 2D space using a UMAP reduction (Fig. 4). Embedding modalities in a latent space with modalities-specific encoders allow for clear identification of cancer clusters, but the separation between some clusters is still unclear (Fig. 4A and B). The addition of multiomics information through cross-attention allows better distinguishing the different cancer type-associated clusters (Fig. 4C). The multiomics embedding can better represent samples from distinct cancers.

Given the quadratic complexity of the attention mechanism, we conducted a comparative analysis of the latency, defined as the time required to obtain a prediction from a single sample, across the various multiomics architectures considered in this study (Fig. S1 in the Supplementary File). It was observed that, for all architectures, an increase in the number of modalities increased the models’ latency. The CrossAttOmics architecture exhibited the highest latency, which can be attributed to its extensive use of attention mechanisms. Nevertheless, the latency remained reasonable, a few dozen milliseconds.

### 4.2 Training with small sample sizes

Despite the broad adoption of high throughput methods in personalized medicine, the availability of paired multiomics data from cancer patients remains limited. Many datasets contain partial multiomics data, where some modalities are entirely missing. Modalities missingness is often the result of economic constraints, experimental limitations, or patient refusal (Kang *et al.* 2021). We explore the impact of the training database size on the performances of CrossAttOmics and other deep-learning architectures by training the different models on a subset of the training set. The different subsets are created by randomly sampling 1%, 2.5%, 5%, 7.5%, 10%, 15%, 30%, 50%, 70%, and 90% of the training set while preserving class proportions. For each subset, five models are trained. The reported performance metrics are estimated on the test set.

**Figure 5.**
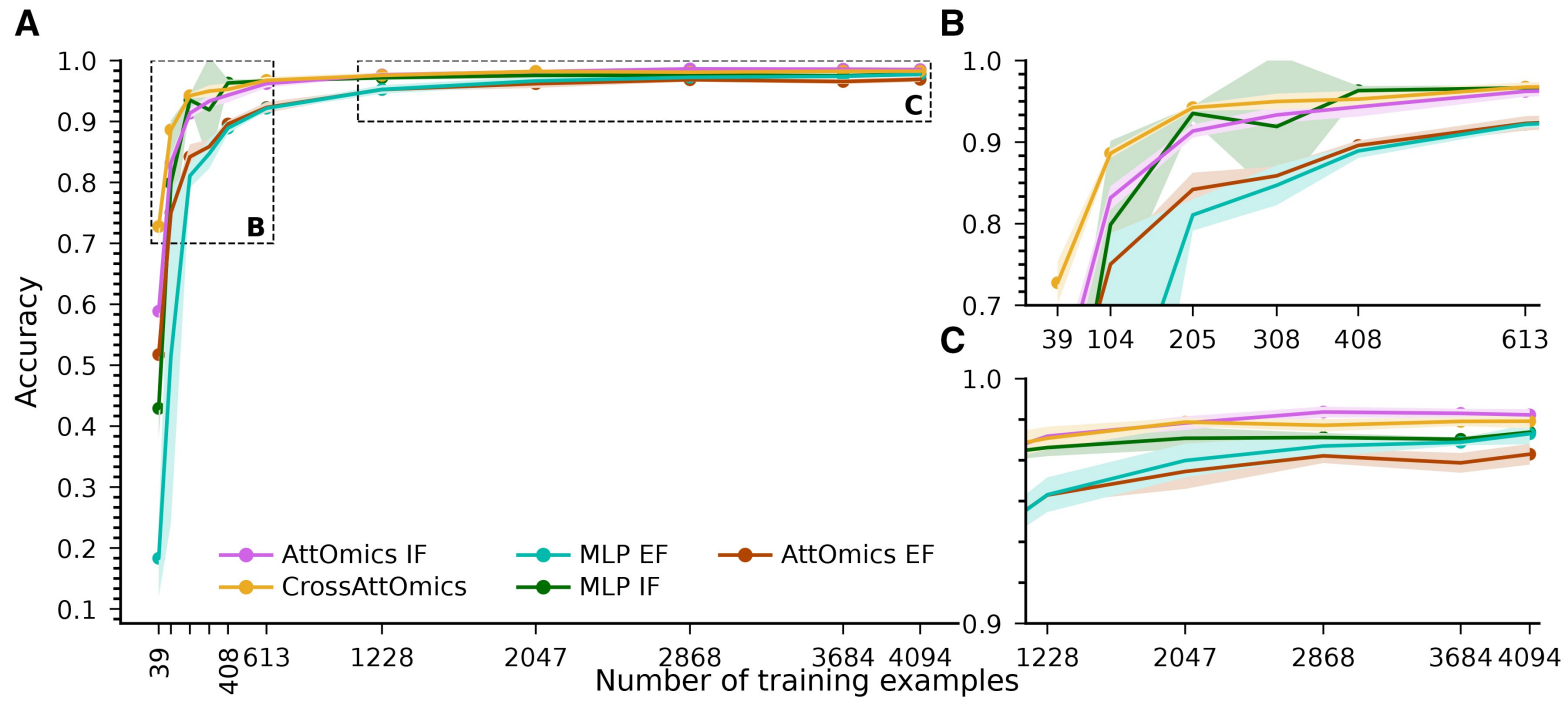
(A) Accuracy on the TCGA test set according to the size of the training set for various multiomics deep learning models when trained on 6 omics. (B) Zoom of panel A focusing on results when training with small datasets. (C) Zoom of panel A focusing on results when training with larger datasets.

Figure 5 shows the average and standard deviation of the accuracy on the cancer-type classification task according to the training set size for all tested methods on the TCGA dataset. The best accuracy is achieved with the highest number of samples. Reducing the number of training examples affects model performance adversely, as a limited training database hinders the capacity of the model to extract hidden information during training.

We observe significant differences between models’ accuracy when training with minimal training datasets. The MLP EF and AttOmics EF are models sensitive to the size of the training set. Despite being easy to implement, early fusion approaches are size-sensitive. On the contrary, the intermediate fusion approach is more resistant to small training sets. Models’ accuracy is lower than those trained on the complete datasets but outperforms early fusion models when trained with less than 600 training examples (Fig. 5B). When trained with less than 300 training examples, CrossAttOmics achieves the best accuracy. Despite CrossAttOmics and AttOmics IF sharing the same encoder, when trained with only 39 examples, CrossAttOmics outperforms AttOmics IF by more than 0.1 points of accuracy. Under limited training settings, cross-attention can improve multimodal representation by allowing modalities to interact and exchange information. [We ran the same experiment using the three best omics for each architecture (Fig. S3 in the Supplementary File).]

### 4.3 Robustness to missing modality

As mentioned earlier, missing modalities in multiomics is a challenge. We circumvent this problem by narrowing our dataset to only complete multiomics samples for the training step. However, in real-world inference scenarios, it is not feasible to exclude patients simply because of incomplete data. The model needs to be robust to different missingness patterns. Although machine learning usually deals with missing data by imputing them, omics artificial generation is still challenging.

A straightforward strategy is to create different missingness patterns during the training (Cheerla and Gevaert 2019). At each iteration, we randomly drop each modality with probability *P*. The maximum number of modalities that can be dropped is a model hyperparameter.

**Figure 6.**
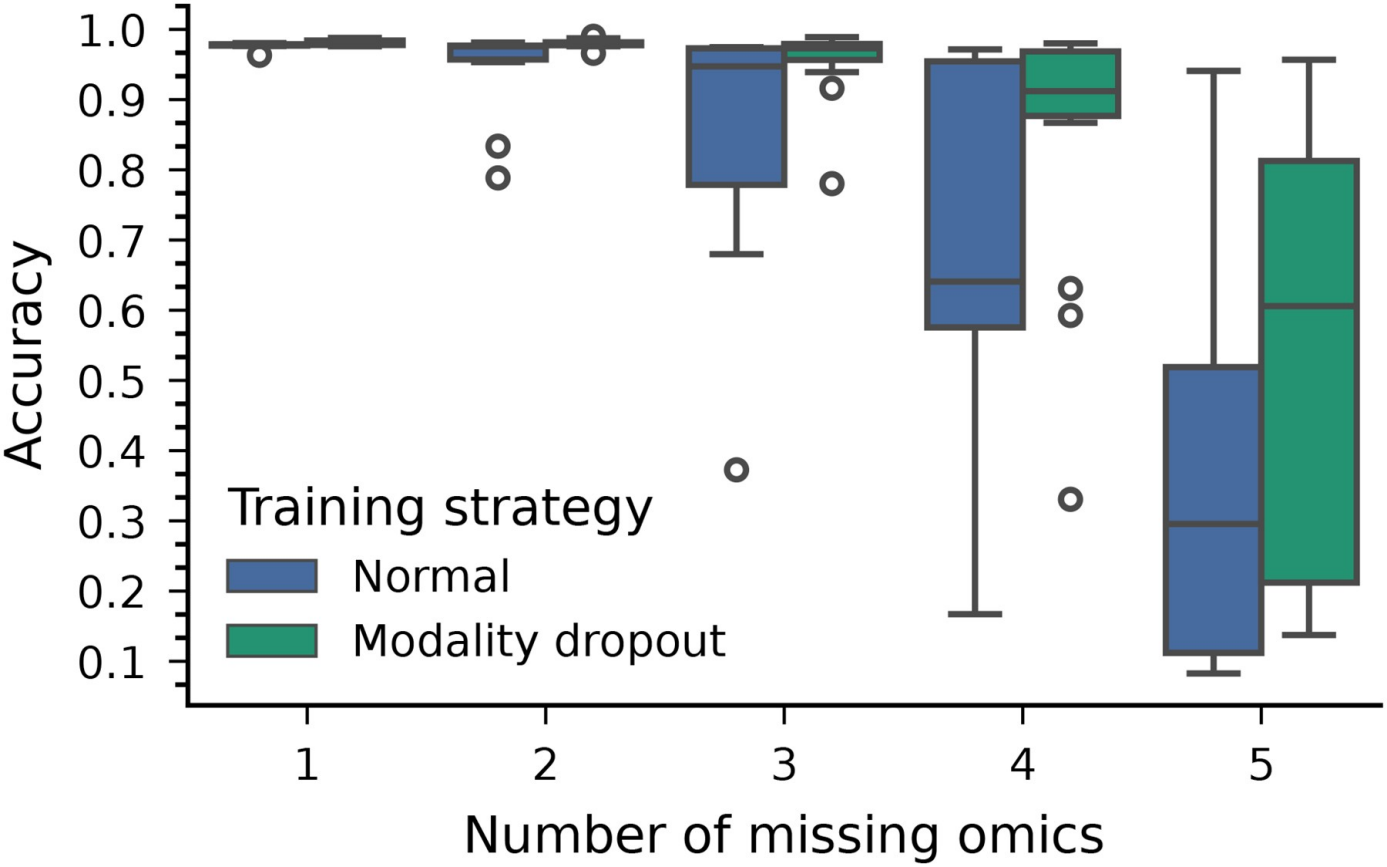
Comparison of CrossAttOmics robustness to missing modalities under two different training strategy: classical training and modality dropout. With modality dropout various scheme of missingness are created during training.

Figure 6 shows the accuracy distribution with different numbers of missing omics under standard and modality dropout training strategies. Each boxplot is calculated with all possible missingness patterns.

With classical training, increasing the number of missing omics degrades the performance. CrossAttOmics is robust to 1 or 2 missing omics, as the multiple cross-attention allows the exchange of information before constructing the multimodal representation. With modality dropout, the robustness is increased, and CrossAttOmics can support up to three missing omics without impacting the accuracy. The performance is slightly impacted when four omics are missing. The impact on the accuracy depends on the omics that are missing. The absence of CNV, an uninformative omic, does not have the same impact as the absence of mRNA, an informative omic. If many of the most informative omics are missing, the model will not be able to restore the missing information from the less informative omics, even by training the model on this specific missingness pattern.

Modality dropout is a simple but effective strategy to increase model robustness to missing modalities. In the Supplementary File, Figs S7 and S8 compare the impact of the different missingness patterns at the cancer level without and with modality dropout.

### 4.4 Interaction importances

Using gradient-based methods to backpropagate the output in the model, we can identify the most significant interactions. One such method is layer-wise relevance propagation (LRP). LRP aims to back-propagate the prediction signal $p_{c}$ in the neural network to assign a relevance score to each neuron. We measure the importance of modality interactions as the mean of the LRP importance scores assigned to the cross-attention output neurons.

**Figure 7.**
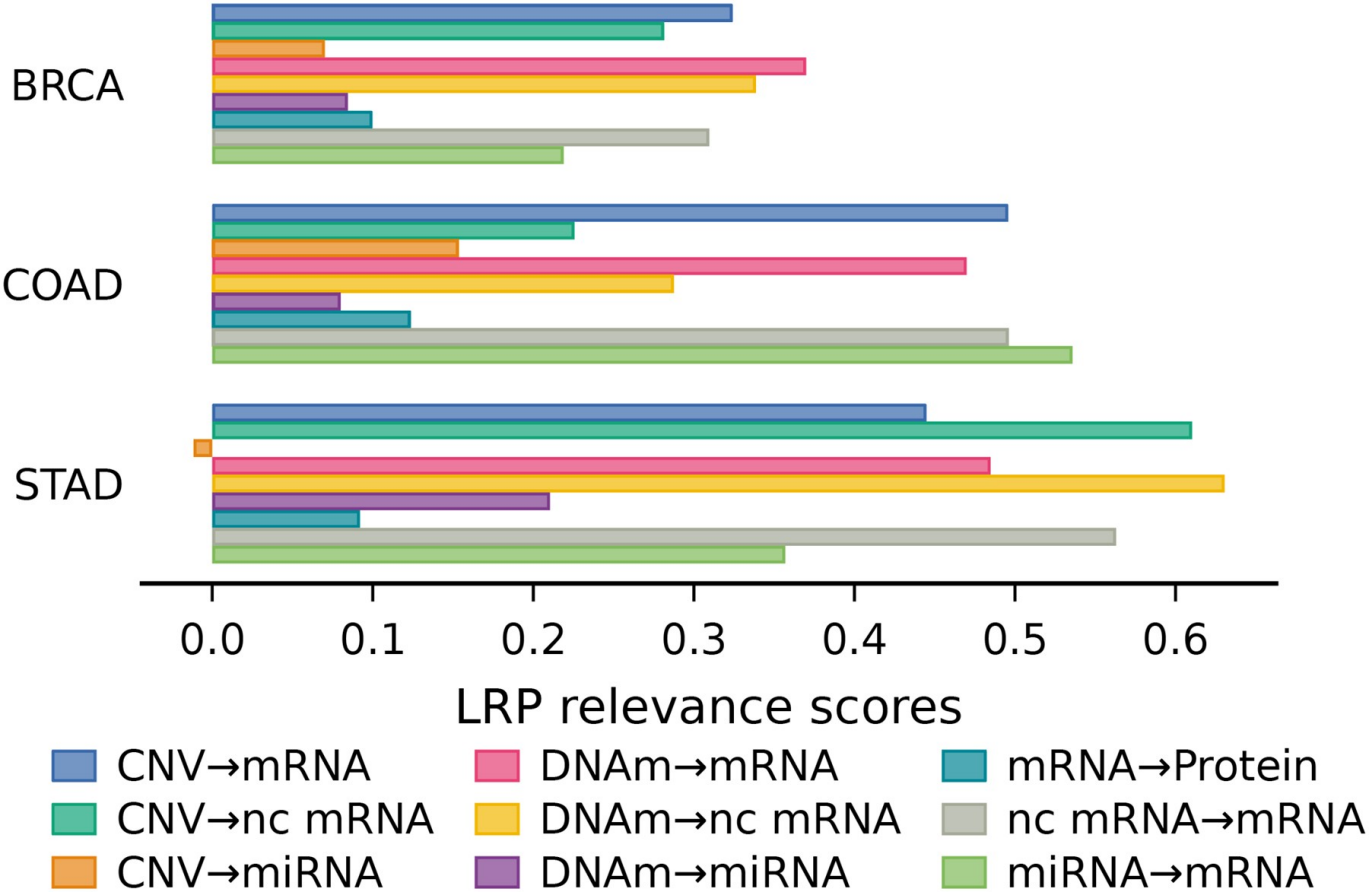
Comparison of the LRP relevance score for the different modelled modality interactions for three cancer.

Figure 7 presents the LRP attribution score for each considered interaction by cancer. Each cancer is characterized by a specific set of important interactions, suggesting that the cross-attention can learn interactions specific to each cancer (Fig. S5 in the Supplementary File). Among the various cancers, the interaction of CNVs with mRNAs stands out as a significant interaction. Combining CNVs and mRNAs makes it possible to exploit their complementarity, and the level of mRNAs confirms the presence of multiple copies of the gene. In the case of colorectal cancer (COAD, Fig. 7), the interaction between miRNA and mRNA has been identified as one of the main interactions for this cancer. miRNAs play a crucial role in gene regulatory networks by targeting various mRNAs (Amirkhah *et al.* 2015). In breast cancer (BRCA, Fig. 7), DNAm–mRNA, DNAm–nc mRNA, and nc mRNA–mRNA interactions were identified as among the most important. Promoter hypomethylation of tumor suppressor genes, such as BRCA1, promotes tumor initiation and progression (Szyf *et al.* 2004). Promoter hypomethylation can also upregulate long non-coding RNA (lncRNA), such as EPIC1, which promotes breast cancer tumourigenesis (Wang *et al.* 2018). In gastric cancer (STAD, Fig. 7), main interactions involve non-coding mRNAs. For this cancer, lncRNAs are known to play key roles in gastric tumourigenesis (Tan *et al.* 2020).

## 5 Conclusions

In this article, we propose CrossAttOmics, a novel deep-learning approach to combine multiomics data. CrossAttOmics harnesses cross-attention to build a multimodal representation that explicitly considers interactions between modalities. While unimodal models trained on specific features, such as proteins in TCGA, can achieve high accuracy, obtaining these features can be challenging and expensive. We show that by using only two or three non-protein omics combinations, CrossAttOmics can achieve similar accuracy to that obtained by training only on proteins. CrossAttOmics outperforms other deep learning architectures when there are very few paired training examples. This is achieved by allowing information to flow between the different omics through the cross-attention layers. By explicitly modeling the interactions between different omics, attribution methods such as LRP can help in identifying the most important interactions.

We adopted a two-phase training strategy, where modalities encoders are trained independently on the prediction task in the first phase. In a second phase the multimodal part, i.e. the fusion through the different cross-attention and the predictor module are trained. There are no restrictions on training the model in an end-to-end fashion, it should be noted that this will increase the training time.

In this work, we only considered omics modalities, but in precision medicine, a wide range of non-omics modalities are available, such as scanners, MRIs, histology slides, or health records. The flexible modeling capabilities of the attention mechanism could help develop a multimodal model integrating heterogeneous and complex modalities.

To assess the significance of modalities interactions, we used post-hoc techniques applied after model training. The generated explanations may not fully capture the underlying complexity of the model, and the explanations may not be reliable. CrossAttOmics could be improved by integrating trainable weights in the architecture to measure the importance of modalities interactions.

This study focused on bulk omics data. Considering single-cell multiomics data could unlock new insights into the intricate interplay of molecular layers in precision medicine. However, attention-based architectures for single-cell multiomics data face challenges due to the complexity and scale associated with such datasets.

Recent technological development added a spatial resolution to single-cell experiments, molecular information is resolved within its tissue context. Methods incorporating the spatial information with the various molecular layers could provide a more holistic understanding of cellular heterogeneity and disease mechanisms.

## Supplementary Material

btaf302_Supplementary_Data
