# Supplementary material for: CrossAttOmics: multiomics data integration with cross-attention
Source: Bioinformatics. 2025 May 13;41(6):btaf302. doi: 10.1093/bioinformatics/btaf302 (PMC12141196; doi:10.1093/bioinformatics/btaf302)
Supplement: btaf302_Supplementary_Data [file btaf302_supplementary_data.pdf]

Supplementary materials for the paper: CrossAttOmics: Multi-Omics data integration with CrossAttention

Aurélien Beaude [1,2,*], Franck Augé [2], Farida Zehraoui [1] and Blaise Hanczar [1,*]

[1]Université Paris-Saclay, Univ Evry, IBISC, 91020, Evry-Courcouronnes, France and
[2]Sanofi R&D, Translational Precision Medicine, 1, impasse des Ateliers, 94400, Vitry-sur-Seine, France

A. Experiments

A.1. Architecture and training details

For a comprehensive and comparative evaluation, we chose various deep learning architectures with different integration strategies: MLP early fusion (MLP EF), MLP intermediate fusion (MLP IF), AttOmics early fusion (AttOmics EF), AttOmics intermediate fusion (AttOmics IF), GNN early fusion (GNN EF), P-NET and MOGONET. We also considered single-omics architecture for comparison: attention-based model (AttOmics), MLP and GNN.

The MLP is based on a FCN with ReLU activations. For the MLP EF architecture, input modalities are concatenanted before being passed to a MLP. For the MLP IF architecture, the modalities encoders were constructed similarly to the unimodal MLP architecture described previously. Unimodal latent representations are concatenated and passed to a 2-layers FCN with ReLU activations.

AttOmics is an attention-based architecture. Input is randomly split into groups, and then each group is embedded in a latent space with a group-specific FCN. Groups are enriched with information from other groups with MHSA. For the AttOmics IF architecture, the modalities encoders were constructed similarly to the unimodal AttOmics architecture described previously. Unimodal latent representations are concatenated and passed to a 3-layers FCN with ReLU activations.

For the GNN architecture the PPI graph is based on data available in the STRING database and was constructed by retaining only high-confidence links: edges with a score higher than 700. We used graph convolutions described in Kipf et al., with a single input and output channel for unimodal training. All the nodes are concatenated and passed to a 2-layers FCN with a ReLU activation. For the GNN EF, the number of channels was set to the number of modalities used for the training.

In all the architectures, the output layer has a softmax activation.

CrossAttOmics is trained using the SGD optimizer with a learning rate of 0.001, a momentum of 0.9, and a batch size of 512. Our 2-phase training allows us to train each encoder with more training examples. An ablation study showed that the MHSA after concatenating the cross-attention outputs was not necessary. When creating the different splits, we paid attention to not including examples used in the pre-training phase in our final test set. The maximum number of epochs is set to 200. An early stopping strategy is deployed to avoid over-fitting with a patience of 10 and a delta of 0.001 on the validation loss between two epochs. Models were evaluated with the accuracy metric; other classification metrics are available in the supplementary file. All models were trained on an Nvidia GeForce RTX 3090.

Thomas N. Kipf, and Max Welling. Semi-Supervised Classification with Graph Convolutional Networks. In *5th International Conference on Learning Representations, ICLR* 2017.

A.2. Multi-head self-attention

In the following, we present the details of the self-attention computation on X'^{i}_{G} to obtain U^i. As the computation is the same for the different modalities, we will omit the modality indicator, U^i will be denoted as U.

MHSA is applied to construct a new representation of the groups, $U = \{U_{g_i}\}_{1 \leq i \leq k}$, by allowing them to interact with each other. MHSA is performed with h different heads to learn different types of interactions. For each head j, self-attention is applied to each group g_i ($1 \leq i \leq k$), in order to obtain: $U^{(j)} = \left\{ U^{(j)}_{g_i} \in \mathbb{R}^l \right\}_{1 \leq i \leq k}$, where $l = \frac{s}{h} \in \mathbb{N}$.

$U^{(j)}_{g_i}$ is defined by:

$$U^{(j)}_{g_i} = A^{(j)}_{g_i} \cdot \left[X'_{g_1} \cdot W^V_j, \cdots, X'_{g_k} \cdot W^V_j \right]^T ,$$

where $A^{(j)}_{g_i}$ is the attention vector computed by the usual dot product attention (Vaswani et al., 2017):

$$A^{(j)}_{g_i} = \operatorname{softmax}\left(\left[A^{(j)}_{g_i,g_1}, \cdots, A^{(j)}_{g_i,g_k} \right] \right) ,$$

$$A^{(j)}_{g_i,g_k} = \frac{\left(X'_{g_i} \cdot W^Q_j \right)^T \cdot \left(X'_{g_k} \cdot W^K_j \right)}{\sqrt{s}} .$$

Projection matrix W_j^Q (respectively W_j^K and W_j^V) maps the group X'_{g_i}, from an s-dimensional space to an l-dimensional space. In the transformers formulation $X'_{g_i} \cdot W_j^Q$, $X'_{g_k} \cdot W_j^K$, and $X'_{g_i} \cdot W_j^V$ are called, query, key, and value respectively. Each element of U_{g_i} is obtained by concatenating the representation of all groups in the different heads and projecting each group to an s-dimensional space using a projection matrix $W^O \in \mathbb{R}^{s \times s}$ as:

$$U_{g_i} = \text{concat}\left(U_{g_i}^{(1)}, \cdots, U_{g_i}^{(h)}\right) \cdot W^O,$$

Algorithm 1 Psuedocode of the CrossAttOmics architecture

Input: M modalities
Input: X^i the representation of modality i
Input: Enc^i the encoder of modality i, θ^i the encoder trainable parameters
Input: $G = (V, E)$ a directed-graph representing the interactions between the different modalities
Output: Trained CrossAttOmics model

Training modalities encoders
for epochs **do**
  for $i = 1$ to M **do**
    $U^i = \text{Enc}^i\left(X^i\right)$ ▷ see eq (2) and (3)
    $\hat{Y}^i = \text{FCN}\left(U^i\right)$
    $\mathcal{L}(\theta^i) = -\sum_{c=1}^{C} w_c Y_c \log\left(\hat{Y}^i\right)$
    $\theta^i \leftarrow \theta^i - \eta_i \nabla \mathcal{L}(\theta^i)$
  end for
end for

Training CrossAttOmics
for epochs **do**
  for $i = 1$ to M **do**
    $U^i = \text{Enc}^i\left(X^i\right)$ ▷ see eq (2) and (3)
  end for
  for (i, j) in E **do** ▷ E is the set of edges in the interaction graph G
    $Z^{i \to j} = \text{CrossAttention}\left(\text{LayerNorm}\left(\text{U}^\text{j}\right), \text{LayerNorm}\left(\text{U}^\text{i}\right)\right) + U^i$ ▷ see eq (4) to (6)
  end for
  for $j = 1$ to M **do**
    $Z^j = \text{Concatenate}\left(\left[U^j, Z^{i \to j} \quad \forall i \in \{1, \cdots, M\} \quad \text{if} \quad (i, j) \in E\right]\right)$ ▷ Modalities with the same target modality are concatenated in a unique vector
    $Z^{j'} = \text{MHSA}\left(Z^j\right)$
  end for
  $Z = \text{Concatenate}\left(\left[Z^j \quad \forall j \in \{1, \cdots, M\}\right]\right)$ ▷ Construction of the multimodal representation
  $\hat{Y} = \text{FCN}\left(Z\right)$
  $\mathcal{L}(\theta) = -\sum_{c=1}^{C} w_c Y_c \log\left(\hat{Y}^i\right)$
  $\theta \leftarrow \theta - \eta \nabla \mathcal{L}(\theta)$
end for

B. Supplementary figures

Table S1. Distribution of the samples across cancer and splits for (a) TCGA and (b) CCLE.

(a)

Cancer	Train	Validation	Test	Total
BLCA	222	48	48	318
BRCA	574	123	124	821
CESC	113	24	25	162
COAD	230	50	50	330
HNSC	228	49	49	326
KIRC	171	37	37	245
KIRP	145	31	32	208
LGG	296	64	64	424
LIHC	118	25	26	169
LUAD	238	51	52	341
LUSC	205	44	44	293
OV	182	39	39	260
PRAD	234	50	51	335
SARC	143	31	31	205
SKCM	227	49	49	325
STAD	226	49	49	324
THCA	254	55	55	364
UCEC	288	62	62	412
Total	4094	881	887	5862

(b)

Cancer	Train	Validation	Test	Total
ALL	16	4	4	24
BRCA	29	6	7	42
COAD-READ	32	7	7	46
DLBC	23	5	6	34
GBM	19	4	5	28
HNSC	20	4	5	29
KIRC	12	3	3	18
LAML	19	4	5	28
LUAD	39	8	9	56
MM	11	2	3	16
OV	25	6	6	37
PAAD	23	5	5	33
SARC	16	4	4	24
SCLC	30	6	7	43
SKCM	32	7	7	46
STAD	22	5	5	32
Total	368	80	88	536

Table S2. Selected hyper-parameters for the omics encoders.

Omics	CNV	mRNA	nc mRNA	DNAm	Proteins	miRNA
# groups	20	20	20	10	20	20
# heads	1	1	1	1	1	1
# layers	4	4	4	3	1	3
embedding dimension	2000	1000	1000	1000	400	1000

Table S3. Main results

mRNA	nc mRNA	miRNA	CNV	DNAm	Protein	AttOmics	GNN	MLP	AttOmics EF	MLP EF	P-NET	GNN EF
					•	0.985 ± 0.006	-	0.990 ± 0.002	-	-	-	-
				•		0.973 ± 0.004	0.954 ± 0.003	0.965 ± 0.001	-	-	-	-
			•			0.727 ± 0.013	0.623 ± 0.017	0.733 ± 0.009	-	-	-	-
		•				0.915 ± 0.004	-	0.926 ± 0.004	-	-	-	-
	•					0.965 ± 0.003	-	0.953 ± 0.002	-	-	-	-
•						0.969 ± 0.003	0.956 ± 0.005	0.967 ± 0.002	-	-	-	-
•				•		-	-	-	0.966 ± 0.005	0.980 ± 0.002	0.961 ± 0.009	0.959 ± 0.007
	•			•		-	-	-	0.964 ± 0.005	0.973 ± 0.002	0.931 ± 0.003	-
	•	•		•		-	-	-	0.964 ± 0.007	0.972 ± 0.001	0.929 ± 0.006	-
•	•			•		-	-	-	0.967 ± 0.003	0.974 ± 0.003	0.962 ± 0.005	-
•	•	•	•	•	•	-	-	-	0.968 ± 0.004	0.975 ± 0.003	-	-

mRNA	nc mRNA	miRNA	CNV	DNAm	Protein	AttOmics IF	MLP IF	CrossAttOmics	MOGONET
					•	-	-	-	-
				•		-	-	-	-
			•			-	-	-	-
		•				-	-	-	-
	•					-	-	-	-
•						-	-	-	-
•				•		0.982 ± 0.001	0.980 ± 0.003	0.982 ± 0.002	0.959 ± 0.002
	•			•		0.984 ± 0.003	0.971 ± 0.001	0.986 ± 0.003	0.951 ± 0.003
	•	•		•		0.985 ± 0.001	0.971 ± 0.003	0.987 ± 0.003	0.944 ± 0.006
•	•			•		0.984 ± 0.004	0.975 ± 0.002	0.983 ± 0.002	0.955 ± 0.004
•	•	•	•	•	•	0.985 ± 0.002	0.975 ± 0.003	0.981 ± 0.001	-

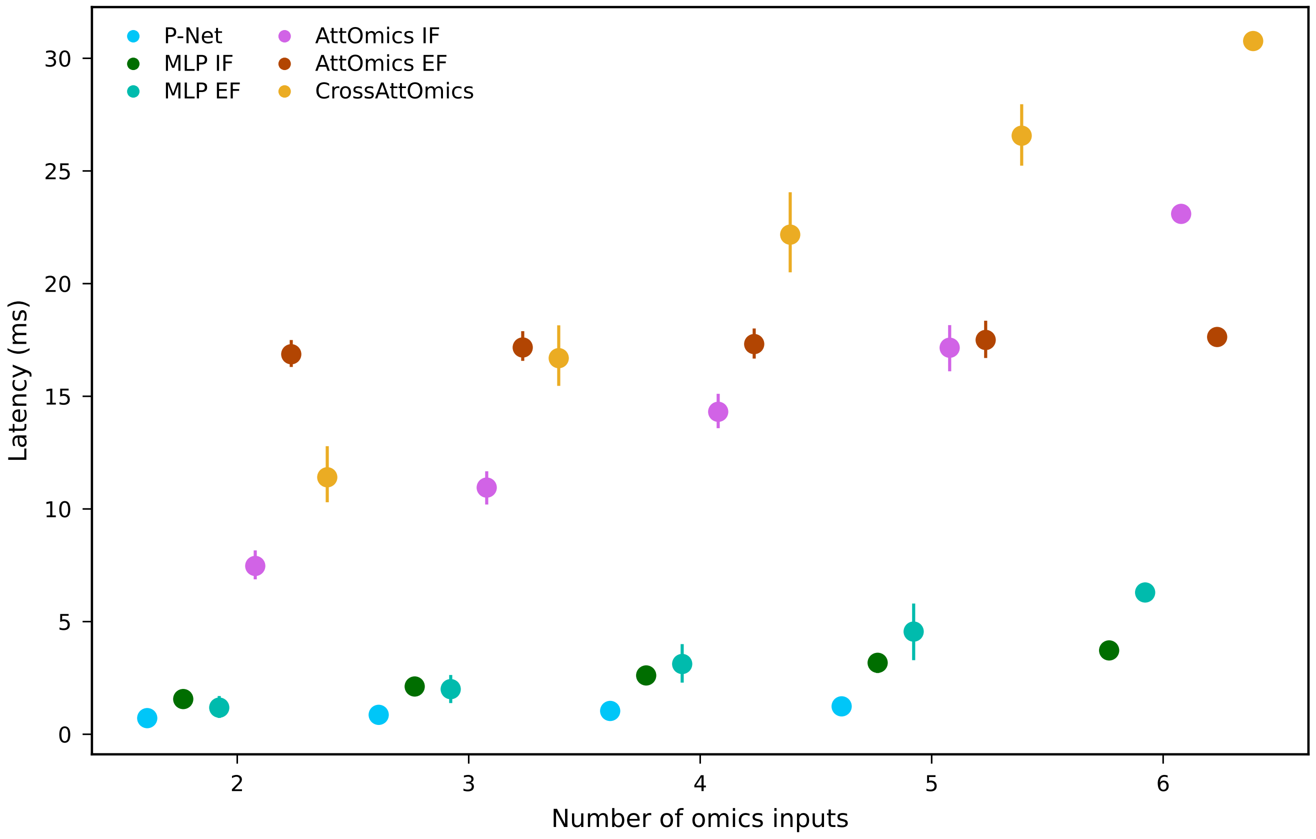

Figure S1: Comparison of the latency, or time in milliseconds to get a prediction for one sample with various number of input omics. Error-bars represent latency variation across the various possible omics combinations.

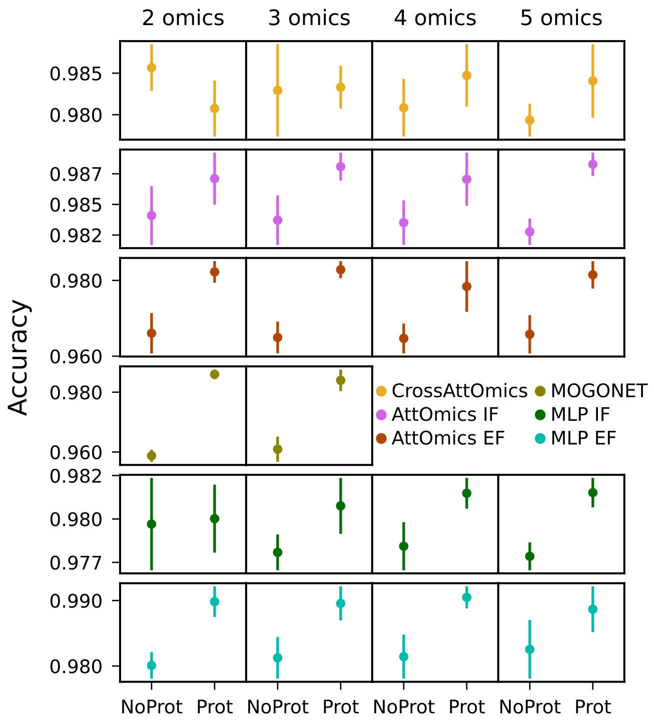

Figure S2: Impact of proteins on the test accuracy when training CrossAttOmics with a fixed number of omics on the TCGA dataset.

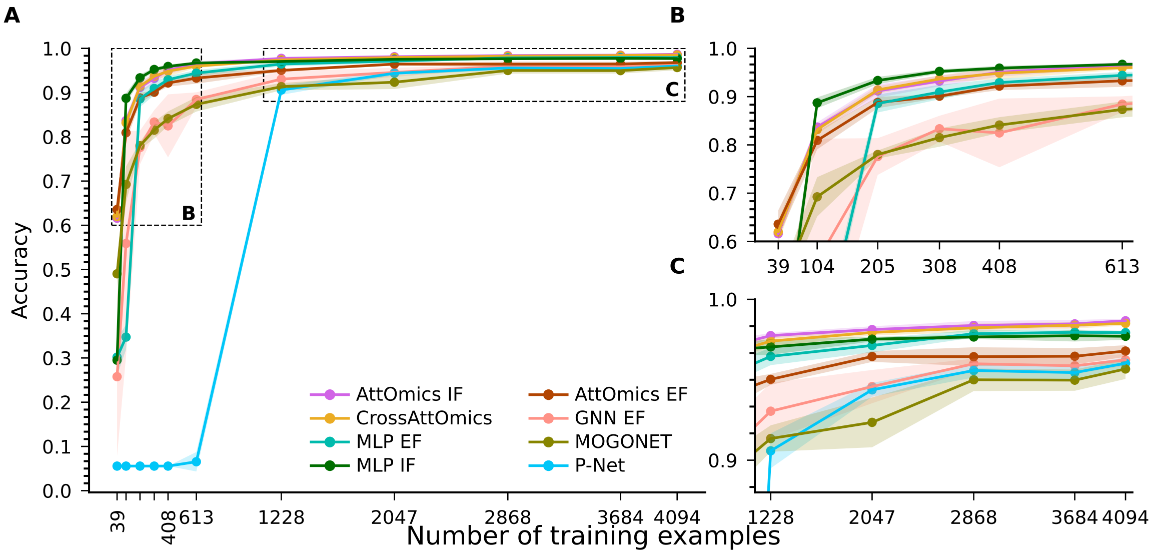

Figure S3: Accuracy on the TCGA test set according to the size of the training set for various multi-omics deep learning models when trained on the best combination of 3 omics. When trained with the minimum samples possible, CrossAttOmics outperformed MLP IF. When the number of training samples was increased, MLP IF became the better architecture. The performance of CrossAttOmics depends on the number of modalities used, which impacts the number of cross-attentions. With the selected omics, miRNA, nc mRNA, and DNAm (Figure 2), there is only two cross-attention to compute. The model cannot benefit from the cross-attention mechanism compared to situations with more modalities (Figure 5).

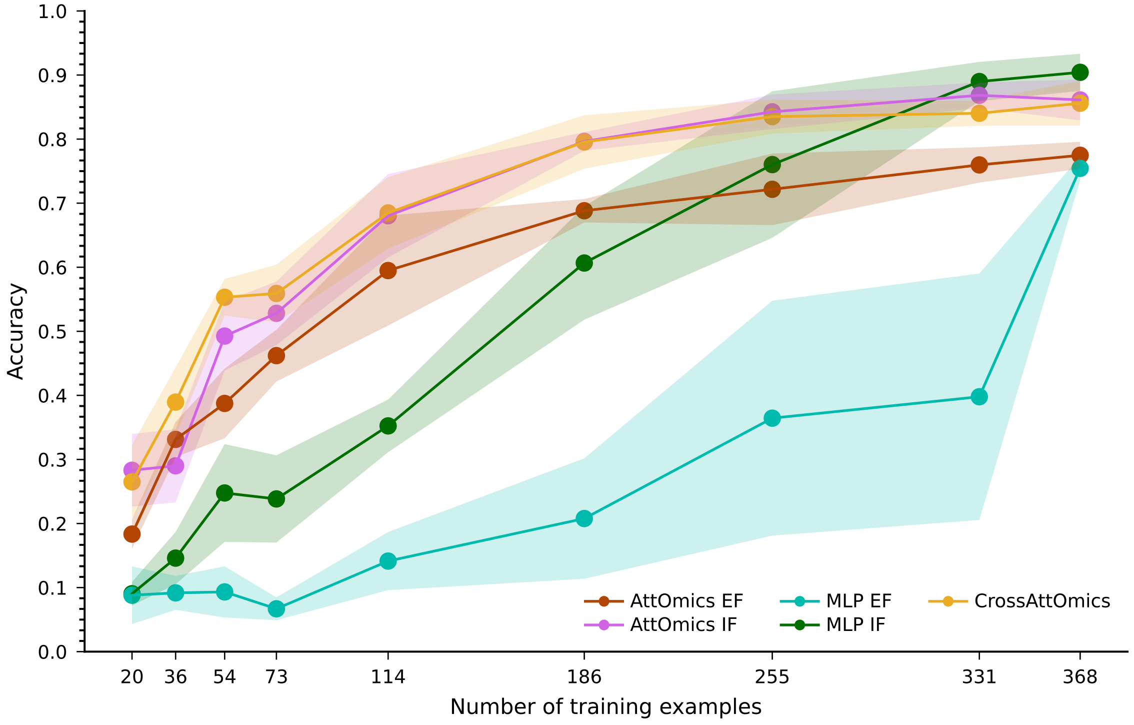

Figure S4: Accuracy on the CCLE test set according to the size of the training set for various multi-omics deep learning models when trained on the best combination of 3 omics.

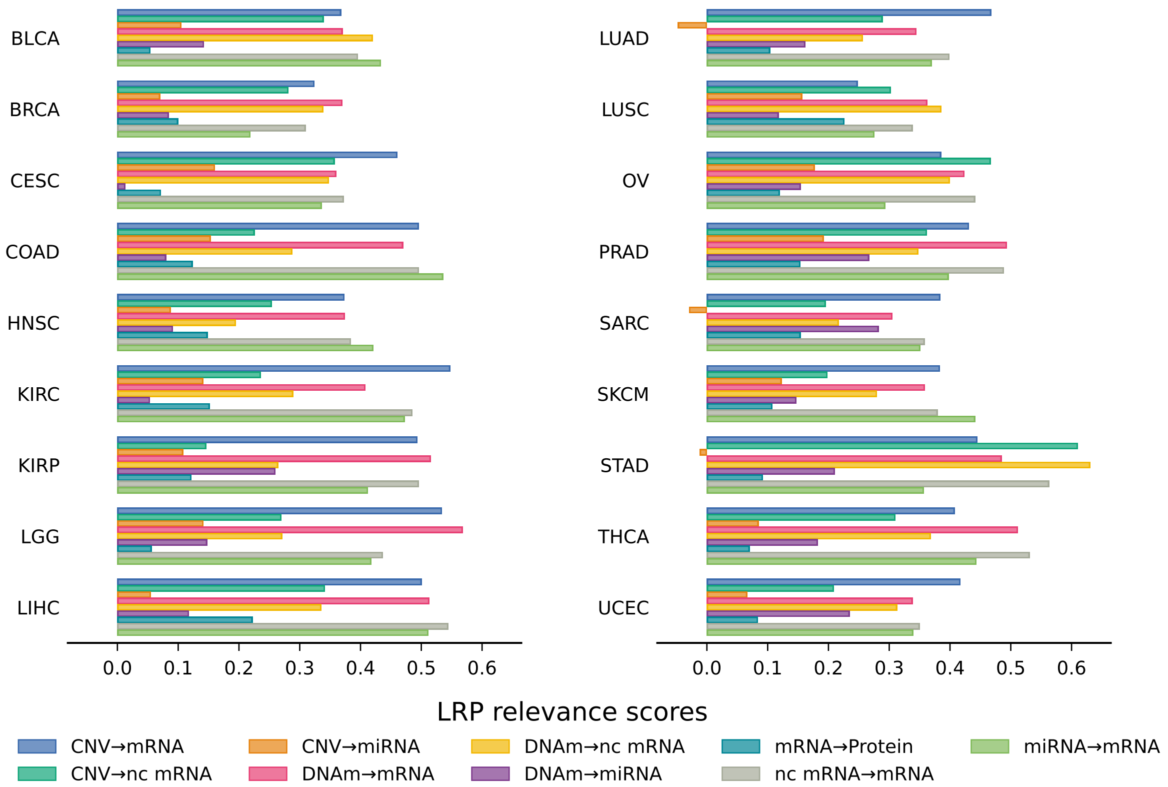

Figure S5: Comparison of the LRP relevance score for the different modelled modality interactions across various cancer.

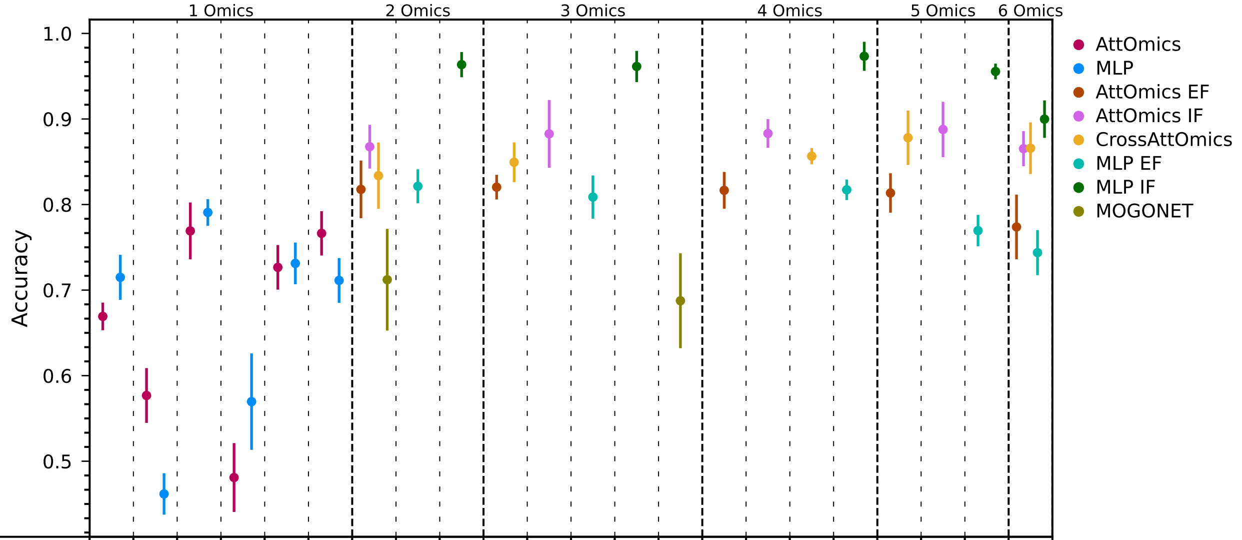

	1 Omics						2 Omics			3 Omics					4 Omics				5 Omics			6 Omics
mRNA	✘	✘	✘	✘	✘	✔	✔	✘	✘	✔	✔	✘	✘	✔	✔	✔	✔	✘	✔	✔	✘	✔
miRNA	✘	✘	✘	✘	✔	✘	✘	✔	✘	✘	✘	✔	✔	✘	✔	✘	✔	✔	✔	✘	✔	✔
Metabolomics	✘	✘	✘	✔	✘	✘	✘	✘	✔	✔	✘	✔	✘	✘	✔	✘	✘	✔	✔	✔	✔	✔
DNAm	✘	✘	✔	✘	✘	✘	✔	✔	✔	✔	✔	✔	✔	✔	✔	✔	✔	✔	✔	✔	✔	✔
CNV	✘	✔	✘	✘	✘	✘	✘	✘	✘	✘	✘	✘	✘	✔	✘	✔	✔	✘	✘	✔	✔	✔
Protein	✔	✘	✘	✘	✘	✘	✘	✘	✘	✘	✔	✘	✔	✘	✘	✔	✘	✔	✔	✔	✔	✔

Figure S6: Comparison of the test accuracy of different multi-omics deep learning integration models across different omics combination on the CCLE dataset. Each dot represents the mean accuracy obtained by a model on the test set after 5 different training. The error-bars represents the standard-error. For each combination a ✓ means that the omics is included in the combination and a ✗ means that the omics is excluded from the combination.

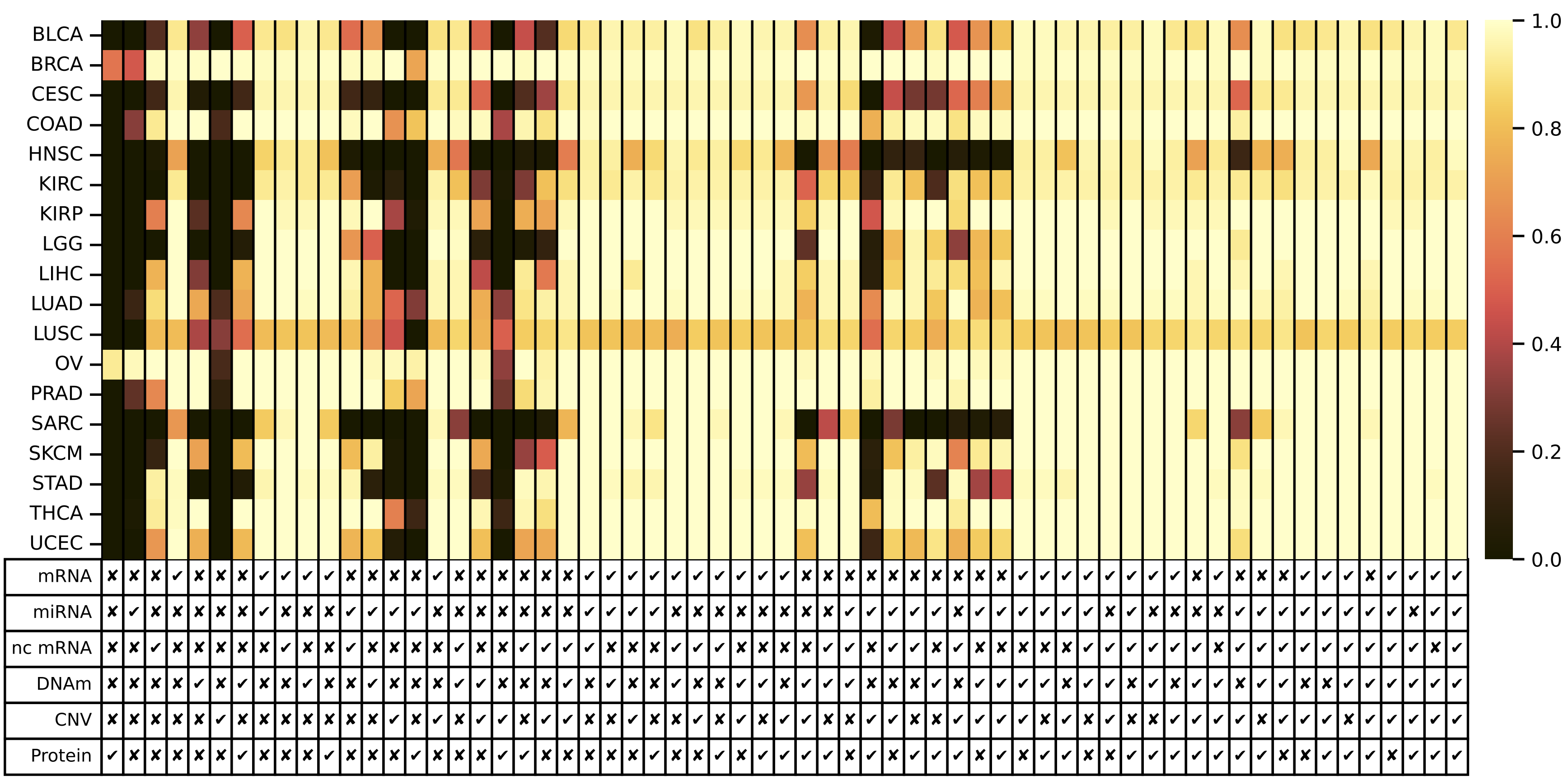

Figure S7: Comparison of the impact of the different missingness pattern on the accuracy per cancer after training the CrossAttOmics model on the 6 omics.

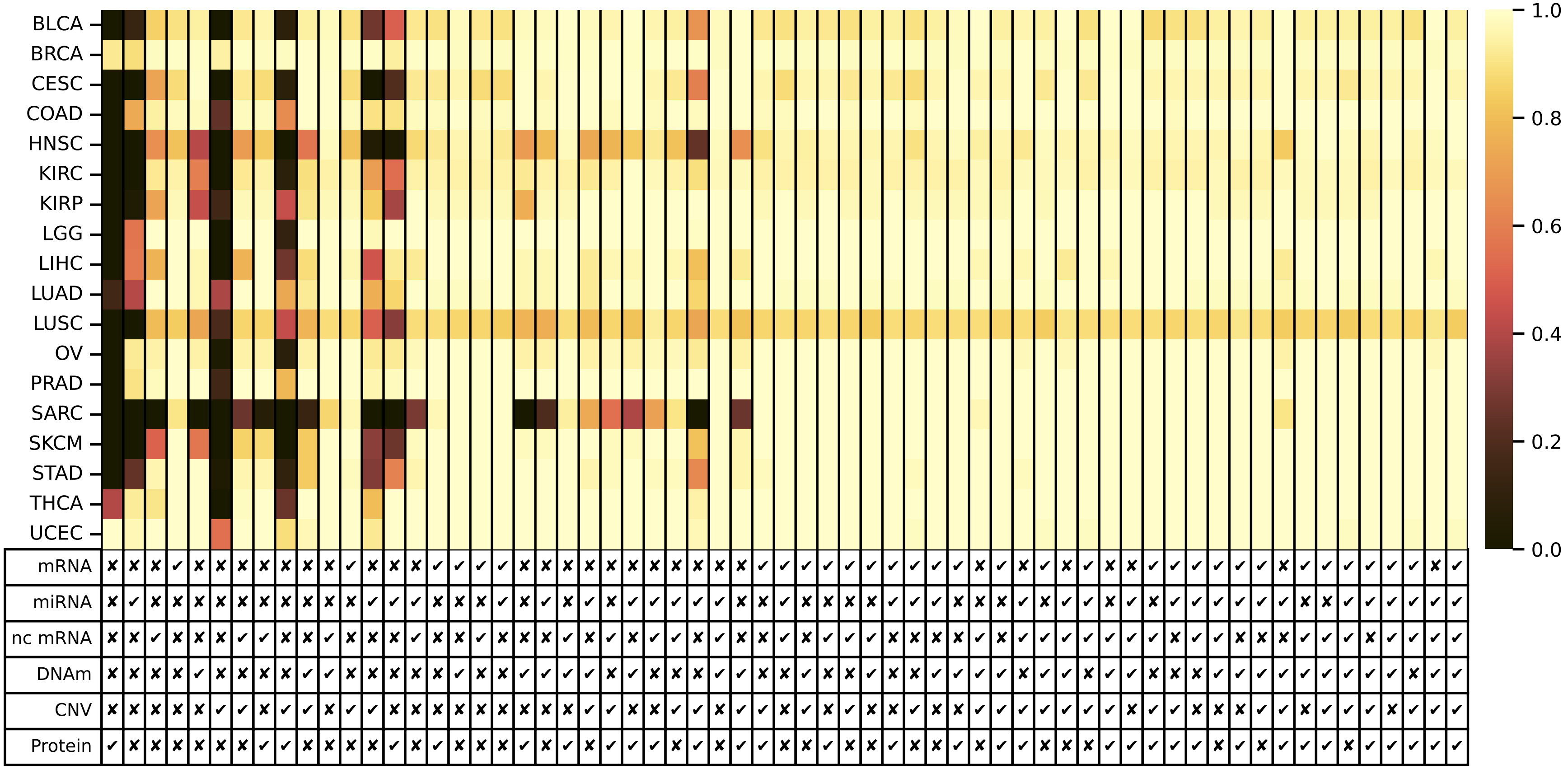

Figure S8: Comparison of the impact of the different missingness pattern on the accuracy per cancer after training the CrossAttOmics model on the 6 omics with modality dropout.

C. Supplementary tables

C.1. Models performances on various combinations of omics on TCGA dataset

Table S4: Classification metrics for AttOmics with different omics combination on TCGA dataset

mRNA	nc mRNA	miRNA	CNV	DNAm	Protein	AUROC	Accuracy	F1	Precision	Recall	Specificity
					•	1.000 ± 0.000	0.985 ± 0.006	0.984 ± 0.006	0.984 ± 0.006	0.985 ± 0.006	0.999 ± 0.000
				•		0.999 ± 0.000	0.973 ± 0.004	0.971 ± 0.004	0.970 ± 0.005	0.973 ± 0.004	0.998 ± 0.000
			•			0.969 ± 0.003	0.727 ± 0.013	0.722 ± 0.015	0.729 ± 0.016	0.727 ± 0.013	0.984 ± 0.001
		•				0.996 ± 0.001	0.915 ± 0.004	0.912 ± 0.004	0.912 ± 0.004	0.915 ± 0.004	0.995 ± 0.000
	•					0.999 ± 0.000	0.965 ± 0.003	0.962 ± 0.003	0.961 ± 0.004	0.965 ± 0.003	0.998 ± 0.000
•						0.999 ± 0.000	0.969 ± 0.003	0.967 ± 0.004	0.967 ± 0.004	0.969 ± 0.003	0.998 ± 0.000

Table S5: Classification metrics for AttOmics EF with different omics combination on TCGA dataset

mRNA	nc mRNA	miRNA	CNV	DNAm	Protein	AUROC	Accuracy	F1	Precision	Recall	Specificity
				•	•	0.999 ± 0.000	0.968 ± 0.003	0.966 ± 0.002	0.966 ± 0.002	0.968 ± 0.003	0.998 ± 0.000
			•		•	0.999 ± 0.000	0.943 ± 0.005	0.940 ± 0.004	0.938 ± 0.003	0.943 ± 0.005	0.997 ± 0.000
			•	•		0.998 ± 0.000	0.947 ± 0.005	0.945 ± 0.006	0.943 ± 0.007	0.947 ± 0.005	0.997 ± 0.000
			•	•	•	0.999 ± 0.000	0.961 ± 0.003	0.959 ± 0.003	0.958 ± 0.003	0.961 ± 0.003	0.998 ± 0.000
		•			•	0.999 ± 0.000	0.966 ± 0.005	0.966 ± 0.005	0.967 ± 0.004	0.966 ± 0.005	0.998 ± 0.000
		•		•		0.998 ± 0.000	0.955 ± 0.005	0.953 ± 0.004	0.952 ± 0.003	0.955 ± 0.005	0.998 ± 0.000
		•		•	•	0.999 ± 0.000	0.968 ± 0.003	0.966 ± 0.003	0.965 ± 0.004	0.968 ± 0.003	0.998 ± 0.000
		•	•			0.990 ± 0.001	0.849 ± 0.009	0.840 ± 0.009	0.837 ± 0.011	0.849 ± 0.009	0.991 ± 0.000
		•	•		•	0.999 ± 0.000	0.944 ± 0.006	0.941 ± 0.007	0.939 ± 0.007	0.944 ± 0.006	0.997 ± 0.000
		•	•	•		0.998 ± 0.000	0.945 ± 0.004	0.943 ± 0.004	0.941 ± 0.005	0.945 ± 0.004	0.997 ± 0.000
		•	•	•	•	0.999 ± 0.000	0.964 ± 0.003	0.962 ± 0.002	0.962 ± 0.002	0.964 ± 0.003	0.998 ± 0.000
	•				•	1.000 ± 0.000	0.982 ± 0.003	0.982 ± 0.003	0.983 ± 0.003	0.982 ± 0.003	0.999 ± 0.000
	•			•		0.999 ± 0.000	0.964 ± 0.005	0.962 ± 0.005	0.961 ± 0.006	0.964 ± 0.005	0.998 ± 0.000
	•			•	•	0.999 ± 0.000	0.974 ± 0.004	0.973 ± 0.004	0.972 ± 0.004	0.974 ± 0.004	0.999 ± 0.000
	•		•			0.997 ± 0.000	0.930 ± 0.004	0.927 ± 0.004	0.925 ± 0.003	0.930 ± 0.004	0.996 ± 0.000
	•		•		•	0.999 ± 0.000	0.954 ± 0.004	0.952 ± 0.005	0.951 ± 0.006	0.954 ± 0.004	0.997 ± 0.000
	•		•	•		0.999 ± 0.000	0.959 ± 0.005	0.958 ± 0.004	0.958 ± 0.004	0.959 ± 0.005	0.998 ± 0.000
	•		•	•	•	0.999 ± 0.000	0.965 ± 0.005	0.964 ± 0.005	0.963 ± 0.004	0.965 ± 0.005	0.998 ± 0.000
	•	•				0.999 ± 0.000	0.958 ± 0.007	0.958 ± 0.006	0.958 ± 0.006	0.958 ± 0.007	0.998 ± 0.000
	•	•			•	1.000 ± 0.000	0.979 ± 0.001	0.979 ± 0.002	0.979 ± 0.002	0.979 ± 0.001	0.999 ± 0.000
	•	•		•		0.999 ± 0.000	0.964 ± 0.007	0.962 ± 0.007	0.962 ± 0.007	0.964 ± 0.007	0.998 ± 0.000
	•	•		•	•	0.999 ± 0.000	0.973 ± 0.003	0.972 ± 0.004	0.971 ± 0.004	0.973 ± 0.003	0.999 ± 0.000
	•	•	•			0.998 ± 0.000	0.935 ± 0.003	0.932 ± 0.003	0.930 ± 0.003	0.935 ± 0.003	0.996 ± 0.000
	•	•	•		•	0.999 ± 0.000	0.958 ± 0.006	0.955 ± 0.007	0.953 ± 0.008	0.958 ± 0.006	0.998 ± 0.000

Continued on next page

Table S5: Classification metrics for AttOmics EF with different omics combination

mRNA	nc mRNA	miRNA	CNV	DNAm	Protein	AUROC	Accuracy	F1	Precision	Recall	Specificity
	•	•	•	•		0.999 ± 0.000	0.956 ± 0.006	0.955 ± 0.006	0.954 ± 0.006	0.956 ± 0.006	0.998 ± 0.000
	•	•	•	•	•	0.999 ± 0.000	0.964 ± 0.002	0.962 ± 0.001	0.961 ± 0.001	0.964 ± 0.002	0.998 ± 0.000
•					•	1.000 ± 0.000	0.975 ± 0.002	0.974 ± 0.002	0.975 ± 0.002	0.975 ± 0.002	0.999 ± 0.000
•				•		0.999 ± 0.000	0.966 ± 0.005	0.964 ± 0.005	0.963 ± 0.004	0.966 ± 0.005	0.998 ± 0.000
•				•	•	0.999 ± 0.000	0.972 ± 0.003	0.971 ± 0.003	0.970 ± 0.003	0.972 ± 0.003	0.998 ± 0.000
•			•			0.998 ± 0.000	0.943 ± 0.005	0.940 ± 0.004	0.939 ± 0.004	0.943 ± 0.005	0.997 ± 0.000
•			•		•	0.999 ± 0.000	0.961 ± 0.006	0.958 ± 0.004	0.957 ± 0.003	0.961 ± 0.006	0.998 ± 0.000
•			•	•		0.999 ± 0.000	0.961 ± 0.005	0.960 ± 0.005	0.959 ± 0.006	0.961 ± 0.005	0.998 ± 0.000
•			•	•	•	0.999 ± 0.000	0.966 ± 0.003	0.965 ± 0.003	0.964 ± 0.003	0.966 ± 0.003	0.998 ± 0.000
•		•				0.999 ± 0.000	0.955 ± 0.004	0.952 ± 0.005	0.951 ± 0.006	0.955 ± 0.004	0.998 ± 0.000
•		•			•	1.000 ± 0.000	0.974 ± 0.003	0.972 ± 0.002	0.972 ± 0.003	0.974 ± 0.003	0.999 ± 0.000
•		•		•		0.999 ± 0.000	0.966 ± 0.006	0.965 ± 0.007	0.964 ± 0.008	0.966 ± 0.006	0.998 ± 0.000
•		•		•	•	0.999 ± 0.000	0.972 ± 0.006	0.970 ± 0.006	0.970 ± 0.005	0.972 ± 0.006	0.998 ± 0.000
•		•	•			0.998 ± 0.000	0.947 ± 0.003	0.945 ± 0.003	0.945 ± 0.003	0.947 ± 0.003	0.997 ± 0.000
•		•	•		•	0.999 ± 0.000	0.959 ± 0.003	0.957 ± 0.002	0.957 ± 0.002	0.959 ± 0.003	0.998 ± 0.000
•		•	•	•		0.999 ± 0.000	0.957 ± 0.003	0.956 ± 0.003	0.955 ± 0.003	0.957 ± 0.003	0.998 ± 0.000
•		•	•	•	•	0.999 ± 0.000	0.966 ± 0.004	0.965 ± 0.004	0.964 ± 0.004	0.966 ± 0.004	0.998 ± 0.000
•	•					0.999 ± 0.000	0.959 ± 0.006	0.957 ± 0.006	0.956 ± 0.007	0.959 ± 0.006	0.998 ± 0.000
•	•				•	1.000 ± 0.000	0.972 ± 0.001	0.972 ± 0.001	0.972 ± 0.002	0.972 ± 0.001	0.999 ± 0.000
•	•			•		0.999 ± 0.000	0.967 ± 0.003	0.966 ± 0.003	0.966 ± 0.003	0.967 ± 0.003	0.998 ± 0.000
•	•			•	•	0.999 ± 0.000	0.974 ± 0.004	0.973 ± 0.004	0.972 ± 0.004	0.974 ± 0.004	0.999 ± 0.000
•	•		•			0.999 ± 0.000	0.950 ± 0.005	0.948 ± 0.006	0.947 ± 0.007	0.950 ± 0.005	0.997 ± 0.000
•	•		•		•	0.999 ± 0.000	0.961 ± 0.001	0.958 ± 0.002	0.957 ± 0.002	0.961 ± 0.001	0.998 ± 0.000
•	•		•	•		0.999 ± 0.000	0.963 ± 0.004	0.962 ± 0.004	0.962 ± 0.004	0.963 ± 0.004	0.998 ± 0.000
•	•		•	•	•	0.999 ± 0.000	0.967 ± 0.002	0.965 ± 0.003	0.965 ± 0.004	0.967 ± 0.002	0.998 ± 0.000
•	•	•				0.999 ± 0.000	0.956 ± 0.002	0.954 ± 0.002	0.953 ± 0.002	0.956 ± 0.002	0.998 ± 0.000
•	•	•			•	0.999 ± 0.000	0.973 ± 0.004	0.972 ± 0.004	0.972 ± 0.004	0.973 ± 0.004	0.999 ± 0.000
•	•	•		•		0.999 ± 0.000	0.966 ± 0.002	0.965 ± 0.003	0.964 ± 0.003	0.966 ± 0.002	0.998 ± 0.000
•	•	•		•	•	0.999 ± 0.000	0.977 ± 0.003	0.975 ± 0.003	0.974 ± 0.003	0.977 ± 0.003	0.999 ± 0.000
•	•	•	•			0.999 ± 0.000	0.950 ± 0.004	0.947 ± 0.004	0.947 ± 0.004	0.950 ± 0.004	0.997 ± 0.000
•	•	•	•		•	0.999 ± 0.000	0.962 ± 0.003	0.960 ± 0.003	0.960 ± 0.004	0.962 ± 0.003	0.998 ± 0.000
•	•	•	•	•		0.999 ± 0.000	0.962 ± 0.005	0.960 ± 0.005	0.959 ± 0.005	0.962 ± 0.005	0.998 ± 0.000
•	•	•	•	•	•	0.999 ± 0.000	0.968 ± 0.004	0.967 ± 0.005	0.966 ± 0.005	0.968 ± 0.004	0.998 ± 0.000

Table S6: Classification metrics for AttOmics IF with different omics combination on TCGA dataset

mRNA	nc mRNA	miRNA	CNV	DNAm	Protein	AUROC	Accuracy	F1	Precision	Recall	Specificity
				•	•	1.000 ± 0.000	0.987 ± 0.002	0.986 ± 0.003	0.986 ± 0.003	0.987 ± 0.002	0.999 ± 0.000
			•		•	0.999 ± 0.000	0.959 ± 0.002	0.958 ± 0.002	0.958 ± 0.002	0.959 ± 0.002	0.998 ± 0.000
			•	•		0.999 ± 0.000	0.970 ± 0.001	0.968 ± 0.001	0.968 ± 0.001	0.970 ± 0.001	0.998 ± 0.000
			•	•	•	1.000 ± 0.000	0.981 ± 0.002	0.979 ± 0.003	0.979 ± 0.004	0.981 ± 0.002	0.999 ± 0.000
		•			•	1.000 ± 0.000	0.975 ± 0.005	0.975 ± 0.006	0.976 ± 0.006	0.975 ± 0.005	0.999 ± 0.000
		•		•		1.000 ± 0.000	0.977 ± 0.003	0.975 ± 0.003	0.975 ± 0.003	0.977 ± 0.003	0.999 ± 0.000
		•		•	•	1.000 ± 0.000	0.983 ± 0.002	0.982 ± 0.003	0.982 ± 0.003	0.983 ± 0.002	0.999 ± 0.000
		•	•			0.997 ± 0.000	0.925 ± 0.004	0.922 ± 0.006	0.921 ± 0.008	0.925 ± 0.004	0.996 ± 0.000
		•	•		•	0.999 ± 0.000	0.971 ± 0.004	0.969 ± 0.005	0.969 ± 0.007	0.971 ± 0.004	0.998 ± 0.000
		•	•	•		0.999 ± 0.000	0.973 ± 0.005	0.972 ± 0.005	0.973 ± 0.006	0.973 ± 0.005	0.999 ± 0.000
		•	•	•	•	1.000 ± 0.000	0.981 ± 0.002	0.980 ± 0.002	0.980 ± 0.002	0.981 ± 0.002	0.999 ± 0.000
	•				•	1.000 ± 0.000	0.984 ± 0.003	0.983 ± 0.002	0.983 ± 0.002	0.984 ± 0.003	0.999 ± 0.000
	•			•		1.000 ± 0.000	0.984 ± 0.003	0.982 ± 0.003	0.982 ± 0.004	0.984 ± 0.003	0.999 ± 0.000
	•			•	•	1.000 ± 0.000	0.987 ± 0.001	0.986 ± 0.001	0.986 ± 0.001	0.987 ± 0.001	0.999 ± 0.000
	•		•			0.999 ± 0.000	0.973 ± 0.001	0.972 ± 0.001	0.971 ± 0.001	0.973 ± 0.001	0.999 ± 0.000
	•		•		•	1.000 ± 0.000	0.983 ± 0.002	0.981 ± 0.002	0.981 ± 0.002	0.983 ± 0.002	0.999 ± 0.000
	•		•	•		1.000 ± 0.000	0.981 ± 0.005	0.980 ± 0.005	0.979 ± 0.004	0.981 ± 0.005	0.999 ± 0.000
	•		•	•	•	1.000 ± 0.000	0.988 ± 0.003	0.987 ± 0.004	0.986 ± 0.004	0.988 ± 0.003	0.999 ± 0.000
	•	•				1.000 ± 0.000	0.976 ± 0.006	0.974 ± 0.007	0.974 ± 0.007	0.976 ± 0.006	0.999 ± 0.000
	•	•			•	1.000 ± 0.000	0.983 ± 0.005	0.982 ± 0.005	0.982 ± 0.005	0.983 ± 0.005	0.999 ± 0.000
	•	•		•		1.000 ± 0.000	0.985 ± 0.001	0.983 ± 0.001	0.983 ± 0.001	0.985 ± 0.001	0.999 ± 0.000
	•	•		•	•	1.000 ± 0.000	0.988 ± 0.001	0.987 ± 0.001	0.986 ± 0.001	0.988 ± 0.001	0.999 ± 0.000
	•	•	•			1.000 ± 0.000	0.978 ± 0.002	0.975 ± 0.003	0.975 ± 0.004	0.978 ± 0.002	0.999 ± 0.000
	•	•	•		•	1.000 ± 0.000	0.984 ± 0.003	0.984 ± 0.003	0.984 ± 0.003	0.984 ± 0.003	0.999 ± 0.000
	•	•	•	•		1.000 ± 0.000	0.983 ± 0.003	0.982 ± 0.003	0.981 ± 0.002	0.983 ± 0.003	0.999 ± 0.000
	•	•	•	•	•	1.000 ± 0.000	0.987 ± 0.004	0.985 ± 0.004	0.984 ± 0.004	0.987 ± 0.004	0.999 ± 0.000
•					•	1.000 ± 0.000	0.981 ± 0.002	0.980 ± 0.002	0.980 ± 0.002	0.981 ± 0.002	0.999 ± 0.000
•				•		1.000 ± 0.000	0.982 ± 0.001	0.981 ± 0.001	0.981 ± 0.001	0.982 ± 0.001	0.999 ± 0.000
•				•	•	1.000 ± 0.000	0.986 ± 0.002	0.984 ± 0.002	0.983 ± 0.003	0.986 ± 0.002	0.999 ± 0.000
•			•			0.999 ± 0.000	0.967 ± 0.004	0.964 ± 0.003	0.963 ± 0.002	0.967 ± 0.004	0.998 ± 0.000
•			•		•	1.000 ± 0.000	0.978 ± 0.004	0.977 ± 0.004	0.977 ± 0.004	0.978 ± 0.004	0.999 ± 0.000
•			•	•		1.000 ± 0.000	0.978 ± 0.001	0.976 ± 0.001	0.975 ± 0.002	0.978 ± 0.001	0.999 ± 0.000
•			•	•	•	1.000 ± 0.000	0.984 ± 0.002	0.983 ± 0.002	0.983 ± 0.002	0.984 ± 0.002	0.999 ± 0.000
•		•				0.999 ± 0.000	0.973 ± 0.003	0.972 ± 0.003	0.971 ± 0.003	0.973 ± 0.003	0.999 ± 0.000
•		•			•	1.000 ± 0.000	0.983 ± 0.001	0.982 ± 0.001	0.981 ± 0.001	0.983 ± 0.001	0.999 ± 0.000
•		•		•		1.000 ± 0.000	0.983 ± 0.002	0.981 ± 0.003	0.980 ± 0.003	0.983 ± 0.002	0.999 ± 0.000
•		•		•	•	1.000 ± 0.000	0.985 ± 0.003	0.984 ± 0.003	0.983 ± 0.003	0.985 ± 0.003	0.999 ± 0.000
•		•	•			0.999 ± 0.000	0.971 ± 0.003	0.969 ± 0.004	0.968 ± 0.004	0.971 ± 0.003	0.998 ± 0.000
•		•	•		•	1.000 ± 0.000	0.981 ± 0.002	0.980 ± 0.002	0.980 ± 0.002	0.981 ± 0.002	0.999 ± 0.000
•		•	•	•		1.000 ± 0.000	0.978 ± 0.001	0.977 ± 0.002	0.977 ± 0.002	0.978 ± 0.001	0.999 ± 0.000

Continued on next page

Table S6: Classification metrics for AttOmics IF with different omics combination

mRNA	nc mRNA	miRNA	CNV	DNAm	Protein	AUROC	Accuracy	F1	Precision	Recall	Specificity
•		•	•	•	•	1.000 ± 0.000	0.987 ± 0.002	0.986 ± 0.001	0.985 ± 0.001	0.987 ± 0.002	0.999 ± 0.000
•	•					1.000 ± 0.000	0.976 ± 0.003	0.974 ± 0.004	0.974 ± 0.004	0.976 ± 0.003	0.999 ± 0.000
•	•				•	1.000 ± 0.000	0.984 ± 0.002	0.983 ± 0.003	0.982 ± 0.003	0.984 ± 0.002	0.999 ± 0.000
•	•			•		1.000 ± 0.000	0.984 ± 0.004	0.982 ± 0.004	0.981 ± 0.004	0.984 ± 0.004	0.999 ± 0.000
•	•			•	•	1.000 ± 0.000	0.987 ± 0.002	0.986 ± 0.002	0.985 ± 0.002	0.987 ± 0.002	0.999 ± 0.000
•	•		•			0.999 ± 0.000	0.979 ± 0.003	0.976 ± 0.003	0.975 ± 0.004	0.979 ± 0.003	0.999 ± 0.000
•	•		•		•	1.000 ± 0.000	0.979 ± 0.002	0.978 ± 0.002	0.978 ± 0.001	0.979 ± 0.002	0.999 ± 0.000
•	•		•	•		1.000 ± 0.000	0.981 ± 0.003	0.979 ± 0.003	0.979 ± 0.003	0.981 ± 0.003	0.999 ± 0.000
•	•		•	•	•	1.000 ± 0.000	0.983 ± 0.002	0.982 ± 0.001	0.982 ± 0.002	0.983 ± 0.002	0.999 ± 0.000
•	•	•				1.000 ± 0.000	0.980 ± 0.003	0.978 ± 0.003	0.977 ± 0.003	0.980 ± 0.003	0.999 ± 0.000
•	•	•			•	1.000 ± 0.000	0.985 ± 0.001	0.984 ± 0.001	0.983 ± 0.002	0.985 ± 0.001	0.999 ± 0.000
•	•	•		•		1.000 ± 0.000	0.982 ± 0.002	0.981 ± 0.002	0.980 ± 0.001	0.982 ± 0.002	0.999 ± 0.000
•	•	•		•	•	1.000 ± 0.000	0.988 ± 0.001	0.986 ± 0.001	0.985 ± 0.001	0.988 ± 0.001	0.999 ± 0.000
•	•	•	•			1.000 ± 0.000	0.979 ± 0.002	0.978 ± 0.002	0.978 ± 0.002	0.979 ± 0.002	0.999 ± 0.000
•	•	•	•		•	1.000 ± 0.000	0.984 ± 0.003	0.984 ± 0.003	0.984 ± 0.003	0.984 ± 0.003	0.999 ± 0.000
•	•	•	•	•		1.000 ± 0.000	0.982 ± 0.001	0.981 ± 0.002	0.980 ± 0.003	0.982 ± 0.001	0.999 ± 0.000
•	•	•	•	•	•	1.000 ± 0.000	0.985 ± 0.002	0.983 ± 0.002	0.983 ± 0.002	0.985 ± 0.002	0.999 ± 0.000

Table S7: Classification metrics for CrossAttOmics with different omics combination on TCGA dataset

mRNA	nc mRNA	miRNA	CNV	DNAm	Protein	AUROC	Accuracy	F1	Precision	Recall	Specificity
		•		•		1.000 ± 0.000	0.976 ± 0.003	0.975 ± 0.003	0.975 ± 0.003	0.976 ± 0.003	0.999 ± 0.000
		•		•	•	1.000 ± 0.000	0.984 ± 0.001	0.983 ± 0.001	0.983 ± 0.001	0.984 ± 0.001	0.999 ± 0.000
		•	•			0.997 ± 0.000	0.928 ± 0.006	0.926 ± 0.006	0.926 ± 0.006	0.928 ± 0.006	0.996 ± 0.000
		•	•		•	1.000 ± 0.000	0.969 ± 0.004	0.968 ± 0.004	0.968 ± 0.004	0.969 ± 0.004	0.998 ± 0.000
		•	•	•		0.999 ± 0.000	0.971 ± 0.005	0.969 ± 0.005	0.970 ± 0.007	0.971 ± 0.005	0.998 ± 0.000
		•	•	•	•	1.000 ± 0.000	0.979 ± 0.002	0.979 ± 0.002	0.979 ± 0.002	0.979 ± 0.002	0.999 ± 0.000
	•			•		1.000 ± 0.000	0.986 ± 0.003	0.984 ± 0.003	0.983 ± 0.003	0.986 ± 0.003	0.999 ± 0.000
	•			•	•	1.000 ± 0.000	0.987 ± 0.001	0.985 ± 0.001	0.985 ± 0.001	0.987 ± 0.001	0.999 ± 0.000
	•		•			0.999 ± 0.000	0.971 ± 0.004	0.970 ± 0.004	0.969 ± 0.004	0.971 ± 0.004	0.998 ± 0.000
	•		•		•	1.000 ± 0.000	0.985 ± 0.001	0.984 ± 0.001	0.984 ± 0.001	0.985 ± 0.001	0.999 ± 0.000
	•		•	•		1.000 ± 0.000	0.984 ± 0.003	0.983 ± 0.003	0.982 ± 0.003	0.984 ± 0.003	0.999 ± 0.000
	•		•	•	•	1.000 ± 0.000	0.986 ± 0.001	0.985 ± 0.001	0.984 ± 0.001	0.986 ± 0.001	0.999 ± 0.000
	•	•		•		1.000 ± 0.000	0.987 ± 0.003	0.985 ± 0.003	0.985 ± 0.002	0.987 ± 0.003	0.999 ± 0.000
	•	•		•	•	1.000 ± 0.000	0.987 ± 0.004	0.986 ± 0.004	0.985 ± 0.004	0.987 ± 0.004	0.999 ± 0.000
	•	•	•			1.000 ± 0.000	0.977 ± 0.005	0.974 ± 0.005	0.974 ± 0.005	0.977 ± 0.005	0.999 ± 0.000
	•	•	•		•	1.000 ± 0.000	0.984 ± 0.002	0.984 ± 0.002	0.984 ± 0.001	0.984 ± 0.002	0.999 ± 0.000
	•	•	•	•		1.000 ± 0.000	0.984 ± 0.003	0.982 ± 0.003	0.982 ± 0.003	0.984 ± 0.003	0.999 ± 0.000

Continued on next page

Table S7: Classification metrics for CrossAttOmics with different omics combination

mRNA	nc mRNA	miRNA	CNV	DNAm	Protein	AUROC	Accuracy	F1	Precision	Recall	Specificity
	•	•	•	•	•	1.000 ± 0.000	0.985 ± 0.004	0.983 ± 0.004	0.983 ± 0.005	0.985 ± 0.004	0.999 ± 0.000
•					•	1.000 ± 0.000	0.981 ± 0.004	0.980 ± 0.004	0.979 ± 0.004	0.981 ± 0.004	0.999 ± 0.000
•				•		1.000 ± 0.000	0.982 ± 0.002	0.980 ± 0.002	0.979 ± 0.002	0.982 ± 0.002	0.999 ± 0.000
•				•	•	1.000 ± 0.000	0.986 ± 0.001	0.984 ± 0.001	0.983 ± 0.002	0.986 ± 0.001	0.999 ± 0.000
•			•			0.999 ± 0.000	0.965 ± 0.002	0.963 ± 0.002	0.963 ± 0.002	0.965 ± 0.002	0.998 ± 0.000
•			•		•	1.000 ± 0.000	0.977 ± 0.005	0.975 ± 0.004	0.975 ± 0.003	0.977 ± 0.005	0.999 ± 0.000
•			•	•		1.000 ± 0.000	0.978 ± 0.001	0.976 ± 0.001	0.976 ± 0.001	0.978 ± 0.001	0.999 ± 0.000
•			•	•	•	1.000 ± 0.000	0.981 ± 0.002	0.980 ± 0.002	0.979 ± 0.001	0.981 ± 0.002	0.999 ± 0.000
•		•				0.999 ± 0.000	0.972 ± 0.003	0.971 ± 0.003	0.971 ± 0.004	0.972 ± 0.003	0.999 ± 0.000
•		•			•	1.000 ± 0.000	0.984 ± 0.002	0.982 ± 0.002	0.982 ± 0.001	0.984 ± 0.002	0.999 ± 0.000
•		•		•		1.000 ± 0.000	0.980 ± 0.001	0.978 ± 0.001	0.978 ± 0.001	0.980 ± 0.001	0.999 ± 0.000
•		•		•	•	1.000 ± 0.000	0.984 ± 0.002	0.983 ± 0.002	0.983 ± 0.002	0.984 ± 0.002	0.999 ± 0.000
•		•	•			1.000 ± 0.000	0.972 ± 0.004	0.971 ± 0.004	0.970 ± 0.004	0.972 ± 0.004	0.999 ± 0.000
•		•	•		•	1.000 ± 0.000	0.979 ± 0.005	0.978 ± 0.005	0.978 ± 0.005	0.979 ± 0.005	0.999 ± 0.000
•		•	•	•		1.000 ± 0.000	0.978 ± 0.003	0.977 ± 0.002	0.977 ± 0.002	0.978 ± 0.003	0.999 ± 0.000
•		•	•	•	•	1.000 ± 0.000	0.983 ± 0.002	0.982 ± 0.002	0.982 ± 0.002	0.983 ± 0.002	0.999 ± 0.000
•	•					1.000 ± 0.000	0.976 ± 0.002	0.974 ± 0.002	0.973 ± 0.002	0.976 ± 0.002	0.999 ± 0.000
•	•				•	1.000 ± 0.000	0.982 ± 0.003	0.980 ± 0.003	0.980 ± 0.003	0.982 ± 0.003	0.999 ± 0.000
•	•			•		1.000 ± 0.000	0.983 ± 0.002	0.982 ± 0.002	0.981 ± 0.002	0.983 ± 0.002	0.999 ± 0.000
•	•			•	•	1.000 ± 0.000	0.985 ± 0.002	0.984 ± 0.001	0.983 ± 0.001	0.985 ± 0.002	0.999 ± 0.000
•	•		•			1.000 ± 0.000	0.975 ± 0.003	0.974 ± 0.003	0.974 ± 0.004	0.975 ± 0.003	0.999 ± 0.000
•	•		•		•	1.000 ± 0.000	0.979 ± 0.003	0.977 ± 0.003	0.977 ± 0.004	0.979 ± 0.003	0.999 ± 0.000
•	•		•	•		1.000 ± 0.000	0.980 ± 0.002	0.979 ± 0.002	0.978 ± 0.003	0.980 ± 0.002	0.999 ± 0.000
•	•		•	•	•	1.000 ± 0.000	0.983 ± 0.004	0.982 ± 0.004	0.981 ± 0.004	0.983 ± 0.004	0.999 ± 0.000
•	•	•				1.000 ± 0.000	0.976 ± 0.003	0.974 ± 0.003	0.974 ± 0.003	0.976 ± 0.003	0.999 ± 0.000
•	•	•			•	1.000 ± 0.000	0.981 ± 0.003	0.980 ± 0.003	0.980 ± 0.003	0.981 ± 0.003	0.999 ± 0.000
•	•	•		•		1.000 ± 0.000	0.982 ± 0.002	0.980 ± 0.003	0.979 ± 0.003	0.982 ± 0.002	0.999 ± 0.000
•	•	•		•	•	1.000 ± 0.000	0.983 ± 0.004	0.981 ± 0.004	0.981 ± 0.004	0.983 ± 0.004	0.999 ± 0.000
•	•	•	•			1.000 ± 0.000	0.976 ± 0.003	0.975 ± 0.003	0.975 ± 0.003	0.976 ± 0.003	0.999 ± 0.000
•	•	•	•		•	1.000 ± 0.000	0.978 ± 0.002	0.977 ± 0.001	0.977 ± 0.001	0.978 ± 0.002	0.999 ± 0.000
•	•	•	•	•		1.000 ± 0.000	0.981 ± 0.002	0.980 ± 0.002	0.979 ± 0.002	0.981 ± 0.002	0.999 ± 0.000
•	•	•	•	•	•	1.000 ± 0.000	0.981 ± 0.001	0.979 ± 0.002	0.979 ± 0.002	0.981 ± 0.001	0.999 ± 0.000

Table S8: Classification metrics for GNN with different omics combination on TCGA dataset

mRNA	nc mRNA	miRNA	CNV	DNAm	Protein	AUROC	Accuracy	F1	Precision	Recall	Specificity
				•		0.998 ± 0.000	0.954 ± 0.003	0.954 ± 0.002	0.956 ± 0.002	0.954 ± 0.003	0.998 ± 0.000
			•			0.946 ± 0.007	0.623 ± 0.017	0.615 ± 0.027	0.655 ± 0.022	0.623 ± 0.017	0.977 ± 0.001

Continued on next page

Table S8: Classification metrics for GNN with different omics combination

mRNA	nc mRNA	miRNA	CNV	DNAm	Protein	AUROC	Accuracy	F1	Precision	Recall	Specificity
•						0.998 ± 0.000	0.956 ± 0.005	0.955 ± 0.004	0.956 ± 0.005	0.956 ± 0.005	0.998 ± 0.000

Table S9: Classification metrics for GNN EF with different omics combination on TCGA dataset

mRNA	nc mRNA	miRNA	CNV	DNAm	Protein	AUROC	Accuracy	F1	Precision	Recall	Specificity
			•	•		0.998 ± 0.000	0.952 ± 0.006	0.952 ± 0.005	0.953 ± 0.005	0.952 ± 0.006	0.997 ± 0.000
•				•		0.999 ± 0.000	0.959 ± 0.007	0.958 ± 0.006	0.958 ± 0.006	0.959 ± 0.007	0.998 ± 0.000
•			•			0.999 ± 0.000	0.963 ± 0.005	0.960 ± 0.005	0.959 ± 0.005	0.963 ± 0.005	0.998 ± 0.000
•			•	•		0.999 ± 0.001	0.961 ± 0.001	0.960 ± 0.002	0.961 ± 0.002	0.961 ± 0.001	0.998 ± 0.000

Table S10: Classification metrics for MLP with different omics combination on TCGA dataset

mRNA	nc mRNA	miRNA	CNV	DNAm	Protein	AUROC	Accuracy	F1	Precision	Recall	Specificity
					•	1.000 ± 0.000	0.990 ± 0.002	0.985 ± 0.002	0.981 ± 0.002	0.990 ± 0.002	0.999 ± 0.000
				•		0.999 ± 0.000	0.965 ± 0.001	0.965 ± 0.001	0.967 ± 0.001	0.965 ± 0.001	0.998 ± 0.000
			•			0.969 ± 0.001	0.733 ± 0.009	0.734 ± 0.010	0.749 ± 0.011	0.733 ± 0.009	0.985 ± 0.001
		•				0.997 ± 0.000	0.926 ± 0.004	0.926 ± 0.004	0.928 ± 0.004	0.926 ± 0.004	0.996 ± 0.000
	•					0.998 ± 0.001	0.953 ± 0.002	0.951 ± 0.002	0.951 ± 0.002	0.953 ± 0.002	0.998 ± 0.000
•						0.998 ± 0.000	0.967 ± 0.002	0.967 ± 0.002	0.967 ± 0.002	0.967 ± 0.002	0.998 ± 0.000

Table S11: Classification metrics for MLP EF with different omics combination on TCGA dataset

mRNA	nc mRNA	miRNA	CNV	DNAm	Protein	AUROC	Accuracy	F1	Precision	Recall	Specificity
				•	•	1.000 ± 0.000	0.983 ± 0.002	0.981 ± 0.001	0.982 ± 0.001	0.983 ± 0.002	0.999 ± 0.000
			•		•	0.999 ± 0.000	0.965 ± 0.004	0.962 ± 0.003	0.961 ± 0.003	0.965 ± 0.004	0.998 ± 0.000
			•	•		0.999 ± 0.000	0.959 ± 0.002	0.958 ± 0.002	0.957 ± 0.001	0.959 ± 0.002	0.998 ± 0.000
			•	•	•	0.999 ± 0.000	0.971 ± 0.004	0.969 ± 0.005	0.968 ± 0.005	0.971 ± 0.004	0.998 ± 0.000
		•			•	1.000 ± 0.000	0.990 ± 0.002	0.990 ± 0.002	0.990 ± 0.003	0.990 ± 0.002	0.999 ± 0.000
		•		•		0.999 ± 0.000	0.975 ± 0.002	0.974 ± 0.002	0.974 ± 0.002	0.975 ± 0.002	0.999 ± 0.000
		•		•	•	1.000 ± 0.000	0.983 ± 0.003	0.982 ± 0.003	0.982 ± 0.003	0.983 ± 0.003	0.999 ± 0.000
		•	•			0.993 ± 0.001	0.877 ± 0.013	0.877 ± 0.013	0.881 ± 0.011	0.877 ± 0.013	0.993 ± 0.001
		•	•		•	1.000 ± 0.000	0.968 ± 0.002	0.967 ± 0.003	0.966 ± 0.004	0.968 ± 0.002	0.998 ± 0.000
		•	•	•		0.998 ± 0.001	0.963 ± 0.003	0.962 ± 0.004	0.962 ± 0.005	0.963 ± 0.003	0.998 ± 0.000

Continued on next page

Table S11: Classification metrics for MLP EF with different omics combination

mRNA	nc mRNA	miRNA	CNV	DNAm	Protein	AUROC	Accuracy	F1	Precision	Recall	Specificity
		•	•	•	•	0.999 ± 0.000	0.975 ± 0.002	0.974 ± 0.003	0.973 ± 0.003	0.975 ± 0.002	0.999 ± 0.000
	•				•	0.998 ± 0.001	0.979 ± 0.004	0.979 ± 0.003	0.980 ± 0.003	0.979 ± 0.004	0.999 ± 0.000
	•			•		0.999 ± 0.001	0.973 ± 0.002	0.971 ± 0.002	0.972 ± 0.001	0.973 ± 0.002	0.999 ± 0.000
	•			•	•	0.999 ± 0.000	0.977 ± 0.003	0.975 ± 0.003	0.975 ± 0.004	0.977 ± 0.003	0.999 ± 0.000
	•		•			0.996 ± 0.001	0.942 ± 0.006	0.938 ± 0.006	0.936 ± 0.007	0.942 ± 0.006	0.997 ± 0.000
	•		•		•	0.998 ± 0.000	0.967 ± 0.003	0.965 ± 0.004	0.964 ± 0.005	0.967 ± 0.003	0.998 ± 0.000
	•		•	•		0.998 ± 0.000	0.968 ± 0.004	0.965 ± 0.004	0.964 ± 0.005	0.968 ± 0.004	0.998 ± 0.000
	•		•	•	•	0.999 ± 0.000	0.975 ± 0.004	0.972 ± 0.004	0.971 ± 0.003	0.975 ± 0.004	0.999 ± 0.000
	•	•				0.996 ± 0.001	0.961 ± 0.001	0.960 ± 0.002	0.961 ± 0.003	0.961 ± 0.001	0.998 ± 0.000
	•	•			•	0.998 ± 0.001	0.979 ± 0.002	0.979 ± 0.002	0.980 ± 0.002	0.979 ± 0.002	0.999 ± 0.000
	•	•		•		0.999 ± 0.001	0.972 ± 0.001	0.971 ± 0.001	0.972 ± 0.001	0.972 ± 0.001	0.998 ± 0.000
	•	•		•	•	0.999 ± 0.000	0.979 ± 0.002	0.977 ± 0.002	0.977 ± 0.002	0.979 ± 0.002	0.999 ± 0.000
	•	•	•			0.996 ± 0.001	0.943 ± 0.002	0.942 ± 0.003	0.941 ± 0.003	0.943 ± 0.002	0.997 ± 0.000
	•	•	•		•	0.998 ± 0.000	0.968 ± 0.004	0.966 ± 0.005	0.967 ± 0.006	0.968 ± 0.004	0.998 ± 0.000
	•	•	•	•		0.998 ± 0.001	0.968 ± 0.003	0.965 ± 0.002	0.965 ± 0.002	0.968 ± 0.003	0.998 ± 0.000
	•	•	•	•	•	0.999 ± 0.000	0.974 ± 0.002	0.972 ± 0.002	0.971 ± 0.002	0.974 ± 0.002	0.999 ± 0.000
•					•	1.000 ± 0.000	0.984 ± 0.003	0.983 ± 0.003	0.982 ± 0.003	0.984 ± 0.003	0.999 ± 0.000
•				•		0.999 ± 0.000	0.980 ± 0.002	0.978 ± 0.002	0.977 ± 0.002	0.980 ± 0.002	0.999 ± 0.000
•				•	•	1.000 ± 0.000	0.982 ± 0.002	0.981 ± 0.002	0.981 ± 0.002	0.982 ± 0.002	0.999 ± 0.000
•			•			0.998 ± 0.000	0.955 ± 0.001	0.952 ± 0.002	0.951 ± 0.002	0.955 ± 0.001	0.998 ± 0.000
•			•		•	0.999 ± 0.000	0.973 ± 0.001	0.971 ± 0.001	0.970 ± 0.002	0.973 ± 0.001	0.999 ± 0.000
•			•	•		0.999 ± 0.000	0.971 ± 0.005	0.969 ± 0.005	0.968 ± 0.005	0.971 ± 0.005	0.998 ± 0.000
•			•	•	•	0.999 ± 0.000	0.980 ± 0.002	0.978 ± 0.003	0.977 ± 0.003	0.980 ± 0.002	0.999 ± 0.000
•		•				0.999 ± 0.000	0.969 ± 0.003	0.967 ± 0.003	0.967 ± 0.003	0.969 ± 0.003	0.998 ± 0.000
•		•			•	1.000 ± 0.000	0.985 ± 0.002	0.984 ± 0.002	0.984 ± 0.002	0.985 ± 0.002	0.999 ± 0.000
•		•		•		0.999 ± 0.000	0.979 ± 0.002	0.978 ± 0.002	0.977 ± 0.003	0.979 ± 0.002	0.999 ± 0.000
•		•		•	•	1.000 ± 0.000	0.983 ± 0.002	0.982 ± 0.002	0.982 ± 0.002	0.983 ± 0.002	0.999 ± 0.000
•		•	•			0.998 ± 0.000	0.955 ± 0.004	0.951 ± 0.005	0.950 ± 0.005	0.955 ± 0.004	0.998 ± 0.000
•		•	•		•	0.999 ± 0.000	0.969 ± 0.002	0.967 ± 0.004	0.966 ± 0.005	0.969 ± 0.002	0.998 ± 0.000
•		•	•	•		0.999 ± 0.000	0.969 ± 0.004	0.967 ± 0.004	0.967 ± 0.004	0.969 ± 0.004	0.998 ± 0.000
•		•	•	•	•	0.999 ± 0.000	0.978 ± 0.002	0.976 ± 0.003	0.975 ± 0.003	0.978 ± 0.002	0.999 ± 0.000
•	•					0.998 ± 0.001	0.963 ± 0.004	0.961 ± 0.004	0.962 ± 0.004	0.963 ± 0.004	0.998 ± 0.000
•	•				•	0.999 ± 0.000	0.978 ± 0.004	0.977 ± 0.004	0.978 ± 0.004	0.978 ± 0.004	0.999 ± 0.000
•	•			•		0.999 ± 0.000	0.974 ± 0.003	0.971 ± 0.003	0.971 ± 0.003	0.974 ± 0.003	0.999 ± 0.000
•	•			•	•	0.999 ± 0.000	0.976 ± 0.002	0.974 ± 0.003	0.973 ± 0.004	0.976 ± 0.002	0.999 ± 0.000
•	•		•			0.998 ± 0.000	0.955 ± 0.002	0.952 ± 0.002	0.952 ± 0.002	0.955 ± 0.002	0.998 ± 0.000
•	•		•		•	0.999 ± 0.001	0.966 ± 0.002	0.964 ± 0.003	0.964 ± 0.003	0.966 ± 0.002	0.998 ± 0.000
•	•		•	•		0.999 ± 0.000	0.972 ± 0.002	0.969 ± 0.002	0.969 ± 0.002	0.972 ± 0.002	0.998 ± 0.000
•	•		•	•	•	0.999 ± 0.000	0.974 ± 0.003	0.971 ± 0.003	0.971 ± 0.003	0.974 ± 0.003	0.999 ± 0.000
•	•	•				0.998 ± 0.000	0.964 ± 0.002	0.962 ± 0.001	0.963 ± 0.002	0.964 ± 0.002	0.998 ± 0.000

Continued on next page

Table S11: Classification metrics for MLP EF with different omics combination

mRNA	nc mRNA	miRNA	CNV	DNAm	Protein	AUROC	Accuracy	F1	Precision	Recall	Specificity
•	•	•			•	0.999 ± 0.001	0.979 ± 0.002	0.978 ± 0.003	0.978 ± 0.003	0.979 ± 0.002	0.999 ± 0.000
•	•	•		•		0.999 ± 0.000	0.974 ± 0.004	0.972 ± 0.003	0.972 ± 0.003	0.974 ± 0.004	0.999 ± 0.000
•	•	•		•	•	0.999 ± 0.000	0.976 ± 0.002	0.975 ± 0.002	0.975 ± 0.002	0.976 ± 0.002	0.999 ± 0.000
•	•	•	•			0.998 ± 0.000	0.955 ± 0.006	0.952 ± 0.007	0.951 ± 0.007	0.955 ± 0.006	0.998 ± 0.000
•	•	•	•		•	0.999 ± 0.000	0.965 ± 0.003	0.964 ± 0.003	0.964 ± 0.003	0.965 ± 0.003	0.998 ± 0.000
•	•	•	•	•		0.999 ± 0.000	0.974 ± 0.003	0.971 ± 0.003	0.970 ± 0.002	0.974 ± 0.003	0.999 ± 0.000
•	•	•	•	•	•	0.999 ± 0.000	0.975 ± 0.003	0.972 ± 0.003	0.971 ± 0.003	0.975 ± 0.003	0.999 ± 0.000

Table S12: Classification metrics for MLP IF with different omics combination on TCGA dataset

mRNA	nc mRNA	miRNA	CNV	DNAm	Protein	AUROC	Accuracy	F1	Precision	Recall	Specificity
				•	•	0.998 ± 0.001	0.980 ± 0.002	0.978 ± 0.002	0.978 ± 0.003	0.980 ± 0.002	0.999 ± 0.000
			•		•	0.999 ± 0.000	0.973 ± 0.002	0.972 ± 0.002	0.972 ± 0.003	0.973 ± 0.002	0.998 ± 0.000
			•	•		0.998 ± 0.001	0.974 ± 0.002	0.973 ± 0.002	0.973 ± 0.002	0.974 ± 0.002	0.999 ± 0.000
			•	•	•	0.999 ± 0.000	0.983 ± 0.003	0.982 ± 0.003	0.982 ± 0.003	0.983 ± 0.003	0.999 ± 0.000
		•			•	0.999 ± 0.000	0.979 ± 0.002	0.979 ± 0.002	0.979 ± 0.002	0.979 ± 0.002	0.999 ± 0.000
		•		•		0.998 ± 0.000	0.968 ± 0.002	0.967 ± 0.002	0.968 ± 0.003	0.968 ± 0.002	0.998 ± 0.000
		•		•	•	0.998 ± 0.001	0.977 ± 0.003	0.976 ± 0.003	0.977 ± 0.004	0.977 ± 0.003	0.999 ± 0.000
		•	•			0.997 ± 0.000	0.931 ± 0.003	0.928 ± 0.003	0.928 ± 0.004	0.931 ± 0.003	0.996 ± 0.000
		•	•		•	0.999 ± 0.000	0.975 ± 0.004	0.975 ± 0.004	0.976 ± 0.004	0.975 ± 0.004	0.999 ± 0.000
		•	•	•		0.998 ± 0.000	0.972 ± 0.002	0.970 ± 0.002	0.972 ± 0.002	0.972 ± 0.002	0.999 ± 0.000
		•	•	•	•	0.999 ± 0.001	0.979 ± 0.002	0.977 ± 0.002	0.978 ± 0.002	0.979 ± 0.002	0.999 ± 0.000
	•				•	0.998 ± 0.000	0.966 ± 0.005	0.964 ± 0.005	0.964 ± 0.004	0.966 ± 0.005	0.998 ± 0.000
	•			•		0.998 ± 0.000	0.971 ± 0.001	0.968 ± 0.002	0.968 ± 0.002	0.971 ± 0.001	0.998 ± 0.000
	•			•	•	0.998 ± 0.001	0.976 ± 0.001	0.974 ± 0.002	0.973 ± 0.002	0.976 ± 0.001	0.999 ± 0.000
	•		•			0.996 ± 0.001	0.956 ± 0.003	0.953 ± 0.003	0.952 ± 0.003	0.956 ± 0.003	0.998 ± 0.000
	•		•		•	0.998 ± 0.001	0.973 ± 0.004	0.972 ± 0.005	0.973 ± 0.005	0.973 ± 0.004	0.999 ± 0.000
	•		•	•		0.998 ± 0.000	0.972 ± 0.003	0.969 ± 0.003	0.970 ± 0.003	0.972 ± 0.003	0.998 ± 0.000
	•		•	•	•	0.999 ± 0.000	0.977 ± 0.002	0.975 ± 0.002	0.975 ± 0.002	0.977 ± 0.002	0.999 ± 0.000
	•	•				0.997 ± 0.001	0.957 ± 0.003	0.957 ± 0.004	0.959 ± 0.003	0.957 ± 0.003	0.998 ± 0.000
	•	•			•	0.998 ± 0.001	0.972 ± 0.004	0.971 ± 0.003	0.972 ± 0.003	0.972 ± 0.004	0.999 ± 0.000
	•	•		•		0.998 ± 0.001	0.971 ± 0.003	0.969 ± 0.003	0.969 ± 0.003	0.971 ± 0.003	0.998 ± 0.000
	•	•		•	•	0.998 ± 0.000	0.975 ± 0.003	0.973 ± 0.002	0.973 ± 0.002	0.975 ± 0.003	0.999 ± 0.000
	•	•	•			0.997 ± 0.001	0.963 ± 0.004	0.961 ± 0.004	0.961 ± 0.003	0.963 ± 0.004	0.998 ± 0.000
	•	•	•		•	0.998 ± 0.000	0.974 ± 0.003	0.973 ± 0.002	0.974 ± 0.002	0.974 ± 0.003	0.999 ± 0.000
	•	•	•	•		0.998 ± 0.001	0.971 ± 0.003	0.969 ± 0.002	0.969 ± 0.002	0.971 ± 0.003	0.998 ± 0.000
	•	•	•	•	•	0.999 ± 0.000	0.976 ± 0.002	0.974 ± 0.001	0.974 ± 0.001	0.976 ± 0.002	0.999 ± 0.000
•					•	0.999 ± 0.000	0.976 ± 0.003	0.974 ± 0.003	0.974 ± 0.003	0.976 ± 0.003	0.999 ± 0.000

Continued on next page

Table S12: Classification metrics for MLP IF with different omics combination

mRNA	nc mRNA	miRNA	CNV	DNAm	Protein	AUROC	Accuracy	F1	Precision	Recall	Specificity
●				●		0.998 ± 0.001	0.980 ± 0.003	0.977 ± 0.003	0.977 ± 0.003	0.980 ± 0.003	0.999 ± 0.000
●				●	●	0.999 ± 0.001	0.982 ± 0.001	0.981 ± 0.001	0.980 ± 0.002	0.982 ± 0.001	0.999 ± 0.000
●			●			0.998 ± 0.001	0.970 ± 0.002	0.967 ± 0.002	0.967 ± 0.002	0.970 ± 0.002	0.998 ± 0.000
●			●		●	0.999 ± 0.000	0.979 ± 0.003	0.977 ± 0.003	0.977 ± 0.003	0.979 ± 0.003	0.999 ± 0.000
●			●	●		0.999 ± 0.001	0.978 ± 0.002	0.976 ± 0.002	0.976 ± 0.001	0.978 ± 0.002	0.999 ± 0.000
●			●	●	●	0.999 ± 0.000	0.982 ± 0.002	0.980 ± 0.002	0.980 ± 0.002	0.982 ± 0.002	0.999 ± 0.000
●		●				0.998 ± 0.000	0.967 ± 0.002	0.965 ± 0.003	0.964 ± 0.004	0.967 ± 0.002	0.998 ± 0.000
●		●			●	0.999 ± 0.000	0.973 ± 0.002	0.971 ± 0.003	0.970 ± 0.003	0.973 ± 0.002	0.999 ± 0.000
●		●		●		0.998 ± 0.001	0.979 ± 0.002	0.977 ± 0.001	0.977 ± 0.001	0.979 ± 0.002	0.999 ± 0.000
●		●		●	●	0.999 ± 0.000	0.980 ± 0.002	0.978 ± 0.002	0.978 ± 0.002	0.980 ± 0.002	0.999 ± 0.000
●		●	●			0.999 ± 0.000	0.969 ± 0.003	0.966 ± 0.003	0.966 ± 0.003	0.969 ± 0.003	0.998 ± 0.000
●		●	●		●	0.999 ± 0.000	0.976 ± 0.004	0.975 ± 0.004	0.975 ± 0.004	0.976 ± 0.004	0.999 ± 0.000
●		●	●	●		0.999 ± 0.001	0.976 ± 0.003	0.974 ± 0.002	0.974 ± 0.002	0.976 ± 0.003	0.999 ± 0.000
●		●	●	●	●	0.999 ± 0.000	0.980 ± 0.002	0.977 ± 0.002	0.977 ± 0.002	0.980 ± 0.002	0.999 ± 0.000
●	●					0.997 ± 0.001	0.962 ± 0.002	0.959 ± 0.003	0.959 ± 0.003	0.962 ± 0.002	0.998 ± 0.000
●	●				●	0.998 ± 0.002	0.969 ± 0.002	0.966 ± 0.003	0.966 ± 0.004	0.969 ± 0.002	0.998 ± 0.000
●	●			●		0.998 ± 0.001	0.975 ± 0.002	0.972 ± 0.002	0.970 ± 0.003	0.975 ± 0.002	0.999 ± 0.000
●	●			●	●	0.999 ± 0.000	0.977 ± 0.003	0.974 ± 0.003	0.973 ± 0.003	0.977 ± 0.003	0.999 ± 0.000
●	●		●			0.998 ± 0.001	0.965 ± 0.003	0.963 ± 0.003	0.963 ± 0.003	0.965 ± 0.003	0.998 ± 0.000
●	●		●		●	0.998 ± 0.001	0.970 ± 0.003	0.968 ± 0.003	0.968 ± 0.003	0.970 ± 0.003	0.998 ± 0.000
●	●		●	●		0.998 ± 0.001	0.973 ± 0.002	0.970 ± 0.002	0.969 ± 0.003	0.973 ± 0.002	0.999 ± 0.000
●	●		●	●	●	0.999 ± 0.000	0.977 ± 0.003	0.975 ± 0.003	0.974 ± 0.003	0.977 ± 0.003	0.999 ± 0.000
●	●	●				0.997 ± 0.001	0.962 ± 0.004	0.960 ± 0.005	0.959 ± 0.006	0.962 ± 0.004	0.998 ± 0.000
●	●	●			●	0.998 ± 0.001	0.968 ± 0.001	0.966 ± 0.002	0.966 ± 0.002	0.968 ± 0.001	0.998 ± 0.000
●	●	●		●		0.998 ± 0.001	0.971 ± 0.003	0.968 ± 0.004	0.968 ± 0.004	0.971 ± 0.003	0.998 ± 0.000
●	●	●		●	●	0.998 ± 0.001	0.975 ± 0.004	0.972 ± 0.004	0.972 ± 0.004	0.975 ± 0.004	0.999 ± 0.000
●	●	●	●			0.998 ± 0.000	0.966 ± 0.003	0.964 ± 0.003	0.964 ± 0.003	0.966 ± 0.003	0.998 ± 0.000
●	●	●	●		●	0.999 ± 0.000	0.970 ± 0.001	0.968 ± 0.001	0.968 ± 0.001	0.970 ± 0.001	0.998 ± 0.000
●	●	●	●	●		0.999 ± 0.000	0.972 ± 0.002	0.970 ± 0.002	0.970 ± 0.002	0.972 ± 0.002	0.999 ± 0.000
●	●	●	●	●	●	0.999 ± 0.000	0.975 ± 0.003	0.972 ± 0.002	0.971 ± 0.002	0.975 ± 0.003	0.999 ± 0.000

Table S13: Classification metrics for MOGONET with different omics combination on TCGA dataset

mRNA	nc mRNA	miRNA	CNV	DNAm	Protein	AUROC	Accuracy	F1	Precision	Recall	Specificity
				●	●	0.946 ± 0.000	0.986 ± 0.001	0.986 ± 0.001	0.987 ± 0.001	0.986 ± 0.001	0.999 ± 0.000
			●		●	0.946 ± 0.000	0.975 ± 0.004	0.975 ± 0.005	0.976 ± 0.004	0.975 ± 0.004	0.999 ± 0.000
			●	●		0.942 ± 0.001	0.919 ± 0.011	0.921 ± 0.011	0.927 ± 0.009	0.919 ± 0.011	0.996 ± 0.000
			●	●	●	0.946 ± 0.000	0.983 ± 0.004	0.983 ± 0.004	0.985 ± 0.003	0.983 ± 0.004	0.999 ± 0.000

Continued on next page

Table S13: Classification metrics for MOGONET with different omics combination

mRNA	nc mRNA	miRNA	CNV	DNAm	Protein	AUROC	Accuracy	F1	Precision	Recall	Specificity
		•			•	0.946 ± 0.001	0.966 ± 0.005	0.968 ± 0.005	0.971 ± 0.005	0.966 ± 0.005	0.998 ± 0.000
		•		•		0.943 ± 0.001	0.931 ± 0.006	0.934 ± 0.005	0.940 ± 0.005	0.931 ± 0.006	0.997 ± 0.000
		•		•	•	0.946 ± 0.001	0.980 ± 0.004	0.981 ± 0.004	0.983 ± 0.004	0.980 ± 0.004	0.999 ± 0.000
		•	•			0.936 ± 0.001	0.860 ± 0.009	0.862 ± 0.009	0.869 ± 0.008	0.860 ± 0.009	0.993 ± 0.001
		•	•		•	0.946 ± 0.000	0.971 ± 0.007	0.972 ± 0.008	0.975 ± 0.008	0.971 ± 0.007	0.999 ± 0.000
		•	•	•		0.943 ± 0.001	0.935 ± 0.005	0.938 ± 0.004	0.944 ± 0.004	0.935 ± 0.005	0.997 ± 0.000
	•				•	0.946 ± 0.001	0.981 ± 0.004	0.981 ± 0.004	0.982 ± 0.004	0.981 ± 0.004	0.999 ± 0.000
	•			•		0.943 ± 0.001	0.951 ± 0.003	0.952 ± 0.003	0.954 ± 0.004	0.951 ± 0.003	0.998 ± 0.000
	•			•	•	0.946 ± 0.001	0.981 ± 0.001	0.980 ± 0.001	0.980 ± 0.001	0.981 ± 0.001	0.999 ± 0.000
	•		•			0.940 ± 0.001	0.904 ± 0.009	0.908 ± 0.009	0.916 ± 0.009	0.904 ± 0.009	0.995 ± 0.000
	•		•		•	0.945 ± 0.000	0.977 ± 0.001	0.978 ± 0.002	0.980 ± 0.002	0.977 ± 0.001	0.999 ± 0.000
	•		•	•		0.944 ± 0.000	0.944 ± 0.005	0.946 ± 0.005	0.949 ± 0.005	0.944 ± 0.005	0.997 ± 0.000
	•	•				0.940 ± 0.001	0.909 ± 0.004	0.912 ± 0.004	0.917 ± 0.006	0.909 ± 0.004	0.996 ± 0.000
	•	•			•	0.946 ± 0.001	0.975 ± 0.006	0.975 ± 0.005	0.976 ± 0.004	0.975 ± 0.006	0.999 ± 0.000
	•	•		•		0.943 ± 0.001	0.944 ± 0.006	0.947 ± 0.005	0.952 ± 0.006	0.944 ± 0.006	0.997 ± 0.000
	•	•	•			0.941 ± 0.001	0.916 ± 0.006	0.919 ± 0.006	0.925 ± 0.007	0.916 ± 0.006	0.996 ± 0.000
•					•	0.946 ± 0.001	0.981 ± 0.004	0.983 ± 0.004	0.985 ± 0.003	0.981 ± 0.004	0.999 ± 0.000
•				•		0.945 ± 0.001	0.959 ± 0.002	0.961 ± 0.002	0.965 ± 0.003	0.959 ± 0.002	0.998 ± 0.000
•				•	•	0.946 ± 0.001	0.982 ± 0.001	0.983 ± 0.002	0.985 ± 0.002	0.982 ± 0.001	0.999 ± 0.000
•			•			0.943 ± 0.001	0.921 ± 0.011	0.926 ± 0.010	0.933 ± 0.009	0.921 ± 0.011	0.996 ± 0.001
•			•		•	0.946 ± 0.000	0.976 ± 0.003	0.978 ± 0.003	0.980 ± 0.002	0.976 ± 0.003	0.999 ± 0.000
•			•	•		0.945 ± 0.000	0.957 ± 0.003	0.958 ± 0.003	0.962 ± 0.002	0.957 ± 0.003	0.998 ± 0.000
•		•				0.943 ± 0.001	0.934 ± 0.005	0.938 ± 0.005	0.943 ± 0.004	0.934 ± 0.005	0.997 ± 0.000
•		•			•	0.946 ± 0.001	0.978 ± 0.003	0.979 ± 0.002	0.981 ± 0.001	0.978 ± 0.003	0.999 ± 0.000
•		•		•		0.944 ± 0.001	0.957 ± 0.005	0.959 ± 0.004	0.963 ± 0.003	0.957 ± 0.005	0.998 ± 0.000
•		•	•			0.942 ± 0.001	0.933 ± 0.006	0.936 ± 0.005	0.942 ± 0.005	0.933 ± 0.006	0.997 ± 0.000
•	•					0.943 ± 0.000	0.941 ± 0.009	0.942 ± 0.007	0.945 ± 0.005	0.941 ± 0.009	0.997 ± 0.000
•	•				•	0.945 ± 0.001	0.976 ± 0.003	0.975 ± 0.003	0.975 ± 0.002	0.976 ± 0.003	0.999 ± 0.000
•	•			•		0.944 ± 0.000	0.955 ± 0.004	0.955 ± 0.003	0.958 ± 0.002	0.955 ± 0.004	0.998 ± 0.000
•	•		•			0.943 ± 0.001	0.939 ± 0.003	0.940 ± 0.003	0.944 ± 0.003	0.939 ± 0.003	0.997 ± 0.000
•	•	•				0.942 ± 0.000	0.941 ± 0.007	0.943 ± 0.008	0.947 ± 0.010	0.941 ± 0.007	0.997 ± 0.000

Table S14: Classification metrics for P-Net with different omics combination on TCGA dataset

mRNA	nc mRNA	miRNA	CNV	DNAm	Protein	AUROC	Accuracy	F1	Precision	Recall	Specificity
			•	•		0.997 ± 0.001	0.927 ± 0.007	0.924 ± 0.008	0.923 ± 0.009	0.927 ± 0.007	0.996 ± 0.000
		•		•		0.997 ± 0.000	0.929 ± 0.007	0.927 ± 0.008	0.927 ± 0.009	0.929 ± 0.007	0.996 ± 0.000
		•	•			0.952 ± 0.005	0.661 ± 0.016	0.645 ± 0.016	0.645 ± 0.014	0.661 ± 0.016	0.980 ± 0.001

Continued on next page

Table S14: Classification metrics for P-Net with different omics combination

mRNA	nc mRNA	miRNA	CNV	DNAm	Protein	AUROC	Accuracy	F1	Precision	Recall	Specificity
		•	•	•		0.997 ± 0.000	0.924 ± 0.004	0.921 ± 0.005	0.919 ± 0.006	0.924 ± 0.004	0.996 ± 0.000
	•			•		0.997 ± 0.001	0.931 ± 0.003	0.930 ± 0.004	0.930 ± 0.005	0.931 ± 0.003	0.996 ± 0.000
	•		•			0.949 ± 0.003	0.643 ± 0.007	0.626 ± 0.009	0.636 ± 0.011	0.643 ± 0.007	0.978 ± 0.000
	•		•	•		0.997 ± 0.001	0.930 ± 0.009	0.928 ± 0.008	0.927 ± 0.007	0.930 ± 0.009	0.996 ± 0.000
	•	•		•		0.997 ± 0.000	0.929 ± 0.006	0.928 ± 0.007	0.928 ± 0.007	0.929 ± 0.006	0.996 ± 0.000
	•	•	•			0.954 ± 0.002	0.670 ± 0.017	0.660 ± 0.019	0.665 ± 0.017	0.670 ± 0.017	0.980 ± 0.001
	•	•	•	•		0.996 ± 0.000	0.929 ± 0.003	0.927 ± 0.002	0.926 ± 0.002	0.929 ± 0.003	0.996 ± 0.000
•				•		0.998 ± 0.000	0.961 ± 0.009	0.959 ± 0.007	0.959 ± 0.005	0.961 ± 0.009	0.998 ± 0.000
•			•			0.998 ± 0.001	0.948 ± 0.007	0.947 ± 0.008	0.947 ± 0.009	0.948 ± 0.007	0.997 ± 0.000
•			•	•		0.998 ± 0.001	0.951 ± 0.008	0.949 ± 0.009	0.947 ± 0.010	0.951 ± 0.008	0.997 ± 0.001
•		•				0.997 ± 0.002	0.951 ± 0.006	0.948 ± 0.007	0.947 ± 0.008	0.951 ± 0.006	0.997 ± 0.000
•		•		•		0.998 ± 0.001	0.954 ± 0.010	0.954 ± 0.009	0.955 ± 0.008	0.954 ± 0.010	0.998 ± 0.000
•		•	•			0.997 ± 0.001	0.947 ± 0.004	0.945 ± 0.004	0.944 ± 0.004	0.947 ± 0.004	0.997 ± 0.000
•		•	•	•		0.998 ± 0.000	0.959 ± 0.003	0.957 ± 0.002	0.955 ± 0.001	0.959 ± 0.003	0.998 ± 0.000
•	•					0.998 ± 0.001	0.946 ± 0.006	0.943 ± 0.005	0.943 ± 0.007	0.946 ± 0.006	0.997 ± 0.000
•	•			•		0.999 ± 0.001	0.962 ± 0.005	0.960 ± 0.005	0.959 ± 0.004	0.962 ± 0.005	0.998 ± 0.000
•	•		•			0.997 ± 0.001	0.945 ± 0.011	0.942 ± 0.012	0.941 ± 0.012	0.945 ± 0.011	0.997 ± 0.001
•	•		•	•		0.999 ± 0.000	0.956 ± 0.006	0.956 ± 0.005	0.957 ± 0.005	0.956 ± 0.006	0.998 ± 0.000
•	•	•				0.997 ± 0.001	0.949 ± 0.004	0.947 ± 0.004	0.946 ± 0.004	0.949 ± 0.004	0.997 ± 0.000
•	•	•		•		0.998 ± 0.001	0.956 ± 0.006	0.955 ± 0.006	0.954 ± 0.006	0.956 ± 0.006	0.998 ± 0.000
•	•	•	•			0.997 ± 0.000	0.950 ± 0.002	0.947 ± 0.003	0.946 ± 0.004	0.950 ± 0.002	0.997 ± 0.000
•	•	•	•	•		0.998 ± 0.001	0.959 ± 0.006	0.956 ± 0.006	0.955 ± 0.006	0.959 ± 0.006	0.998 ± 0.000

C.2. Models performances on various combinations of omics on CCLE dataset

Table S15: Classification metrics for AttOmics with different omics combination on CCLE dataset.

mRNA	miRNA	CNV	DNAm	Protein	Metabolomics	AUROC	Accuracy	F1	Precision	Recall	Specificity
					•	0.894 ± 0.005	0.481 ± 0.039	0.460 ± 0.035	0.481 ± 0.029	0.481 ± 0.039	0.965 ± 0.003
				•		0.932 ± 0.005	0.669 ± 0.015	0.650 ± 0.023	0.671 ± 0.025	0.669 ± 0.015	0.977 ± 0.001
			•			0.971 ± 0.006	0.769 ± 0.032	0.759 ± 0.028	0.793 ± 0.027	0.769 ± 0.032	0.985 ± 0.002
		•				0.914 ± 0.011	0.577 ± 0.030	0.565 ± 0.032	0.585 ± 0.043	0.577 ± 0.030	0.972 ± 0.002
	•					0.947 ± 0.007	0.727 ± 0.024	0.705 ± 0.023	0.704 ± 0.022	0.727 ± 0.024	0.981 ± 0.002
•						0.970 ± 0.007	0.766 ± 0.024	0.757 ± 0.025	0.787 ± 0.023	0.766 ± 0.024	0.984 ± 0.002

Table S16: Classification metrics for AttOmics EF with different omics combination on CCLE dataset.

mRNA	miRNA	CNV	DNAm	Protein	Metabolomics	AUROC	Accuracy	F1	Precision	Recall	Specificity
				•	•	0.881 ± 0.016	0.465 ± 0.053	0.433 ± 0.058	0.466 ± 0.077	0.465 ± 0.053	0.964 ± 0.003
			•		•	0.984 ± 0.003	0.784 ± 0.048	0.777 ± 0.048	0.798 ± 0.049	0.784 ± 0.048	0.986 ± 0.004
			•	•		0.984 ± 0.003	0.797 ± 0.016	0.791 ± 0.019	0.822 ± 0.022	0.797 ± 0.016	0.987 ± 0.001
			•	•	•	0.983 ± 0.004	0.780 ± 0.034	0.778 ± 0.034	0.801 ± 0.041	0.780 ± 0.034	0.986 ± 0.002
		•			•	0.917 ± 0.008	0.577 ± 0.037	0.547 ± 0.032	0.544 ± 0.032	0.577 ± 0.037	0.971 ± 0.003
		•		•		0.922 ± 0.006	0.576 ± 0.024	0.544 ± 0.027	0.544 ± 0.040	0.576 ± 0.024	0.971 ± 0.002
		•		•	•	0.923 ± 0.007	0.613 ± 0.033	0.589 ± 0.034	0.594 ± 0.040	0.613 ± 0.033	0.974 ± 0.002
		•	•			0.973 ± 0.005	0.753 ± 0.033	0.732 ± 0.033	0.743 ± 0.030	0.753 ± 0.033	0.984 ± 0.002
		•	•		•	0.972 ± 0.004	0.753 ± 0.027	0.734 ± 0.033	0.752 ± 0.040	0.753 ± 0.027	0.984 ± 0.002
		•	•	•		0.975 ± 0.002	0.752 ± 0.024	0.732 ± 0.026	0.752 ± 0.041	0.752 ± 0.024	0.983 ± 0.002
		•	•	•	•	0.973 ± 0.007	0.757 ± 0.012	0.731 ± 0.011	0.737 ± 0.024	0.757 ± 0.012	0.984 ± 0.001
	•				•	0.913 ± 0.002	0.575 ± 0.029	0.553 ± 0.030	0.591 ± 0.045	0.575 ± 0.029	0.971 ± 0.002
	•			•		0.927 ± 0.014	0.628 ± 0.061	0.606 ± 0.077	0.613 ± 0.090	0.628 ± 0.061	0.974 ± 0.004
	•			•	•	0.939 ± 0.006	0.657 ± 0.035	0.643 ± 0.049	0.659 ± 0.064	0.657 ± 0.035	0.977 ± 0.003
	•		•			0.982 ± 0.006	0.793 ± 0.012	0.790 ± 0.016	0.816 ± 0.030	0.793 ± 0.012	0.987 ± 0.001
	•		•		•	0.982 ± 0.004	0.777 ± 0.044	0.775 ± 0.042	0.795 ± 0.043	0.777 ± 0.044	0.986 ± 0.003
	•		•	•		0.984 ± 0.003	0.794 ± 0.017	0.787 ± 0.015	0.810 ± 0.016	0.794 ± 0.017	0.987 ± 0.001
	•		•	•	•	0.985 ± 0.002	0.790 ± 0.016	0.786 ± 0.016	0.808 ± 0.013	0.790 ± 0.016	0.987 ± 0.001
	•	•				0.918 ± 0.011	0.563 ± 0.049	0.541 ± 0.055	0.551 ± 0.068	0.563 ± 0.049	0.971 ± 0.004
	•	•			•	0.921 ± 0.013	0.584 ± 0.032	0.562 ± 0.028	0.576 ± 0.020	0.584 ± 0.032	0.972 ± 0.002
	•	•		•		0.923 ± 0.009	0.579 ± 0.025	0.552 ± 0.021	0.551 ± 0.022	0.579 ± 0.025	0.971 ± 0.001
	•	•		•	•	0.927 ± 0.007	0.562 ± 0.019	0.528 ± 0.019	0.531 ± 0.029	0.562 ± 0.019	0.970 ± 0.002
	•	•	•			0.970 ± 0.003	0.759 ± 0.046	0.736 ± 0.062	0.764 ± 0.062	0.759 ± 0.046	0.984 ± 0.003
	•	•	•		•	0.968 ± 0.007	0.737 ± 0.023	0.714 ± 0.020	0.737 ± 0.034	0.737 ± 0.023	0.982 ± 0.002
	•	•	•	•		0.972 ± 0.005	0.754 ± 0.025	0.733 ± 0.031	0.751 ± 0.044	0.754 ± 0.025	0.983 ± 0.001
	•	•	•	•	•	0.970 ± 0.002	0.753 ± 0.027	0.736 ± 0.035	0.747 ± 0.042	0.753 ± 0.027	0.983 ± 0.002
•					•	0.967 ± 0.007	0.764 ± 0.033	0.759 ± 0.035	0.792 ± 0.045	0.764 ± 0.033	0.984 ± 0.002
•				•		0.969 ± 0.004	0.751 ± 0.027	0.739 ± 0.030	0.764 ± 0.050	0.751 ± 0.027	0.983 ± 0.002
•				•	•	0.969 ± 0.004	0.761 ± 0.014	0.755 ± 0.014	0.801 ± 0.024	0.761 ± 0.014	0.984 ± 0.001
•			•			0.984 ± 0.006	0.818 ± 0.032	0.818 ± 0.028	0.855 ± 0.023	0.818 ± 0.032	0.988 ± 0.002
•			•		•	0.984 ± 0.005	0.820 ± 0.013	0.825 ± 0.016	0.853 ± 0.027	0.820 ± 0.013	0.989 ± 0.001
•			•	•		0.984 ± 0.006	0.806 ± 0.012	0.806 ± 0.012	0.826 ± 0.012	0.806 ± 0.012	0.987 ± 0.001
•			•	•	•	0.988 ± 0.002	0.811 ± 0.036	0.808 ± 0.036	0.830 ± 0.040	0.811 ± 0.036	0.988 ± 0.002
•		•				0.946 ± 0.010	0.691 ± 0.031	0.659 ± 0.025	0.650 ± 0.029	0.691 ± 0.031	0.979 ± 0.002
•		•			•	0.955 ± 0.005	0.695 ± 0.025	0.670 ± 0.026	0.672 ± 0.039	0.695 ± 0.025	0.979 ± 0.001
•		•		•		0.956 ± 0.007	0.709 ± 0.014	0.685 ± 0.024	0.681 ± 0.033	0.709 ± 0.014	0.980 ± 0.001
•		•		•	•	0.958 ± 0.005	0.707 ± 0.021	0.682 ± 0.030	0.680 ± 0.038	0.707 ± 0.021	0.980 ± 0.002
•		•	•			0.977 ± 0.005	0.751 ± 0.029	0.736 ± 0.033	0.761 ± 0.034	0.751 ± 0.029	0.983 ± 0.002
•		•	•		•	0.972 ± 0.006	0.765 ± 0.037	0.749 ± 0.038	0.769 ± 0.040	0.765 ± 0.037	0.985 ± 0.002
•		•	•	•		0.975 ± 0.007	0.774 ± 0.038	0.757 ± 0.041	0.776 ± 0.031	0.774 ± 0.038	0.985 ± 0.002

Continued on next page

Table S16: Classification metrics for AttOmics EF with different omics combination on CCLE dataset.

mRNA	miRNA	CNV	DNAm	Protein	Metabolomics	AUROC	Accuracy	F1	Precision	Recall	Specificity
•		•	•	•	•	0.972 ± 0.003	0.767 ± 0.023	0.755 ± 0.029	0.787 ± 0.046	0.767 ± 0.023	0.985 ± 0.002
•	•					0.964 ± 0.010	0.764 ± 0.020	0.761 ± 0.020	0.794 ± 0.026	0.764 ± 0.020	0.984 ± 0.001
•	•				•	0.973 ± 0.003	0.773 ± 0.011	0.771 ± 0.012	0.796 ± 0.010	0.773 ± 0.011	0.985 ± 0.001
•	•			•		0.967 ± 0.007	0.783 ± 0.017	0.776 ± 0.020	0.800 ± 0.034	0.783 ± 0.017	0.985 ± 0.002
•	•			•	•	0.965 ± 0.005	0.759 ± 0.023	0.754 ± 0.019	0.776 ± 0.029	0.759 ± 0.023	0.984 ± 0.001
•	•		•			0.986 ± 0.004	0.800 ± 0.037	0.800 ± 0.036	0.824 ± 0.030	0.800 ± 0.037	0.987 ± 0.002
•	•		•		•	0.987 ± 0.005	0.817 ± 0.020	0.813 ± 0.021	0.834 ± 0.027	0.817 ± 0.020	0.988 ± 0.001
•	•		•	•		0.985 ± 0.002	0.795 ± 0.016	0.791 ± 0.017	0.814 ± 0.015	0.795 ± 0.016	0.987 ± 0.001
•	•		•	•	•	0.987 ± 0.002	0.814 ± 0.021	0.812 ± 0.022	0.834 ± 0.022	0.814 ± 0.021	0.988 ± 0.002
•	•	•				0.950 ± 0.013	0.668 ± 0.041	0.637 ± 0.048	0.646 ± 0.054	0.668 ± 0.041	0.977 ± 0.004
•	•	•			•	0.952 ± 0.010	0.662 ± 0.027	0.631 ± 0.038	0.635 ± 0.028	0.662 ± 0.027	0.977 ± 0.003
•	•	•		•		0.956 ± 0.006	0.701 ± 0.028	0.680 ± 0.031	0.675 ± 0.037	0.701 ± 0.028	0.980 ± 0.002
•	•	•		•	•	0.960 ± 0.006	0.719 ± 0.024	0.686 ± 0.028	0.668 ± 0.033	0.719 ± 0.024	0.981 ± 0.002
•	•	•	•			0.977 ± 0.003	0.764 ± 0.026	0.753 ± 0.030	0.764 ± 0.038	0.764 ± 0.026	0.985 ± 0.002
•	•	•	•		•	0.975 ± 0.007	0.786 ± 0.029	0.765 ± 0.033	0.789 ± 0.035	0.786 ± 0.029	0.986 ± 0.002
•	•	•	•	•		0.977 ± 0.001	0.770 ± 0.016	0.763 ± 0.017	0.779 ± 0.029	0.770 ± 0.016	0.985 ± 0.001
•	•	•	•	•	•	0.976 ± 0.003	0.774 ± 0.036	0.752 ± 0.039	0.765 ± 0.036	0.774 ± 0.036	0.985 ± 0.002

Table S17: Classification metrics for AttOmics IF with different omics combination on CCLE dataset.

mRNA	miRNA	CNV	DNAm	Protein	Metabolomics	AUROC	Accuracy	F1	Precision	Recall	Specificity
				•	•	0.956 ± 0.011	0.714 ± 0.046	0.701 ± 0.054	0.716 ± 0.070	0.714 ± 0.046	0.980 ± 0.003
			•		•	0.984 ± 0.004	0.813 ± 0.022	0.804 ± 0.025	0.819 ± 0.030	0.813 ± 0.022	0.988 ± 0.002
			•	•		0.984 ± 0.004	0.826 ± 0.030	0.820 ± 0.034	0.847 ± 0.032	0.826 ± 0.030	0.989 ± 0.002
			•	•	•	0.985 ± 0.008	0.823 ± 0.031	0.817 ± 0.034	0.840 ± 0.033	0.823 ± 0.031	0.989 ± 0.002
		•			•	0.929 ± 0.017	0.621 ± 0.026	0.579 ± 0.033	0.582 ± 0.039	0.621 ± 0.026	0.974 ± 0.003
		•		•		0.955 ± 0.013	0.698 ± 0.023	0.667 ± 0.035	0.693 ± 0.052	0.698 ± 0.023	0.979 ± 0.002
		•		•	•	0.952 ± 0.007	0.721 ± 0.037	0.686 ± 0.045	0.692 ± 0.032	0.721 ± 0.037	0.981 ± 0.003
		•	•			0.985 ± 0.007	0.844 ± 0.026	0.836 ± 0.031	0.865 ± 0.027	0.844 ± 0.026	0.990 ± 0.002
		•	•		•	0.988 ± 0.005	0.847 ± 0.038	0.829 ± 0.049	0.848 ± 0.051	0.847 ± 0.038	0.990 ± 0.003
		•	•	•		0.986 ± 0.006	0.851 ± 0.018	0.839 ± 0.025	0.859 ± 0.040	0.851 ± 0.018	0.991 ± 0.001
		•	•	•	•	0.986 ± 0.004	0.854 ± 0.034	0.844 ± 0.038	0.867 ± 0.040	0.854 ± 0.034	0.991 ± 0.002
	•				•	0.939 ± 0.007	0.700 ± 0.025	0.688 ± 0.050	0.720 ± 0.079	0.700 ± 0.025	0.979 ± 0.002
	•			•		0.949 ± 0.009	0.705 ± 0.028	0.686 ± 0.032	0.695 ± 0.038	0.705 ± 0.028	0.980 ± 0.002
	•			•	•	0.957 ± 0.013	0.715 ± 0.016	0.706 ± 0.011	0.726 ± 0.029	0.715 ± 0.016	0.980 ± 0.001
	•		•			0.985 ± 0.004	0.822 ± 0.012	0.814 ± 0.016	0.839 ± 0.023	0.822 ± 0.012	0.989 ± 0.000
	•		•		•	0.986 ± 0.003	0.836 ± 0.016	0.833 ± 0.019	0.856 ± 0.022	0.836 ± 0.016	0.990 ± 0.001
	•		•	•		0.983 ± 0.007	0.825 ± 0.013	0.820 ± 0.014	0.843 ± 0.011	0.825 ± 0.013	0.989 ± 0.001

Continued on next page

Table S17: Classification metrics for AttOmics IF with different omics combination on CCLE dataset.

mRNA	miRNA	CNV	DNAm	Protein	Metabolomics	AUROC	Accuracy	F1	Precision	Recall	Specificity
	•		•	•	•	0.984 ± 0.008	0.811 ± 0.035	0.798 ± 0.035	0.826 ± 0.043	0.811 ± 0.035	0.988 ± 0.002
	•	•				0.952 ± 0.013	0.707 ± 0.049	0.677 ± 0.061	0.680 ± 0.066	0.707 ± 0.049	0.980 ± 0.004
	•	•			•	0.957 ± 0.013	0.741 ± 0.035	0.707 ± 0.035	0.703 ± 0.040	0.741 ± 0.035	0.982 ± 0.002
	•	•		•		0.962 ± 0.011	0.751 ± 0.028	0.732 ± 0.031	0.751 ± 0.055	0.751 ± 0.028	0.984 ± 0.002
	•	•		•	•	0.967 ± 0.006	0.753 ± 0.017	0.727 ± 0.013	0.716 ± 0.013	0.753 ± 0.017	0.983 ± 0.001
	•	•	•			0.989 ± 0.003	0.854 ± 0.027	0.844 ± 0.034	0.875 ± 0.028	0.854 ± 0.027	0.991 ± 0.002
	•	•	•		•	0.987 ± 0.009	0.849 ± 0.030	0.842 ± 0.031	0.870 ± 0.030	0.849 ± 0.030	0.991 ± 0.002
	•	•	•	•		0.990 ± 0.004	0.847 ± 0.029	0.834 ± 0.033	0.873 ± 0.024	0.847 ± 0.029	0.991 ± 0.002
	•	•	•	•	•	0.988 ± 0.004	0.866 ± 0.013	0.856 ± 0.019	0.885 ± 0.015	0.866 ± 0.013	0.992 ± 0.001
•					•	0.977 ± 0.009	0.785 ± 0.021	0.783 ± 0.023	0.814 ± 0.032	0.785 ± 0.021	0.985 ± 0.002
•				•		0.975 ± 0.006	0.779 ± 0.032	0.782 ± 0.037	0.813 ± 0.045	0.779 ± 0.032	0.985 ± 0.002
•				•	•	0.981 ± 0.004	0.808 ± 0.031	0.810 ± 0.030	0.839 ± 0.036	0.808 ± 0.031	0.987 ± 0.002
•			•			0.992 ± 0.004	0.868 ± 0.024	0.859 ± 0.029	0.888 ± 0.028	0.868 ± 0.024	0.991 ± 0.002
•			•		•	0.991 ± 0.005	0.870 ± 0.036	0.870 ± 0.038	0.892 ± 0.046	0.870 ± 0.036	0.992 ± 0.003
•			•	•		0.993 ± 0.007	0.883 ± 0.038	0.880 ± 0.042	0.891 ± 0.044	0.883 ± 0.038	0.992 ± 0.003
•			•	•	•	0.991 ± 0.005	0.853 ± 0.030	0.849 ± 0.032	0.876 ± 0.028	0.853 ± 0.030	0.991 ± 0.002
•		•				0.980 ± 0.006	0.809 ± 0.033	0.796 ± 0.036	0.816 ± 0.058	0.809 ± 0.033	0.987 ± 0.002
•		•			•	0.981 ± 0.008	0.821 ± 0.009	0.810 ± 0.012	0.826 ± 0.024	0.821 ± 0.009	0.988 ± 0.001
•		•		•		0.980 ± 0.006	0.819 ± 0.021	0.807 ± 0.023	0.830 ± 0.039	0.819 ± 0.021	0.988 ± 0.001
•		•		•	•	0.981 ± 0.011	0.801 ± 0.066	0.785 ± 0.089	0.809 ± 0.101	0.801 ± 0.066	0.987 ± 0.005
•		•	•			0.991 ± 0.004	0.858 ± 0.016	0.855 ± 0.018	0.889 ± 0.027	0.858 ± 0.016	0.991 ± 0.001
•		•	•		•	0.990 ± 0.003	0.862 ± 0.026	0.855 ± 0.031	0.880 ± 0.026	0.862 ± 0.026	0.991 ± 0.002
•		•	•	•		0.994 ± 0.003	0.883 ± 0.015	0.877 ± 0.021	0.899 ± 0.020	0.883 ± 0.015	0.993 ± 0.001
•		•	•	•	•	0.995 ± 0.004	0.888 ± 0.031	0.880 ± 0.034	0.899 ± 0.030	0.888 ± 0.031	0.993 ± 0.002
•	•					0.977 ± 0.006	0.781 ± 0.031	0.783 ± 0.030	0.816 ± 0.038	0.781 ± 0.031	0.986 ± 0.002
•	•				•	0.976 ± 0.008	0.768 ± 0.020	0.763 ± 0.022	0.792 ± 0.034	0.768 ± 0.020	0.985 ± 0.001
•	•			•		0.978 ± 0.006	0.796 ± 0.031	0.791 ± 0.037	0.806 ± 0.040	0.796 ± 0.031	0.987 ± 0.002
•	•			•	•	0.983 ± 0.003	0.811 ± 0.035	0.810 ± 0.038	0.847 ± 0.034	0.811 ± 0.035	0.987 ± 0.003
•	•		•			0.992 ± 0.004	0.859 ± 0.015	0.853 ± 0.019	0.877 ± 0.016	0.859 ± 0.015	0.991 ± 0.001
•	•		•		•	0.991 ± 0.002	0.865 ± 0.008	0.862 ± 0.009	0.883 ± 0.018	0.865 ± 0.008	0.991 ± 0.001
•	•		•	•		0.991 ± 0.003	0.869 ± 0.033	0.865 ± 0.038	0.888 ± 0.027	0.869 ± 0.033	0.992 ± 0.002
•	•		•	•	•	0.993 ± 0.004	0.879 ± 0.022	0.874 ± 0.028	0.896 ± 0.027	0.879 ± 0.022	0.992 ± 0.002
•	•	•				0.974 ± 0.006	0.804 ± 0.037	0.794 ± 0.041	0.818 ± 0.053	0.804 ± 0.037	0.987 ± 0.002
•	•	•			•	0.982 ± 0.005	0.803 ± 0.045	0.798 ± 0.050	0.827 ± 0.059	0.803 ± 0.045	0.987 ± 0.003
•	•	•		•		0.987 ± 0.004	0.843 ± 0.028	0.840 ± 0.030	0.876 ± 0.020	0.843 ± 0.028	0.990 ± 0.002
•	•	•		•	•	0.981 ± 0.003	0.823 ± 0.029	0.812 ± 0.031	0.832 ± 0.038	0.823 ± 0.029	0.988 ± 0.002
•	•	•	•			0.991 ± 0.003	0.875 ± 0.023	0.870 ± 0.026	0.895 ± 0.028	0.875 ± 0.023	0.992 ± 0.002
•	•	•	•		•	0.990 ± 0.006	0.853 ± 0.019	0.847 ± 0.025	0.870 ± 0.021	0.853 ± 0.019	0.991 ± 0.001
•	•	•	•	•		0.994 ± 0.004	0.882 ± 0.040	0.875 ± 0.043	0.898 ± 0.039	0.882 ± 0.040	0.992 ± 0.002
•	•	•	•	•	•	0.990 ± 0.006	0.865 ± 0.019	0.856 ± 0.020	0.880 ± 0.010	0.865 ± 0.019	0.991 ± 0.001

Table S18: Classification metrics for CrossAttOmics with different omics combination on CCLE dataset.

mRNA	miRNA	CNV	DNAm	Protein	Metabolomics	AUROC	Accuracy	F1	Precision	Recall	Specificity
	•		•			0.982 ± 0.007	0.790 ± 0.022	0.780 ± 0.026	0.801 ± 0.030	0.790 ± 0.022	0.987 ± 0.002
	•		•		•	0.975 ± 0.014	0.809 ± 0.032	0.801 ± 0.031	0.810 ± 0.032	0.809 ± 0.032	0.988 ± 0.003
	•		•	•		0.978 ± 0.008	0.780 ± 0.037	0.765 ± 0.045	0.787 ± 0.058	0.780 ± 0.037	0.986 ± 0.003
	•		•	•	•	0.975 ± 0.004	0.789 ± 0.018	0.781 ± 0.014	0.801 ± 0.010	0.789 ± 0.018	0.987 ± 0.002
	•	•				0.924 ± 0.007	0.656 ± 0.032	0.612 ± 0.034	0.618 ± 0.059	0.656 ± 0.032	0.976 ± 0.002
	•	•			•	0.934 ± 0.013	0.669 ± 0.059	0.635 ± 0.061	0.645 ± 0.066	0.669 ± 0.059	0.977 ± 0.004
	•	•		•		0.953 ± 0.008	0.716 ± 0.023	0.688 ± 0.031	0.697 ± 0.033	0.716 ± 0.023	0.980 ± 0.001
	•	•		•	•	0.953 ± 0.009	0.722 ± 0.044	0.688 ± 0.044	0.683 ± 0.048	0.722 ± 0.044	0.981 ± 0.003
	•	•	•			0.983 ± 0.005	0.833 ± 0.022	0.822 ± 0.030	0.848 ± 0.030	0.833 ± 0.022	0.989 ± 0.002
	•	•	•		•	0.978 ± 0.005	0.831 ± 0.017	0.825 ± 0.020	0.845 ± 0.015	0.831 ± 0.017	0.989 ± 0.001
	•	•	•	•		0.984 ± 0.005	0.850 ± 0.029	0.841 ± 0.030	0.873 ± 0.024	0.850 ± 0.029	0.991 ± 0.002
	•	•	•	•	•	0.981 ± 0.006	0.834 ± 0.013	0.825 ± 0.016	0.851 ± 0.019	0.834 ± 0.013	0.989 ± 0.001
•					•	0.965 ± 0.007	0.767 ± 0.031	0.755 ± 0.032	0.776 ± 0.048	0.767 ± 0.031	0.984 ± 0.002
•				•		0.966 ± 0.011	0.749 ± 0.018	0.737 ± 0.022	0.759 ± 0.023	0.749 ± 0.018	0.983 ± 0.002
•				•	•	0.967 ± 0.005	0.763 ± 0.055	0.746 ± 0.062	0.771 ± 0.055	0.763 ± 0.055	0.983 ± 0.004
•			•			0.985 ± 0.006	0.834 ± 0.037	0.829 ± 0.043	0.862 ± 0.046	0.834 ± 0.037	0.989 ± 0.003
•			•		•	0.981 ± 0.007	0.849 ± 0.022	0.843 ± 0.032	0.865 ± 0.025	0.849 ± 0.022	0.990 ± 0.002
•			•	•		0.989 ± 0.003	0.842 ± 0.028	0.831 ± 0.031	0.855 ± 0.035	0.842 ± 0.028	0.990 ± 0.001
•			•	•	•	0.985 ± 0.005	0.839 ± 0.028	0.831 ± 0.033	0.855 ± 0.039	0.839 ± 0.028	0.989 ± 0.002
•		•				0.967 ± 0.015	0.799 ± 0.025	0.782 ± 0.032	0.798 ± 0.043	0.799 ± 0.025	0.986 ± 0.002
•		•			•	0.973 ± 0.009	0.770 ± 0.039	0.756 ± 0.046	0.778 ± 0.038	0.770 ± 0.039	0.984 ± 0.003
•		•		•		0.973 ± 0.004	0.786 ± 0.025	0.768 ± 0.034	0.783 ± 0.047	0.786 ± 0.025	0.985 ± 0.001
•		•		•	•	0.966 ± 0.007	0.776 ± 0.029	0.743 ± 0.037	0.766 ± 0.042	0.776 ± 0.029	0.984 ± 0.002
•		•	•			0.983 ± 0.007	0.828 ± 0.029	0.810 ± 0.032	0.834 ± 0.037	0.828 ± 0.029	0.988 ± 0.002
•		•	•		•	0.985 ± 0.013	0.841 ± 0.017	0.832 ± 0.025	0.845 ± 0.038	0.841 ± 0.017	0.989 ± 0.001
•		•	•	•		0.986 ± 0.005	0.846 ± 0.036	0.837 ± 0.045	0.856 ± 0.063	0.846 ± 0.036	0.990 ± 0.002
•		•	•	•	•	0.988 ± 0.006	0.847 ± 0.017	0.838 ± 0.023	0.845 ± 0.038	0.847 ± 0.017	0.990 ± 0.002
•	•					0.966 ± 0.007	0.764 ± 0.024	0.748 ± 0.025	0.759 ± 0.030	0.764 ± 0.024	0.983 ± 0.002
•	•				•	0.972 ± 0.002	0.781 ± 0.029	0.773 ± 0.031	0.793 ± 0.035	0.781 ± 0.029	0.985 ± 0.001
•	•			•		0.968 ± 0.003	0.770 ± 0.011	0.759 ± 0.013	0.785 ± 0.012	0.770 ± 0.011	0.984 ± 0.001
•	•			•	•	0.968 ± 0.005	0.795 ± 0.017	0.785 ± 0.018	0.802 ± 0.022	0.795 ± 0.017	0.985 ± 0.001
•	•		•			0.987 ± 0.003	0.842 ± 0.025	0.835 ± 0.028	0.856 ± 0.033	0.842 ± 0.025	0.989 ± 0.002
•	•		•		•	0.988 ± 0.003	0.834 ± 0.018	0.832 ± 0.016	0.862 ± 0.013	0.834 ± 0.018	0.989 ± 0.001
•	•		•	•		0.990 ± 0.002	0.844 ± 0.021	0.837 ± 0.025	0.858 ± 0.031	0.844 ± 0.021	0.990 ± 0.002
•	•		•	•	•	0.987 ± 0.002	0.878 ± 0.030	0.873 ± 0.028	0.893 ± 0.028	0.878 ± 0.030	0.992 ± 0.002
•	•	•				0.974 ± 0.005	0.821 ± 0.043	0.808 ± 0.046	0.827 ± 0.044	0.821 ± 0.043	0.987 ± 0.002
•	•	•			•	0.969 ± 0.015	0.787 ± 0.027	0.767 ± 0.050	0.788 ± 0.055	0.787 ± 0.027	0.985 ± 0.003
•	•	•		•		0.975 ± 0.011	0.781 ± 0.046	0.768 ± 0.048	0.807 ± 0.037	0.781 ± 0.046	0.985 ± 0.003
•	•	•		•	•	0.969 ± 0.005	0.767 ± 0.015	0.755 ± 0.019	0.777 ± 0.045	0.767 ± 0.015	0.985 ± 0.001
•	•	•	•			0.989 ± 0.002	0.857 ± 0.008	0.851 ± 0.008	0.875 ± 0.014	0.857 ± 0.008	0.991 ± 0.001

Continued on next page

Table S18: Classification metrics for CrossAttOmics with different omics combination on CCLE dataset.

mRNA	miRNA	CNV	DNAm	Protein	Metabolomics	AUROC	Accuracy	F1	Precision	Recall	Specificity
•	•	•	•		•	0.991 ± 0.002	0.854 ± 0.034	0.842 ± 0.042	0.865 ± 0.038	0.854 ± 0.034	0.990 ± 0.002
•	•	•	•	•		0.987 ± 0.006	0.866 ± 0.020	0.860 ± 0.019	0.883 ± 0.020	0.866 ± 0.020	0.991 ± 0.001
•	•	•	•	•	•	0.990 ± 0.002	0.866 ± 0.029	0.863 ± 0.033	0.891 ± 0.026	0.866 ± 0.029	0.991 ± 0.002

Table S19: Classification metrics for MLP with different omics combination on CCLE dataset.

mRNA	miRNA	CNV	DNAm	Protein	Metabolomics	AUROC	Accuracy	F1	Precision	Recall	Specificity
					•	0.910 ± 0.015	0.570 ± 0.055	0.541 ± 0.066	0.565 ± 0.073	0.570 ± 0.055	0.970 ± 0.004
				•		0.951 ± 0.008	0.715 ± 0.025	0.698 ± 0.029	0.698 ± 0.027	0.715 ± 0.025	0.980 ± 0.002
			•			0.982 ± 0.003	0.791 ± 0.014	0.784 ± 0.016	0.818 ± 0.013	0.791 ± 0.014	0.987 ± 0.001
		•				0.891 ± 0.007	0.462 ± 0.023	0.447 ± 0.017	0.495 ± 0.023	0.462 ± 0.023	0.964 ± 0.001
	•					0.934 ± 0.006	0.731 ± 0.023	0.723 ± 0.036	0.736 ± 0.045	0.731 ± 0.023	0.981 ± 0.002
•						0.913 ± 0.020	0.711 ± 0.025	0.705 ± 0.017	0.727 ± 0.021	0.711 ± 0.025	0.980 ± 0.002

Table S20: Classification metrics for MLP EF with different omics combination on CCLE dataset.

mRNA	miRNA	CNV	DNAm	Protein	Metabolomics	AUROC	Accuracy	F1	Precision	Recall	Specificity
			•		•	0.978 ± 0.009	0.784 ± 0.021	0.773 ± 0.023	0.793 ± 0.022	0.784 ± 0.021	0.987 ± 0.001
			•	•		0.980 ± 0.006	0.799 ± 0.008	0.792 ± 0.008	0.824 ± 0.014	0.799 ± 0.008	0.987 ± 0.001
			•	•	•	0.984 ± 0.005	0.796 ± 0.018	0.788 ± 0.020	0.817 ± 0.023	0.796 ± 0.018	0.988 ± 0.001
		•			•	0.890 ± 0.005	0.502 ± 0.036	0.487 ± 0.028	0.520 ± 0.024	0.502 ± 0.036	0.967 ± 0.002
		•		•		0.904 ± 0.007	0.502 ± 0.034	0.480 ± 0.036	0.511 ± 0.046	0.502 ± 0.034	0.968 ± 0.002
		•		•	•	0.903 ± 0.015	0.512 ± 0.033	0.489 ± 0.042	0.524 ± 0.035	0.512 ± 0.033	0.967 ± 0.003
		•	•			0.974 ± 0.004	0.769 ± 0.018	0.749 ± 0.024	0.765 ± 0.042	0.769 ± 0.018	0.985 ± 0.001
		•	•		•	0.979 ± 0.001	0.759 ± 0.021	0.736 ± 0.019	0.751 ± 0.037	0.759 ± 0.021	0.984 ± 0.001
		•	•	•		0.974 ± 0.004	0.768 ± 0.028	0.759 ± 0.032	0.780 ± 0.037	0.768 ± 0.028	0.985 ± 0.002
		•	•	•	•	0.977 ± 0.005	0.776 ± 0.015	0.761 ± 0.017	0.774 ± 0.034	0.776 ± 0.015	0.985 ± 0.001
	•				•	0.946 ± 0.004	0.770 ± 0.013	0.768 ± 0.013	0.813 ± 0.009	0.770 ± 0.013	0.984 ± 0.001
	•			•		0.956 ± 0.004	0.774 ± 0.010	0.766 ± 0.012	0.772 ± 0.017	0.774 ± 0.010	0.985 ± 0.001
	•			•	•	0.961 ± 0.006	0.776 ± 0.031	0.758 ± 0.031	0.755 ± 0.030	0.776 ± 0.031	0.985 ± 0.002
	•		•			0.982 ± 0.005	0.821 ± 0.018	0.814 ± 0.019	0.839 ± 0.029	0.821 ± 0.018	0.989 ± 0.001
	•		•		•	0.981 ± 0.004	0.809 ± 0.024	0.800 ± 0.023	0.822 ± 0.024	0.809 ± 0.024	0.988 ± 0.002
	•		•	•		0.983 ± 0.006	0.809 ± 0.031	0.803 ± 0.029	0.822 ± 0.032	0.809 ± 0.031	0.988 ± 0.002
	•		•	•	•	0.980 ± 0.006	0.817 ± 0.010	0.808 ± 0.007	0.835 ± 0.017	0.817 ± 0.010	0.989 ± 0.001
	•	•				0.887 ± 0.017	0.481 ± 0.047	0.465 ± 0.036	0.515 ± 0.038	0.481 ± 0.047	0.965 ± 0.003

Continued on next page

Table S20: Classification metrics for MLP EF with different omics combination on CCLE dataset.

mRNA	miRNA	CNV	DNAm	Protein	Metabolomics	AUROC	Accuracy	F1	Precision	Recall	Specificity
	•	•			•	0.889 ± 0.024	0.492 ± 0.041	0.470 ± 0.065	0.500 ± 0.099	0.492 ± 0.041	0.966 ± 0.004
	•	•		•		0.906 ± 0.008	0.554 ± 0.032	0.525 ± 0.038	0.565 ± 0.037	0.554 ± 0.032	0.970 ± 0.003
	•	•		•	•	0.910 ± 0.005	0.539 ± 0.013	0.526 ± 0.014	0.562 ± 0.026	0.539 ± 0.013	0.969 ± 0.001
	•	•	•			0.977 ± 0.004	0.764 ± 0.017	0.747 ± 0.021	0.760 ± 0.042	0.764 ± 0.017	0.985 ± 0.001
	•	•	•		•	0.978 ± 0.002	0.772 ± 0.023	0.751 ± 0.023	0.767 ± 0.035	0.772 ± 0.023	0.985 ± 0.001
	•	•	•	•		0.979 ± 0.003	0.774 ± 0.022	0.758 ± 0.013	0.768 ± 0.001	0.774 ± 0.022	0.985 ± 0.002
	•	•	•	•	•	0.979 ± 0.004	0.770 ± 0.017	0.755 ± 0.019	0.783 ± 0.043	0.770 ± 0.017	0.985 ± 0.001
•					•	0.914 ± 0.014	0.735 ± 0.020	0.728 ± 0.024	0.760 ± 0.030	0.735 ± 0.020	0.982 ± 0.002
•				•		0.905 ± 0.020	0.717 ± 0.008	0.716 ± 0.005	0.769 ± 0.028	0.717 ± 0.008	0.981 ± 0.001
•				•	•	0.905 ± 0.011	0.707 ± 0.026	0.703 ± 0.025	0.751 ± 0.017	0.707 ± 0.026	0.980 ± 0.001
•			•			0.937 ± 0.014	0.737 ± 0.030	0.733 ± 0.030	0.774 ± 0.033	0.737 ± 0.030	0.983 ± 0.002
•			•		•	0.925 ± 0.014	0.735 ± 0.036	0.725 ± 0.034	0.775 ± 0.042	0.735 ± 0.036	0.982 ± 0.002
•			•	•		0.931 ± 0.011	0.764 ± 0.019	0.753 ± 0.018	0.769 ± 0.012	0.764 ± 0.019	0.984 ± 0.002
•			•	•	•	0.946 ± 0.014	0.756 ± 0.019	0.741 ± 0.012	0.766 ± 0.018	0.756 ± 0.019	0.984 ± 0.001
•		•				0.899 ± 0.004	0.606 ± 0.013	0.567 ± 0.003	0.582 ± 0.051	0.606 ± 0.013	0.973 ± 0.000
•		•			•	0.895 ± 0.023	0.583 ± 0.039	0.556 ± 0.061	0.578 ± 0.088	0.583 ± 0.039	0.972 ± 0.003
•		•		•		0.893 ± 0.014	0.586 ± 0.074	0.548 ± 0.071	0.549 ± 0.061	0.586 ± 0.074	0.972 ± 0.005
•		•		•	•	0.886 ± 0.016	0.571 ± 0.027	0.538 ± 0.032	0.547 ± 0.045	0.571 ± 0.027	0.970 ± 0.002
•		•	•			0.945 ± 0.007	0.727 ± 0.036	0.713 ± 0.055	0.736 ± 0.059	0.727 ± 0.036	0.982 ± 0.003
•		•	•		•	0.956 ± 0.005	0.754 ± 0.010	0.747 ± 0.014	0.776 ± 0.043	0.754 ± 0.010	0.984 ± 0.001
•		•	•	•		0.939 ± 0.004	0.712 ± 0.030	0.704 ± 0.038	0.737 ± 0.058	0.712 ± 0.030	0.981 ± 0.003
•		•	•	•	•	0.937 ± 0.025	0.694 ± 0.036	0.677 ± 0.034	0.717 ± 0.029	0.694 ± 0.036	0.980 ± 0.003
•	•					0.911 ± 0.021	0.699 ± 0.017	0.687 ± 0.020	0.725 ± 0.035	0.699 ± 0.017	0.979 ± 0.001
•	•				•	0.912 ± 0.012	0.703 ± 0.037	0.687 ± 0.044	0.723 ± 0.068	0.703 ± 0.037	0.980 ± 0.003
•	•			•		0.899 ± 0.025	0.703 ± 0.028	0.705 ± 0.033	0.753 ± 0.034	0.703 ± 0.028	0.980 ± 0.002
•	•			•	•	0.921 ± 0.016	0.709 ± 0.024	0.697 ± 0.026	0.733 ± 0.044	0.709 ± 0.024	0.980 ± 0.002
•	•		•			0.934 ± 0.011	0.747 ± 0.015	0.736 ± 0.012	0.760 ± 0.007	0.747 ± 0.015	0.983 ± 0.001
•	•		•		•	0.926 ± 0.015	0.740 ± 0.035	0.733 ± 0.036	0.762 ± 0.031	0.740 ± 0.035	0.983 ± 0.003
•	•		•	•		0.932 ± 0.013	0.737 ± 0.025	0.726 ± 0.027	0.747 ± 0.027	0.737 ± 0.025	0.982 ± 0.002
•	•		•	•	•	0.934 ± 0.018	0.755 ± 0.024	0.749 ± 0.019	0.773 ± 0.011	0.755 ± 0.024	0.984 ± 0.002
•	•	•				0.904 ± 0.018	0.613 ± 0.054	0.584 ± 0.070	0.594 ± 0.075	0.613 ± 0.054	0.974 ± 0.004
•	•	•			•	0.887 ± 0.017	0.597 ± 0.064	0.555 ± 0.070	0.558 ± 0.093	0.597 ± 0.064	0.972 ± 0.005
•	•	•		•		0.902 ± 0.021	0.598 ± 0.026	0.552 ± 0.049	0.551 ± 0.076	0.598 ± 0.026	0.973 ± 0.003
•	•	•		•	•	0.898 ± 0.026	0.596 ± 0.039	0.574 ± 0.035	0.584 ± 0.042	0.596 ± 0.039	0.973 ± 0.002
•	•	•	•			0.947 ± 0.012	0.728 ± 0.021	0.722 ± 0.023	0.744 ± 0.027	0.728 ± 0.021	0.982 ± 0.001
•	•	•	•		•	0.943 ± 0.007	0.713 ± 0.020	0.695 ± 0.032	0.719 ± 0.037	0.713 ± 0.020	0.981 ± 0.002
•	•	•	•	•		0.936 ± 0.017	0.696 ± 0.057	0.673 ± 0.059	0.690 ± 0.048	0.696 ± 0.057	0.979 ± 0.004
•	•	•	•	•	•	0.945 ± 0.005	0.744 ± 0.025	0.735 ± 0.022	0.756 ± 0.037	0.744 ± 0.025	0.983 ± 0.001

Table S21: Classification metrics for MLP IF with different omics combination on CCLE dataset.

mRNA	miRNA	CNV	DNAm	Protein	Metabolomics	AUROC	Accuracy	F1	Precision	Recall	Specificity
				•	•	0.997 ± 0.003	0.954 ± 0.010	0.954 ± 0.012	0.963 ± 0.012	0.954 ± 0.010	0.997 ± 0.001
			•		•	0.998 ± 0.002	0.964 ± 0.013	0.964 ± 0.014	0.969 ± 0.011	0.964 ± 0.013	0.997 ± 0.001
			•	•		0.996 ± 0.003	0.955 ± 0.011	0.955 ± 0.012	0.961 ± 0.013	0.955 ± 0.011	0.997 ± 0.001
			•	•	•	0.997 ± 0.002	0.945 ± 0.028	0.946 ± 0.028	0.955 ± 0.025	0.945 ± 0.028	0.996 ± 0.002
		•			•	0.995 ± 0.002	0.919 ± 0.028	0.922 ± 0.028	0.932 ± 0.028	0.919 ± 0.028	0.994 ± 0.002
		•		•		0.988 ± 0.002	0.876 ± 0.023	0.873 ± 0.021	0.898 ± 0.024	0.876 ± 0.023	0.992 ± 0.001
		•		•	•	0.999 ± 0.000	0.950 ± 0.018	0.950 ± 0.018	0.964 ± 0.012	0.950 ± 0.018	0.996 ± 0.001
		•	•			0.991 ± 0.007	0.926 ± 0.013	0.925 ± 0.015	0.935 ± 0.014	0.926 ± 0.013	0.995 ± 0.001
		•	•		•	0.996 ± 0.002	0.938 ± 0.013	0.935 ± 0.014	0.941 ± 0.014	0.938 ± 0.013	0.995 ± 0.001
		•	•	•		0.995 ± 0.003	0.948 ± 0.011	0.948 ± 0.013	0.958 ± 0.011	0.948 ± 0.011	0.996 ± 0.001
		•	•	•	•	0.994 ± 0.005	0.946 ± 0.016	0.944 ± 0.017	0.954 ± 0.013	0.946 ± 0.016	0.996 ± 0.001
	•				•	0.995 ± 0.001	0.918 ± 0.013	0.923 ± 0.012	0.937 ± 0.011	0.918 ± 0.013	0.994 ± 0.001
	•			•		0.993 ± 0.001	0.883 ± 0.013	0.883 ± 0.012	0.909 ± 0.015	0.883 ± 0.013	0.992 ± 0.001
	•			•	•	0.998 ± 0.001	0.958 ± 0.005	0.958 ± 0.003	0.964 ± 0.005	0.958 ± 0.005	0.997 ± 0.000
	•		•			0.994 ± 0.006	0.940 ± 0.019	0.939 ± 0.021	0.947 ± 0.020	0.940 ± 0.019	0.996 ± 0.001
	•		•		•	0.995 ± 0.003	0.945 ± 0.017	0.943 ± 0.018	0.950 ± 0.016	0.945 ± 0.017	0.996 ± 0.001
	•		•	•		0.997 ± 0.002	0.961 ± 0.017	0.962 ± 0.017	0.970 ± 0.012	0.961 ± 0.017	0.997 ± 0.001
	•		•	•	•	0.998 ± 0.002	0.973 ± 0.015	0.972 ± 0.018	0.974 ± 0.017	0.973 ± 0.015	0.998 ± 0.001
	•	•				0.988 ± 0.003	0.872 ± 0.015	0.875 ± 0.016	0.905 ± 0.013	0.872 ± 0.015	0.992 ± 0.001
	•	•			•	0.996 ± 0.003	0.926 ± 0.019	0.930 ± 0.020	0.949 ± 0.017	0.926 ± 0.019	0.995 ± 0.001
	•	•		•		0.995 ± 0.003	0.886 ± 0.025	0.889 ± 0.027	0.917 ± 0.019	0.886 ± 0.025	0.993 ± 0.002
	•	•		•	•	0.999 ± 0.001	0.938 ± 0.016	0.943 ± 0.016	0.958 ± 0.012	0.938 ± 0.016	0.996 ± 0.001
	•	•	•			0.994 ± 0.003	0.929 ± 0.017	0.926 ± 0.020	0.940 ± 0.021	0.929 ± 0.017	0.995 ± 0.001
	•	•	•		•	0.998 ± 0.002	0.949 ± 0.014	0.948 ± 0.017	0.959 ± 0.014	0.949 ± 0.014	0.996 ± 0.001
	•	•	•	•		0.996 ± 0.003	0.956 ± 0.015	0.955 ± 0.016	0.964 ± 0.012	0.956 ± 0.015	0.997 ± 0.001
	•	•	•	•	•	0.997 ± 0.002	0.956 ± 0.008	0.955 ± 0.008	0.963 ± 0.007	0.956 ± 0.008	0.997 ± 0.001
•					•	0.912 ± 0.014	0.764 ± 0.040	0.757 ± 0.041	0.810 ± 0.023	0.764 ± 0.040	0.984 ± 0.003
•				•		0.908 ± 0.028	0.755 ± 0.059	0.749 ± 0.065	0.812 ± 0.044	0.755 ± 0.059	0.983 ± 0.004
•				•	•	0.914 ± 0.023	0.747 ± 0.026	0.740 ± 0.023	0.797 ± 0.009	0.747 ± 0.026	0.984 ± 0.001
•			•			0.942 ± 0.016	0.856 ± 0.031	0.853 ± 0.026	0.887 ± 0.008	0.856 ± 0.031	0.991 ± 0.002
•			•		•	0.937 ± 0.020	0.868 ± 0.030	0.868 ± 0.022	0.900 ± 0.017	0.868 ± 0.030	0.991 ± 0.002
•			•	•		0.927 ± 0.009	0.834 ± 0.014	0.831 ± 0.015	0.875 ± 0.014	0.834 ± 0.014	0.989 ± 0.001
•			•	•	•	0.940 ± 0.011	0.880 ± 0.038	0.875 ± 0.029	0.895 ± 0.020	0.880 ± 0.038	0.992 ± 0.002
•		•				0.910 ± 0.013	0.754 ± 0.029	0.746 ± 0.034	0.791 ± 0.045	0.754 ± 0.029	0.983 ± 0.002
•		•			•	0.920 ± 0.015	0.769 ± 0.023	0.757 ± 0.025	0.799 ± 0.037	0.769 ± 0.023	0.985 ± 0.001
•		•		•		0.922 ± 0.022	0.780 ± 0.026	0.772 ± 0.024	0.813 ± 0.026	0.780 ± 0.026	0.986 ± 0.001
•		•		•	•	0.917 ± 0.017	0.809 ± 0.022	0.807 ± 0.028	0.848 ± 0.035	0.809 ± 0.022	0.987 ± 0.002
•		•	•			0.939 ± 0.015	0.859 ± 0.031	0.856 ± 0.029	0.882 ± 0.026	0.859 ± 0.031	0.991 ± 0.002
•		•	•		•	0.939 ± 0.007	0.872 ± 0.029	0.867 ± 0.023	0.895 ± 0.012	0.872 ± 0.029	0.991 ± 0.001
•		•	•	•		0.958 ± 0.011	0.894 ± 0.032	0.887 ± 0.031	0.907 ± 0.022	0.894 ± 0.032	0.993 ± 0.002

Continued on next page

Table S21: Classification metrics for MLP IF with different omics combination on CCLE dataset.

mRNA	miRNA	CNV	DNAm	Protein	Metabolomics	AUROC	Accuracy	F1	Precision	Recall	Specificity
•		•	•	•	•	0.955 ± 0.011	0.896 ± 0.026	0.892 ± 0.024	0.912 ± 0.010	0.896 ± 0.026	0.993 ± 0.001
•	•					0.908 ± 0.015	0.761 ± 0.012	0.756 ± 0.007	0.806 ± 0.008	0.761 ± 0.012	0.984 ± 0.001
•	•				•	0.919 ± 0.026	0.794 ± 0.041	0.787 ± 0.040	0.820 ± 0.032	0.794 ± 0.041	0.986 ± 0.003
•	•			•		0.915 ± 0.015	0.771 ± 0.024	0.752 ± 0.025	0.806 ± 0.021	0.771 ± 0.024	0.984 ± 0.001
•	•			•	•	0.922 ± 0.018	0.801 ± 0.009	0.798 ± 0.010	0.837 ± 0.012	0.801 ± 0.009	0.987 ± 0.001
•	•		•			0.931 ± 0.013	0.864 ± 0.025	0.867 ± 0.022	0.901 ± 0.022	0.864 ± 0.025	0.991 ± 0.002
•	•		•		•	0.943 ± 0.014	0.869 ± 0.039	0.864 ± 0.038	0.893 ± 0.036	0.869 ± 0.039	0.991 ± 0.003
•	•		•	•		0.940 ± 0.005	0.858 ± 0.025	0.853 ± 0.021	0.878 ± 0.012	0.858 ± 0.025	0.991 ± 0.001
•	•		•	•	•	0.948 ± 0.007	0.883 ± 0.028	0.875 ± 0.021	0.897 ± 0.015	0.883 ± 0.028	0.992 ± 0.002
•	•	•				0.917 ± 0.011	0.785 ± 0.017	0.777 ± 0.015	0.818 ± 0.025	0.785 ± 0.017	0.986 ± 0.001
•	•	•			•	0.922 ± 0.021	0.794 ± 0.025	0.787 ± 0.021	0.829 ± 0.026	0.794 ± 0.025	0.986 ± 0.002
•	•	•		•		0.918 ± 0.016	0.805 ± 0.043	0.791 ± 0.041	0.821 ± 0.030	0.805 ± 0.043	0.987 ± 0.003
•	•	•		•	•	0.933 ± 0.020	0.823 ± 0.065	0.819 ± 0.062	0.860 ± 0.037	0.823 ± 0.065	0.988 ± 0.004
•	•	•	•			0.955 ± 0.012	0.872 ± 0.030	0.865 ± 0.029	0.884 ± 0.029	0.872 ± 0.030	0.991 ± 0.002
•	•	•	•		•	0.948 ± 0.008	0.890 ± 0.022	0.889 ± 0.020	0.916 ± 0.009	0.890 ± 0.022	0.993 ± 0.001
•	•	•	•	•		0.942 ± 0.016	0.885 ± 0.029	0.884 ± 0.022	0.912 ± 0.015	0.885 ± 0.029	0.992 ± 0.002
•	•	•	•	•	•	0.956 ± 0.012	0.900 ± 0.020	0.898 ± 0.022	0.916 ± 0.020	0.900 ± 0.020	0.994 ± 0.001

Table S22: Classification metrics for MOGONET with different omics combination on CCLE dataset.

mRNA	miRNA	CNV	DNAm	Protein	Metabolomics	AUROC	Accuracy	F1	Precision	Recall	Specificity
				•	•	0.878 ± 0.012	0.483 ± 0.036	0.470 ± 0.035	0.491 ± 0.049	0.483 ± 0.036	0.967 ± 0.003
			•		•	0.913 ± 0.009	0.558 ± 0.019	0.549 ± 0.023	0.586 ± 0.029	0.558 ± 0.019	0.972 ± 0.001
			•	•		0.935 ± 0.007	0.598 ± 0.015	0.597 ± 0.025	0.667 ± 0.054	0.598 ± 0.015	0.974 ± 0.001
			•	•	•	0.901 ± 0.012	0.633 ± 0.046	0.616 ± 0.040	0.657 ± 0.032	0.633 ± 0.046	0.977 ± 0.003
		•			•	0.842 ± 0.008	0.351 ± 0.048	0.345 ± 0.044	0.381 ± 0.060	0.351 ± 0.048	0.959 ± 0.002
		•		•		0.889 ± 0.004	0.451 ± 0.059	0.449 ± 0.059	0.514 ± 0.082	0.451 ± 0.059	0.964 ± 0.003
		•		•	•	0.876 ± 0.015	0.550 ± 0.033	0.531 ± 0.037	0.556 ± 0.049	0.550 ± 0.033	0.971 ± 0.001
		•	•			0.945 ± 0.003	0.666 ± 0.043	0.648 ± 0.046	0.680 ± 0.049	0.666 ± 0.043	0.977 ± 0.002
		•	•		•	0.920 ± 0.017	0.620 ± 0.056	0.598 ± 0.052	0.623 ± 0.041	0.620 ± 0.056	0.975 ± 0.003
		•	•	•		0.938 ± 0.009	0.664 ± 0.022	0.653 ± 0.031	0.698 ± 0.048	0.664 ± 0.022	0.978 ± 0.002
	•				•	0.860 ± 0.013	0.454 ± 0.028	0.443 ± 0.021	0.480 ± 0.025	0.454 ± 0.028	0.963 ± 0.002
	•			•		0.865 ± 0.005	0.513 ± 0.047	0.501 ± 0.048	0.531 ± 0.066	0.513 ± 0.047	0.968 ± 0.002
	•			•	•	0.862 ± 0.022	0.514 ± 0.033	0.491 ± 0.030	0.508 ± 0.043	0.514 ± 0.033	0.968 ± 0.002
	•		•			0.918 ± 0.005	0.636 ± 0.051	0.621 ± 0.052	0.636 ± 0.048	0.636 ± 0.051	0.974 ± 0.003
	•		•		•	0.903 ± 0.007	0.604 ± 0.037	0.590 ± 0.035	0.632 ± 0.040	0.604 ± 0.037	0.972 ± 0.003
	•		•	•		0.905 ± 0.024	0.613 ± 0.009	0.589 ± 0.009	0.621 ± 0.048	0.613 ± 0.009	0.974 ± 0.001
	•	•				0.867 ± 0.003	0.481 ± 0.024	0.455 ± 0.011	0.497 ± 0.037	0.481 ± 0.024	0.964 ± 0.002

Continued on next page

Table S22: Classification metrics for MOGONET with different omics combination on CCLE dataset.

mRNA	miRNA	CNV	DNAm	Protein	Metabolomics	AUROC	Accuracy	F1	Precision	Recall	Specificity
	•	•			•	0.837 ± 0.013	0.497 ± 0.017	0.468 ± 0.031	0.478 ± 0.034	0.497 ± 0.017	0.965 ± 0.001
	•	•		•		0.872 ± 0.013	0.547 ± 0.018	0.536 ± 0.018	0.591 ± 0.020	0.547 ± 0.018	0.968 ± 0.001
	•	•	•			0.915 ± 0.015	0.638 ± 0.036	0.619 ± 0.041	0.649 ± 0.039	0.638 ± 0.036	0.974 ± 0.002
•					•	0.884 ± 0.011	0.514 ± 0.045	0.518 ± 0.041	0.577 ± 0.022	0.514 ± 0.045	0.969 ± 0.002
•				•		0.900 ± 0.009	0.539 ± 0.025	0.538 ± 0.021	0.599 ± 0.019	0.539 ± 0.025	0.970 ± 0.001
•				•	•	0.881 ± 0.027	0.594 ± 0.047	0.581 ± 0.040	0.620 ± 0.026	0.594 ± 0.047	0.974 ± 0.003
•			•			0.932 ± 0.024	0.712 ± 0.058	0.688 ± 0.057	0.720 ± 0.056	0.712 ± 0.058	0.980 ± 0.003
•			•		•	0.918 ± 0.009	0.657 ± 0.021	0.647 ± 0.010	0.689 ± 0.005	0.657 ± 0.021	0.977 ± 0.001
•			•	•		0.919 ± 0.012	0.663 ± 0.030	0.649 ± 0.023	0.722 ± 0.045	0.663 ± 0.030	0.977 ± 0.002
•		•				0.898 ± 0.012	0.566 ± 0.025	0.555 ± 0.015	0.618 ± 0.038	0.566 ± 0.025	0.971 ± 0.001
•		•			•	0.877 ± 0.021	0.565 ± 0.069	0.557 ± 0.061	0.595 ± 0.040	0.565 ± 0.069	0.972 ± 0.004
•		•		•		0.895 ± 0.019	0.602 ± 0.040	0.588 ± 0.033	0.622 ± 0.030	0.602 ± 0.040	0.973 ± 0.002
•		•	•			0.938 ± 0.009	0.688 ± 0.054	0.664 ± 0.053	0.691 ± 0.052	0.688 ± 0.054	0.979 ± 0.003
•	•					0.889 ± 0.009	0.572 ± 0.041	0.567 ± 0.045	0.598 ± 0.056	0.572 ± 0.041	0.971 ± 0.003
•	•				•	0.871 ± 0.030	0.563 ± 0.048	0.564 ± 0.045	0.607 ± 0.044	0.563 ± 0.048	0.971 ± 0.003
•	•			•		0.872 ± 0.028	0.600 ± 0.033	0.589 ± 0.033	0.638 ± 0.031	0.600 ± 0.033	0.973 ± 0.002
•	•		•			0.904 ± 0.007	0.660 ± 0.037	0.648 ± 0.039	0.672 ± 0.033	0.660 ± 0.037	0.976 ± 0.003
•	•	•				0.880 ± 0.009	0.596 ± 0.031	0.579 ± 0.038	0.609 ± 0.045	0.596 ± 0.031	0.972 ± 0.002
